# Benchmarking the electrochemical parameters of the LiNi_0.8_Mn_0.1_Co_0.1_O_2_ positive electrode material for Li-ion batteries

**DOI:** 10.1016/j.heliyon.2023.e21881

**Published:** 2023-11-01

**Authors:** Aleksandra A. Savina, Artem M. Abakumov

**Affiliations:** Center for Energy Science and Technology, Skolkovo Institute of Science and Technology, Bolshoy Boulevard 30, bld. 1, 121205, Moscow, Russia

**Keywords:** Li-ion battery, Cathode material, Ni-rich layered oxide, NMC811, Benchmarking

## Abstract

The layered oxide LiNi_0.8_Mn_0.1_Co_0.1_O_2_ (NMC811, NCM811) is of utmost technological importance as a positive electrode (cathode) material for the forthcoming generation of Li-ion batteries. In this contribution, we have collected 548 research articles comprising >950 records on the electrochemical properties of NMC811 as a cathode material in half-cells with metallic Li counter electrode. The analysis of distribution histograms provided statistically-relevant values of such key characteristics of NMC811 as the first cycle discharge capacity and Coulombic efficiency, discharge capacities at different upper cut-off voltages, capacity fade and capacity retention at the 0.1C–5C current densities. We derived equations describing the relationships between discharge capacity and upper cut-off voltage, Ni content in the LiNi_x_Mn_y_Co_z_O_2_ compositions in vicinity of NMC811, antisite disorder, and the C-rate. Additionally, the distribution histograms were used for a qualitative comparison between various groups of NMC811 materials, such as benchmarks in various optimizations vs obtained in course of synthesis development, lab-made vs commercial, polycrystalline vs single-crystal. The results of this analysis provide justified values to be used as benchmarks in further works related to optimizing and improving NMC811 and related materials, eliminating random picking up from a huge pool of published data.

## 1 Introduction

**Fig. 1:**
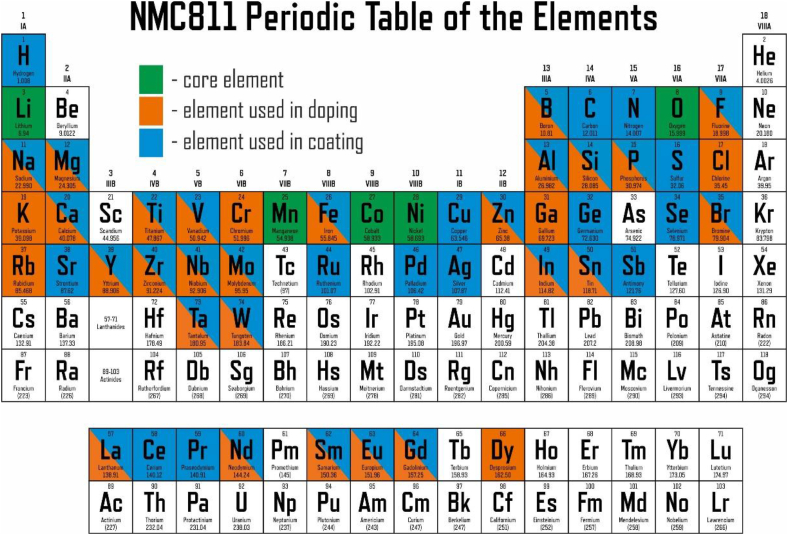
Chemical elements utilized in various doping and coating strategies targeting improvement of the functional properties of NMC811 cathode materials, based on the data from 381 publications from the years of 2009–2023.

Complex layered oxides of lithium and transition metals LiNi_x_Mn_y_Co_z_O_2_ (x + y + z = 1, also termed NMCXYZ) are widely commercialized positive electrode (cathode) materials for Li-ion batteries powering portable electronic devices, electric cars, unmanned aerial vehicles, electric tools, and demonstrating sustainably growing market within last decade. The commonly recognized technology trend resides in switching to the cathode materials with increasing nickel content that provide higher energy density at reduced cost due to diminishing concentration of rather expensive and sparse cobalt. On this way, the material of the first generation, LiCoO_2_, with the practical electrochemical capacity of ∼140 mAh/g is gradually being replaced with the second-generation materials NMC111 (C ≈ 160 mAh/g) and NMC532 (C ≈ 170 mAh/g), and later with the third-generation material NMC622 delivering the capacity of about 180 mAh/g (as referred to the similar electrochemical cycling conditions) [[1], [2], [3]]. Nowadays the industry turns towards mass production of the battery cells based on the cathode with even higher Ni content, NMC811, with the reversible electrochemical capacity of ∼200 mAh/g and the energy density approaching 750 Wh/kg, allowing for the energy density at the cell level of 300 Wh/kg [2]. Although the market is still occupied by the materials of the second and third generations, the share of NMC811 increases drastically within last few years, predominantly at the cost of NMC532 [4]. This makes NMC811 one of the primary targets for the material science R&D activity related to Li-ion batteries. Hundreds of research articles have been published so far on the synthesis techniques, microstructure optimization, design of core-shell and compositional gradient structures, chemical doping and protective coating of NMC811. The variety of chemical elements utilized in various doping and coating strategies amounts to 51 out of 81 elements of the Periodic Table (excluding the radioactive ones) (Fig. 1).

Nevertheless, the material science community still operates rather imprecise and incomplete information even on the basic electrochemical parameters of NMC811, despite they have been extensively studied and reported many times. For instance, the electrochemical discharge capacity of NMC811 is generally alluded to ∼200 mAh/g (referring to the measurements in half-cells with Li-metal anode at the upper voltage limit of 4.3 V vs Li/Li^+^ and the 0.1C rate). However, even at these charge/discharge conditions, one can find the reported first discharge capacity values of NMC811 varying in the range of ∼160–230 mAh/g, thus posing the legitimate questions on how the value of 200 mAh/g has been selected and how trustworthy this value is. Much less is known on other essential aspects of the NMC811 electrochemical behavior, such as first cycle Coulombic efficiency, rate capability and capacity retention. Due to natural space and time limitations, only a little fraction of the reported data is usually analyzed and summarized in review articles that might be sufficient for picking up qualitative trends, but does not allow for reliable quantitative estimates [[5], [6], [7], [8], [9], [10], [11]]. Particularly, quantitative correlations between various parameters (such as discharge capacity vs voltage range or capacity retention vs C-rate) are lacking.

The basic electrochemical properties of NMC811 have been reported many times either as the primary target or as a benchmark for further improvement. The main objective of this study is to analyze this pool of information and to retrieve statistically-relevant estimates of the most important parameters and trace their mutual influence, as well as their dependencies on chemistry, crystal structure and morphology. The data from >500 publications on NMC811 allowed us to find out these quantitative measures, which can further serve for benchmarking if the optimization and improvement of NMC811 cathodes are targeted. We also derived a set of empirical equations interconnecting various electrochemical, compositional and structural variables that provide a playground for further theoretical and computational examination of these correlations. Finally, the performed analysis illustrates how coherent the electrochemical and material science society is in measuring the key properties of the electrode materials.

## 2 Methodology

A collection of 548 articles published in 2005–2023 has been screened to retrieve the data on the NMC811 cathode material that include:

- electrochemical capacity at first discharge, C (mAh/g);
- first cycle Coulombic efficiency, FCE (%, measured as the ratio between the capacities on first discharge and charge);
- upper, E_U_, and lower, E_L_, potential limits for the charge/discharge cycling (V vs. Li/Li^+^);
- current density, expressed as C-rate, CR (1C = 200 mA/g);
- capacity fade CF at given E_U_ and CR (% per cycle);
- discharge capacity at the CRs of 0.1C, 0.2C, 0.5C, 1C, 2C and 5C;
- chemical composition;
- concentration of antisite defects g_Ni_ (%, measured as Ni fraction in the Li site of the *R*-3*m* α-NaFeO_2_-type crystal structure);
- positive electrode composition in weight % of the active material, conductive additive, and binder.

The numerical data were read from the text and tables, if available, or measured from corresponding graphs. Only the electrochemical data measured with galvanostatic cycling with potential limitations at room temperature in half-cells with Li metallic anode and liquid electrolytes based on LiPF_6_ solution (typically with 1 M concentration) in alkyl carbonate-based solvents were collected. The data on full cells or other types of electrochemical systems (such as all-solid-state cells) were not included in the analysis. The range of considered chemical compositions spans over the Ni content corresponding to 0.75≤x ≤ 0.85 in the LiNi_x_Mn_y_Co_z_O_2_ formula.

Each set of numerical values has been supported with the following metadata:

- “polycrystalline” - the material in a form of separate or agglomerated submicron primary particles;
- “single-crystal” - the material in a form of separate crystals of few microns in size;
- “benchmark” - the pristine material used as benchmark for further optimization (typically doping, coating, core-shell or gradient structures);
- “synthesis” – the material obtained in course of development or optimization of a certain synthesis technique;
- “lab-made” – the material synthesized as a part of the conducted research. Even if the preparation was started from a commercial precursor, but last step(s) was performed in the lab, the material was considered “lab-made”;
- “commercial” – the material was received from an enterprise and used as-received.

In total, the whole dataset comprises 951 entries. The numerical data, extracted from literature, are provided in Supporting Information along with Digital Object Identifiers (10.13039/100000201DOI) of the source publications. The analysis of these data was performed with distribution histograms [12]. The quantitative measures of the first cycle discharge capacity and Coulombic efficiency, capacity fade and discharge capacitis at various C-rate were determined either by fitting the histograms with Gaussian peaks or by calculating the median values. Finally, in order to ensure the coherency of the calculated parameters (i.e. that all the values can be realized simultaneously in one and the same sample), we have synthesized a single NMC811 specimen using the most common co-precipitation technique and measured all abovementioned parameters experimentally (see Supporting Information for the experimental details and results).

## 3 Results and discussion

In the analyzed articles, the reported initial discharge capacity of NMC811 has been tested at the upper voltage limits in the range of 4.2–4.8 V vs Li/Li^+^ and at different current rates, but the most frequent measurements refer to E_U_ = 4.3 V and CR = 0.1C. The capacities formally measured at the same C-rate are, in fact, split into two large groups, in which 1C corresponds to close but different current densities of 200 mA/g and 180 mA/g. Thus, before constructing the data pool for further analysis, the distributions of discharge capacities at the current densities of 20 mA/g and 18 mA/g were compared (Fig. S1appsec1 of Supporting Information). In both cases, the distributions are heavily skewed (see more detailed analysis below) but centered to the same values, particularly if compared to the huge spread of the capacity values. Due to negligible difference between the two distributions, they were combined and treated together resulting in 311 reported discharge capacities.

**Fig. 2:**
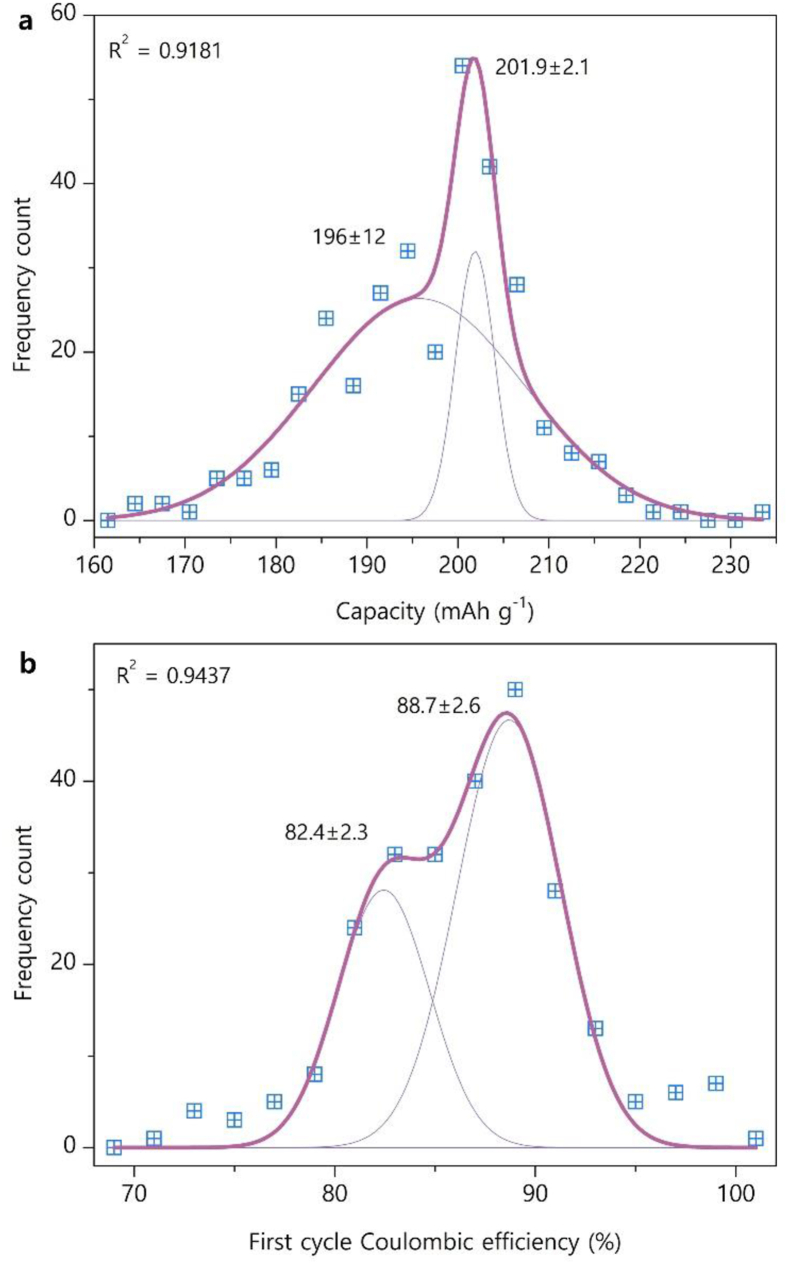
Distribution histograms of first discharge capacity (a) and first cycle Coulombic efficiency (b) at *E*_U_ = 4.3 V and C-rate of 0.1C. The distributions are fitted with two Gaussian peaks (shown as faint lines). The mean values and standard deviations are marked. Adjusted R^2^ coefficients are provided as a goodness of fit measures.

The distribution of the initial discharge capacity is negatively skewed, with a peak at ∼200 mAh/g and pronounced shoulder at nearly ∼195 mAh/g (Fig. 2a). The distribution can be fitted with two Gaussian peaks with drastically different full-width-at-half-maximum (FWHM): the narrow and broad peaks are characterized with the mean capacity values ${\bar{C}}_{1}$ = 201.9 ± 2.1 mAh/g and ${\bar{C}}_{2}$ = 196 ± 12 mAh/g, respectively. Similar asymmetric shape is realized for the distribution of FCE (Fig. 2b), with the peaks at ${\bar{\text{FCE}}}_{1}$ = 88.7 ± 2.6 % and ${\bar{\text{FCE}}}_{2}$ = 82.4 ± 2.3 %.

From the Central Limit Theorem, the negatively skewed distribution cannot arise from independent measurements with fully random errors. Nevertheless, the observed asymmetry can be rationalized from general considerations. At low current density minimizing the impact of kinetic and diffusion limitations, the maximum capacity is defined by the capacity of the electronic bands corresponding to the Ni^2+^/Ni^3+^, Ni^3+^/Ni^4+^ and Co^3+^/Co^4+^ redox pairs within the given potential window. This in turn depends on the position of the Fermi level within the hybridized Co3*d*-O2*p* band which is emptied at the end of charge [13,14]. The number of electrons which the bands can donate is determined by the chemical composition and crystal structure which can be considered the same for all samples in the analyzed pool. It is legitimate to assume that the Gaussian peak at the right side of the capacity histogram provides a measure of the electronic capacity of the bands broadened due to random experimental errors according to the normal distribution. Small standard deviation of ∼1 % confirms that this limiting value is measured reproducibly. Thus, the 201.9 ± 2.1 mAh/g mean value of this normal distribution can be accepted as a statistically-relevant benchmark for the NMC811 first cycle discharge capacity measured at *E*_U_ = 4.3 V and CR = 0.1C in Li half-cell. The broad peak in the capacity histogram spans between 163 and 232 mAh/g; both values do not look realistic for NMC811 at the given cycling conditions. Large variance (measured as squared standard deviation which amounts to >6 % of the mean value) indicates that this peak accumulates significant experimental errors, which might reside in imprecisions in the active material mass per unit area of the cathode, in off-setting of the potential limits and current density, or in imprecise characterization of the chemical composition. In order to evaluate the impact of the electrode composition on the first discharge capacity, its distribution histogram has been constructed from the data measured on the electrodes with the 80:10:10 wt% ratio of the active material, conductive additive and binder (∼74 % of the reported discharge capacities at E_U_ = 4.3 V and CR = 0.1C). The histogram at the constant electrode composition (Fig. S2a) is negatively skewed with the sharp peak at ∼202 mAh/g, similarly to the distribution histogram of the full dataset in Fig. 2a. This indicates that the skewness and bimodal appearance of the distribution histogram of the first discharge capacity is not nested in the difference in the electrode compositions. The median and mean values of first discharge capacity change only slightly with the active material content in the electrode composition (Figs. S2b and c). These changes are negligible compared to standard deviations taken as a measure of data variance (Fig. S2c) that prevents their reliable analysis.

**Fig. 3:**
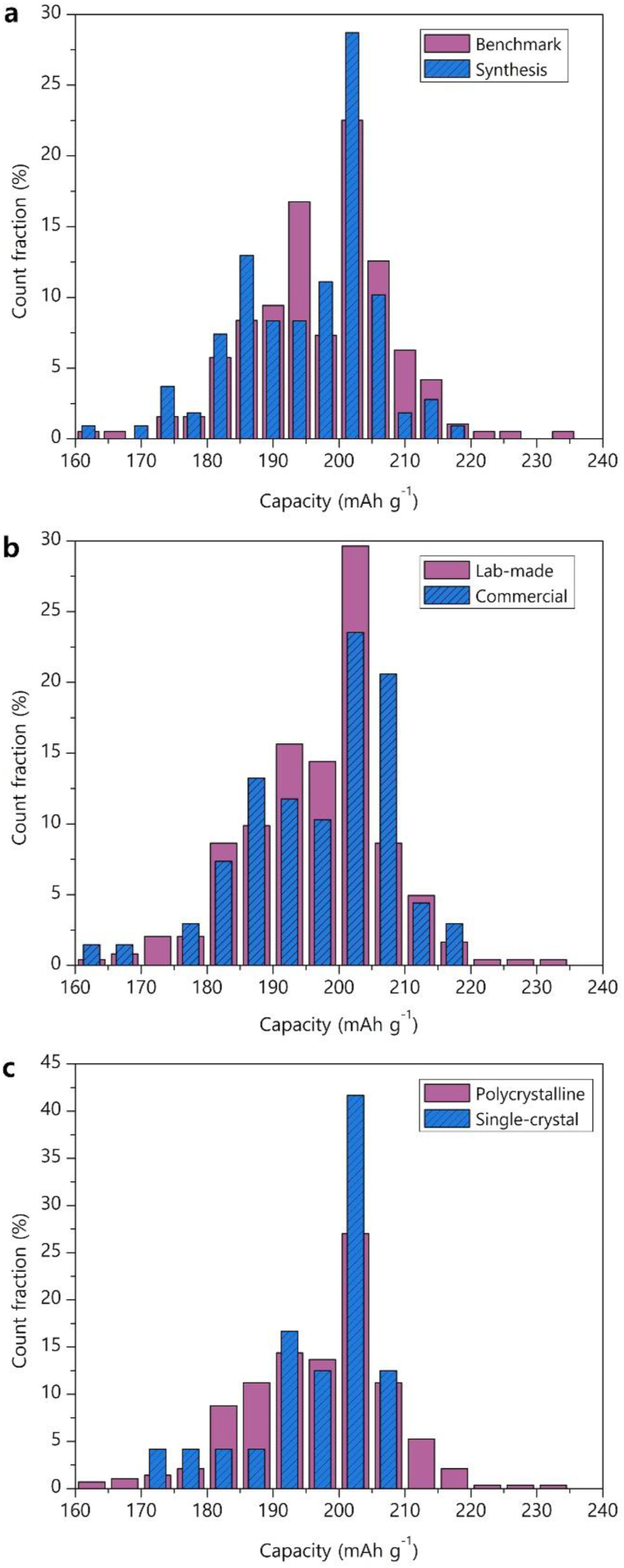
Distribution histograms of discharge capacities classified over various metadata: “benchmark” vs “synthesis” (a), “lab-made” vs “commercial” (b) and “polycrystalline” vs “single-crystal” (c).

The displacement of the mean for the broad Gaussian peak towards lower capacity value may reflect generally lower quality of the tested NMC811 materials. The exact origins of such lowering are hard to establish because of lack of fine experimental details in vast majority of publications, but one can speculate on deterioration of NMC811 due to prolonged storage and/or excessive contact with air and moisture [15,16], deviation in the synthesis procedure resulting in surface passivation with antisite defects [17,18], improper microstructure or electrode/cell preparation rendering a fraction of the material electrochemically inactive. At least partially, the origins of this negative skewness can be understood from the qualitative comparative analysis of the partial distributions constructed according to the assigned metadata (Fig. 3). The “benchmark” and “synthesis” histograms are different in the ratio of the capacity measurements centered at ∼202 and 196 mAh/g (Fig. 3a): the fraction of the former is clearly higher for the “synthesis” histogram. Tentatively, it corroborates the conjecture on the influence of sample handling on the capacity measurements. One can reasonably assume that the materials obtained in course of the synthesis optimization are electrochemically tested within short time after the preparation in order to get immediate feedback, whereas the materials for benchmarking could be stored for much longer time before being measured as they are not the primary target of the research. Unfortunately, research bias cannot also be fully excluded: if the goal is a new or optimized synthesis procedure, much efforts will be invested in producing the material with the top and competitive properties, whereas it is easier to select a benchmark with moderate performance to demonstrate further noticeable improvement. The assumption on sample deterioration due to improper storage or handling is also supported by a comparison of the “lab-made” and “commercial” histograms (Fig. 3b). Indeed, the “commercial” samples expectably demonstrate a larger fraction of the measurements between 205 and 210 mAh/g, but a significant peak at lower capacity is present for this distribution as well. This is somewhat surprising, as the commercial vendors are not expected to deliver the NMC811 materials with the discharge capacity of <200 mAh/g, losing the main advantage of high Ni content. Finally, much larger fraction of the measurements centered at ∼202 mAh/g for the “single-crystal” vs “polycrystalline” samples (Fig. 3c) also points to substantial influence of degradation which occurs primarily at the surface of the NMC811 particles, as the single-crystal samples generally demonstrate lower specific surface compared to the polycrystalline ones.

**Fig. 4:**
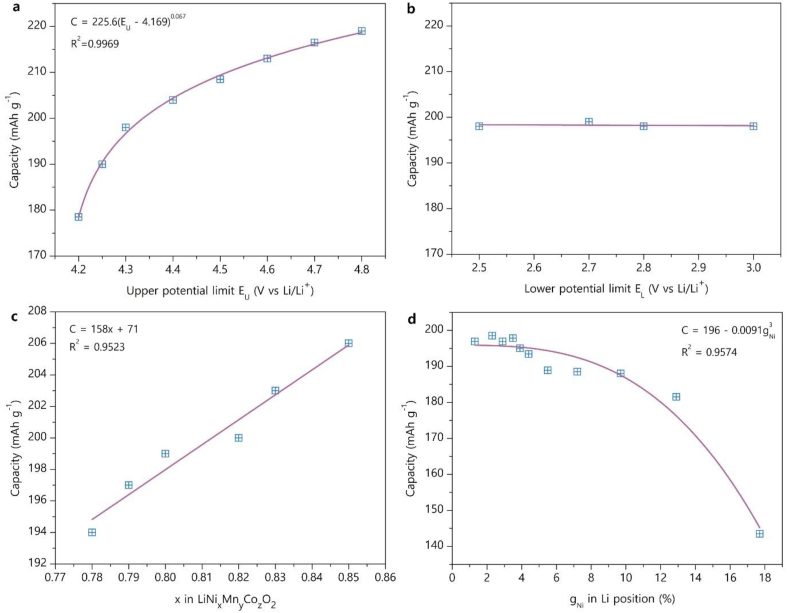
First cycle discharge capacity as a function of the upper potential limit E_U_ (a), lower potential limit E_L_ (b), overall Ni content x in the LiNi_x_Mn_y_Co_z_O_2_ formula (c) and Ni occupancy factor in the Li site g_Ni_ (d). Adjusted R^2^ coefficients are provided as a goodness of fit measures for the panels a, c, d. The line in the panel b is an eye guide.

For further analysis of the influence of various parameters on the discharge capacity the skewed capacity histograms will be characterized with their median values, as the number of available data points is often insufficient for a meaningful fitting with the Gaussian peaks. The median provides a better representation of a skewed distribution and, indeed, for the histogram in Fig. 3a the median value of 199 mAh/g is closer to the center of the narrow Gaussian peak at 202 mAh/g than the mean value of 196 ± 11 mAh/g. Thus, despite small offset towards lower capacity, the medians were used to deduce the dependence of the initial discharge capacity on upper and lower potential limits, overall Ni content and Ni fraction in the Li site (as a measure of antisite disorder) (Fig. 4).

The first cycle discharge capacity depends monotonically on the upper potential limit E_U_ within the range of 4.2 ≤ E_U_ ≤ 4.8 V vs Li/Li^+^ (Fig. 4a). This dependence can be approximated very well with the equation:

(1) C = *a*(E_U_ – *b*)^*c*^

where *a* = 225.6 ± 0.7, *b* = 4.169 ± 0.006, *c* = 0.067 ± 0.004. The equation (1) for first glance might look physically meaningless as it predicts zero capacity when E_U_ = *b*, whereas it is well known that NMC811 demonstrates significant electrochemical capacity even below 4.0 V. One should refer to the cyclic voltammogram or differential capacity plots of NMC811 to realize that equation (1) does not describe the reduction peaks at ∼3.7 and 4.0 V due to the H1↔M and M↔H2 phase transitions, but provides the capacity variation with voltage for the H2↔H3 reduction peak above 4.1 V that is considered as an instability region for NMC811 [19,20]. The limiting E_U_ value of 4.169 ± 0.006 V from equation (1) corresponds very well to the reported 4.15–4.18 V potential of the H2↔H3 reduction peak [[21], [22], [23], [24]]. In drastic contrast to clear variation of discharge capacity with the upper potential limit, it practically does not depend on the lower potential limit E_L_ in the range of 2.5–3.0 V (Fig. 4b) that agrees with absence of redox activity in this potential region.

The dependence of first discharge capacity on the Ni content x in the LiNi_x_Mn_y_Co_z_O_2_ formula within the compositional region near NMC811 (0.78 ≤ x ≤ 0.85) was fitted linearly (Fig. 4c) with the slope of 158 ± 16 mAh/g and intercept of 71 ± 13 mAh/g. Fig. S3a compares the result of this fit with the C-x dependencies reported earlier for NMCs in a broader compositional range. If linearly fitted, these data demonstrate large variance of the slope and intercept; some data significantly deviate from linearity. Fig. S3a points to the fact that a single measurement of discharge capacity is not trustworthy and no meaningful conclusion can be deduced from comparing a limited number of observations. Actually, the C-x dependence is most probably essentially non-linear, as indicated by the discharge capacity values commonly referred to for NMCs with the compositions ranging from LiCoO_2_ to NMC9.5 0.25 0.25 (Fig. S3b). Thus, the linear fit should be considered as an approximation of a very short part of this non-linear curve in close vicinity of NMC811, without attempts to extrapolate it outside its validity area.

The discharge capacity decreases only slightly in the range of small antisite defect concentrations up to ∼4 %, but then drops down dramatically (Fig. 4c). Although the number of available capacity measurements complemented with the Rietveld-refined occupancy factor of Ni at the Li site is not large (104 entries only), and the calculated points do not strictly follow a smooth curve, the C-g_Ni_ dependence can roughly be approximated with the cubic equation:

(2) C = *a* - *b*g_Ni_^3^

where *a* = 195.9 ± 1.1, *b* = 0.0091 ± 0.0006. The relationship between antisite disorder and electrochemical capacity is quite sophisticated because the term “antisite” disorder involves point defects of different nature, which also differ in their spatial distribution between the surface and bulk parts of the NMC811 crystals. Ni at the Li site measured with the Rietveld refinement can appear due to true antisite disorder (i.e. Li^+^↔Ni^2+^ exchange between two crystallographic sites):

(3) $${\text{Ni}}_{\text{Ni}}^{\times }+{\text{Li}}_{\text{Li}}^{\times }\to {\text{Li}}_{\text{Ni}}^{\prime }+{\text{Ni}}_{\text{Li}}^{•}$$

or due to partial reduction of Ni^3+^ in its native site ${\text{Ni}}_{\text{Ni}}^{•}$ followed by migration to the Li site ${\text{Ni}}_{\text{Li}}^{•}$ with releasing oxygen that corresponds to gradual transition from the layered Li(Ni.Mn,Co)O_2_ structure to a rock-salt-type one:

(4) $${V_{\text{Li}}^{\prime }+\text{Ni}}_{\text{Ni}}^{•}+O_{O}^{\times }\to {\text{Ni}}_{\text{Li}}^{•}+V_{\text{Ni}}^{\prime \prime \prime }+V_{O}^{••}+1/2O_{2}$$

At small g_Ni_ of 3–4% the disorder follows equation (3) being confined to a few nanometers surface layer [25]. Although antisite disorder can impede Li ion transport [26], noticeable capacity deterioration apparently does not happen at these low defect concentrations that might be attributed to a discontinuous nature of the defective surface layer observed with quantitative transmission electron microscopy [25]. Thus, if the defect concentration is below the percolation threshold (estimated from Fig. 4c at g_Ni_ ≈ 4 %), the surface layer can still maintain transparency for Li-ion diffusion. As soon as oxygen loss is triggered (for instance, by rising temperature of the thermal treatment) [18,27], g_Ni_ may increase substantially following equation (4) causing severe capacity loss due to decreasing the Li content and driving the structure towards rock-salt NiO [17]. One can speculate (however, without straightforward proof) that the kink at g_Ni_ ≈ 5–6% in the plot in Fig. 4d might be related to switching the defect formation scheme from equation (3) to equation (4).

**Fig. 5:**
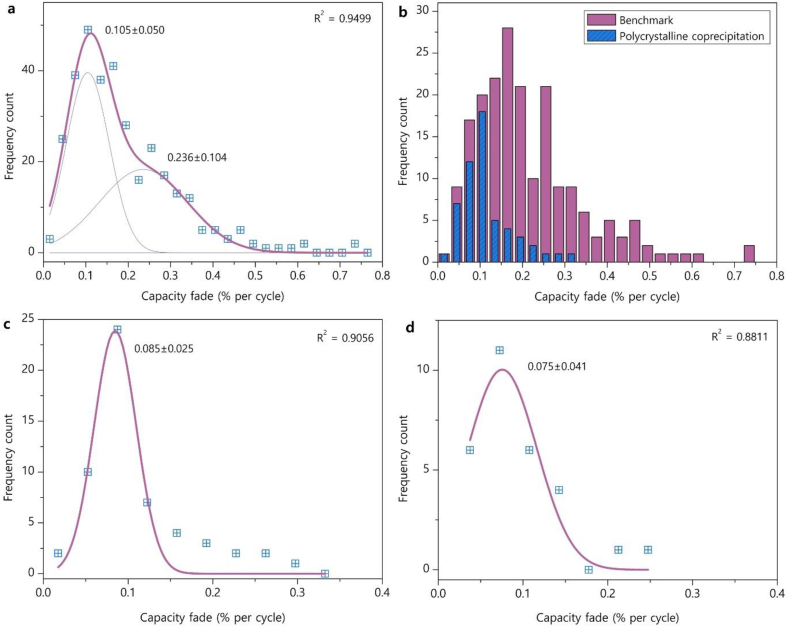
Distribution histogram of capacity fade (% per cycle, *E*_U_ = 4.3 V and C-rate of 1C) (a). The distribution is fitted with two Gaussian peaks (shown as faint lines). Distribution histograms of capacity fade for the “benchmark” measurements compared to the histogram of the “synthesis” measurements on polycrystalline samples obtained through coprecipitation (b). Distribution histograms of capacity fade for the “polycrystalline” (c) and “single-crystal” (d) samples fitted with Gaussian peaks. The mean values and standard deviations are marked. Adjusted R^2^ coefficients are provided as a goodness of fit measures.

Expectably, the distribution of capacity fade at *E*_U_ = 4.3 V and CR = 1C is also asymmetric (Fig. 5a), but with positive skewness, in contrast to the distribution of the discharge capacity. The capacity fade histogram was also fitted with two Gaussian peaks: the narrow one at ${\bar{\text{CF}}}_{1}$ = 0.105 ± 0.050 % per cycle and the broad one at ${\bar{\text{CF}}}_{2}$ = 0.236 ± 0.104 % per cycle that corresponds to ∼190 and ∼85 charge/discharge cycles at full depth till reaching the capacity retention of 80 %. The heavy positive skewness and broad peak of poor capacity retention originate mainly from the NMC811 samples used as “benchmark” (Fig. 5b), whereas the polycrystalline materials prepared with the most elaborated coprecipitation method demonstrate much narrower distribution peaked at the capacity fade of ∼0.1 % per cycle (Fig. 5b). As discussed above, this drastic difference evidences either quality deterioration of the samples used as “benchmark” or biasing when benchmarks of low performance have been selected for more remarkable demonstration of improvements. In order to eliminate the negative influence of these “benchmark” measurements, only the “synthesis” measurements were treated for “polycrystalline” and “single-crystal” NMC811 separately (Fig. 5c and d), as the “single-crystal” materials are reported to demonstrate superior capacity retention [[28], [29], [30]]. Indeed, the “single-crystal” materials show lower capacity fade (0.075 ± 0.041 % per cycle) than the “polycrystalline” ones (0.085 ± 0.025 % per cycle), but the difference is not truly remarkable, as reflected by ∼588-172 and 333-182 cycles, respectively, till 80 % capacity retention. One should stress that these values must be treated with care, keeping in mind that they belong to half-cells with Li metal anodes, whereas the capacity retentions of 80–84 % for 1000 cycles have been reported for single-crystal NMC811 in full Li-ion cells with graphite or Li_4_Ti_5_O_12_ anodes [31,32].

Asymmetric nature of the capacity fade distribution results in large positive offsets even for the median values thus preventing evaluation of the absolute capacity fade at different upper potential thresholds E_U_. Nevertheless, for the ratio of the median capacity fade at certain E_U_ to that at E_U_ = 4.3 V vs Li/Li^+^ such dependence can be tracked (Fig. S4). The relative CF(E_U_)/CF(4.3 V) values at CR = 1C behave almost linearly on E_U_ with the slope corresponding to doubling of capacity fade per each ∼0.35 V of the upper limit of the cycling potential window. This renders high upper voltage as an important factor diminishing the cycling stability of NMC811.

**Fig. 6:**
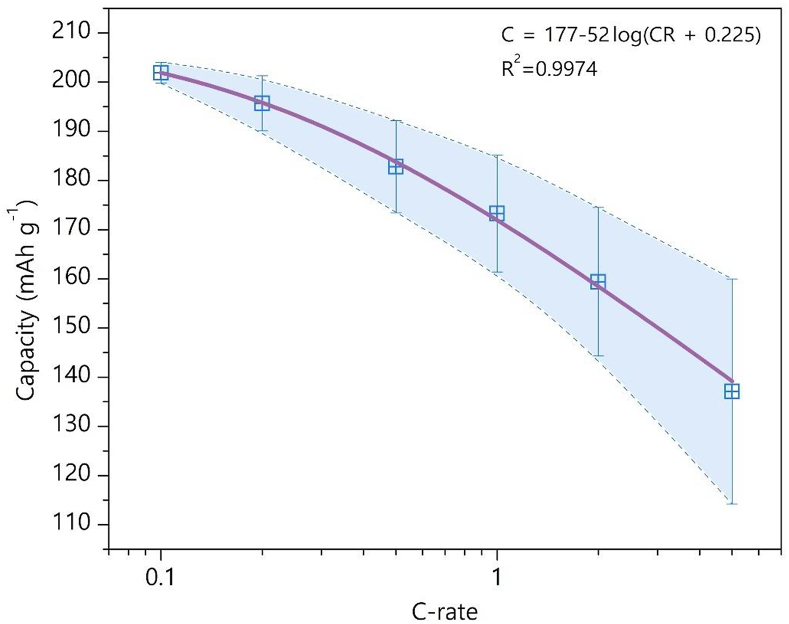
Discharge capacity vs C-rate at *E*_U_ = 4.3 V vs Li/Li^+^. C-rate scale is logarithmic. Adjusted R^2^ coefficient is provided as a goodness of fit measures.

Finally, the rate capability of NMC811 at E_U_ = 4.3 V vs Li/Li^+^ was quantified by analyzing histograms of discharge capacity distributions at the 0.2C, 0.5C, 1C, 2C and 5C current rates (Fig. S5). In contrast to the first discharge capacity histogram at CR = 0.1C (Fig. 2a), these histograms do not appear very asymmetric and can be fitted with a single Gaussian contribution. This difference is the shape of histograms is difficult to explain, but perhaps it might be attributed to that the rate capability was usually measured after the cell passed through few formation cycles. Even brief visual comparison of the histograms in Fig. S5 reveals that the distributions become broader with increasing the C-rate indicating steadily increasing capacity variance. This might be expected, as various factors impeding the Li-ion transport (crystal size, crystallite packing in agglomerates, porosity, concentration of antisite defects, composition and thickness of the electrode) demonstrate their negative impact on the rate capability more pronouncedly at higher CRs. The discharge capacity vs CR dependence is provided in Fig. 6 along with the standard deviations. The monotonic dependence can be fitted with a logarithmic function:

(5) C = *a* – *b∙*log(CR + *c*)

where *a* = 176.5 ± 1.7, *b* = 52.0 ± 4.0, *c* = 0.225 ± 0.051. This equation provides the discharge capacity limit of 210.7 mAh/g for NMC811 at infinitely small CR.

## 4 Conclusions

Data analysis from >500 publications comprising >950 entries on the electrochemical properties of the NMC811 cathode material allowed us to calculate quantitative measures of the key functional properties of NMC811, such as first cycle discharge capacity, first cycle Coulombic efficiency, capacity retention and rate capability and their links to the cycling potential limits, chemical composition and antisite disorder. The values listed in Table 1 provide the checkpoint for benchmarking the research targeting further optimization of the electrochemical performance of the NMC811 cathode materials and synthesis techniques. We verified the literature-retrieved data by the measurements of the same set of parameters on a single NMC811 specimen without any chemical modification (doping, coating etc.), which we synthesized via a standard hydroxide co-precipitation method (see Supporting Information). This verification is necessary as the statistical analysis of the published data does not guarantee that all the parameters from Table 1 could be realized simultaneously. The experimentally-determined electrochemical parameters correspond well the literature-derived data in Table 1, confirming their coherence.

**Table 1:** Main electrochemical parameters of the NMC811 cathode materials tested in half-cells with Li metal counter electrode.

Parameter	Literature-derived value	Experimental value (this work)
First cycle discharge capacity (E_U_ = 4.3 V, E_L_ = 2.5–3.0 V, CR = 0.1C), mAh/g	201.9 ± 2.1	200
First cycle Coulombic efficiency (E_U_ = 4.3 V, E_L_ = 2.5–3.0 V, CR = 0.1C), %	88.7 ± 2.6	90
First cycle discharge capacity (E_L_ = 2.5–3.0 V, CR = 0.1C) at:		
E_U_ = 4.2 V, mAh/g	179	180
E_U_ = 4.4 V, mAh/g	204	205
E_U_ = 4.5 V, mAh/g	209	210
E_U_ = 4.6 V, mAh/g	213	214
E_U_ = 4.7 V, mAh/g	217	217
E_U_ = 4.8 V, mAh/g	219	221
Capacity fade for polycrystalline materials (E_U_ = 4.3 V, E_L_ = 2.5–3.0 V, CR = 1C), % per cycle	0.085 ± 0.025	0.091–0.112
Capacity fade for single crystal materials (E_U_ = 4.3 V, E_L_ = 2.5–3.0 V, CR = 1C), % per cycle	0.075 ± 0.041	n/a
Discharge capacity (E_U_ = 4.3 V, E_L_ = 2.5–3.0 V) at:		
CR = 0.2C, mAh/g	195.6 ± 5.6	192
CR = 0.5C, mAh/g	182.8 ± 9.4	179
CR = 1C, mAh/g	173.3 ± 11.9	165
CR = 2C, mAh/g	159.4 ± 15.0	157
CR = 5C, mAh/g	137.1 ± 22.9	132

Notations: E_U_ – upper potential limit, E_L_ – lower potential limit, CR – current rate (1C = 200 mA/g). Voltage is given vs the Li/Li^+^ potential.

To sum up, the obtained set of quantitative estimates eliminates the necessity of picking up random references from hundreds of publications available to date. Moreover, the data from Table 1 may serve as the reference points for testing predictive machine-learning methods, whereas equations (1), (2), (5) call for rationalizing them using *ab initio* computational approaches.

Finally, large variance in the reported electrochemical parameters apparently calls for introducing commonly accepted protocols for testing the active electrode materials, through a consensus in the battery community, in a way as it was done in the field of photovoltaics [33]. For the case of NMC811, the recommended galvanostatic cycling regimes for testing and benchmarking can be captured from Table 1. However, besides the testing regimes, the assembling protocols for two-electrode coin cells with Li metal anode must be standardized that is beyond the scope of this manuscript. Some analysis of possible pitfalls, practical advices and hints can be found in few recent publications [[34], [35], [36], [37], [38]]. Developing a standardized commonly accepted methodology is crucially important for monitoring the progress in high-nickel cathodes which nowadays evolve along numerous directions, such as complex chemical doping, creating composite and/or chemical gradient structures, tuning the crystallite shapes and alignment, modifying grain boundaries, designing advanced single crystal morphologies and combinations whereof.

## Data availability statement

The raw numerical data, extracted from literature, are provided in Supporting Information along with Digital Object Identifiers (DOI) of the source publications.

## CRediT authorship contribution statement

**Aleksandra A. Savina:** Data curation, Investigation, Validation, Writing – review & editing. **Artem M. Abakumov:** Conceptualization, Data curation, Formal analysis, Funding acquisition, Methodology, Supervision, Writing – original draft.

## Declaration of competing interest

The authors declare that they have no known competing financial interests or personal relationships that could have appeared to influence the work reported in this paper.
