# Supplementary material for: Benchmarking the electrochemical parameters of the LiNi0.8Mn0.1Co0.1O2 positive electrode material for Li-ion batteries
Source: Heliyon. 2023 Nov 1;9(12):e21881. doi: 10.1016/j.heliyon.2023.e21881 (PMC10709181; doi:10.1016/j.heliyon.2023.e21881)
Supplement: Multimedia component 1 [file mmc1.pdf]

The raw numerical data extracted from literature and Digital Object Identifiers (DOI) of the source publications.

Composiition	First charge/discharge cycle at room temperature				Capacity retention					Rate capability at 4.3V upper voltage, room temperature				Ni content in Li site, %	Cathode composition, weight %			DOI or web reference	Tags
	First discharge capacity, mAh/g	Voltage range, V	Initial Coulombic efficiency, %	C-rate, 1C = 200 mAh/g	C-rate	Upper voltage, V	# of cycles	Capacity retention, %	Capacity fade, %/cycle	Capacity at 0.5C, mAh/g	Capacity at 1C, mAh/g	Capacity at 2C, mAh/g	Capacity at 5C, mAh/g		Active material	Conductive additive	Binder		
LiNi0.8Mn0.1Co0.1O2	200	2.7-4.3	89	0.1	0.5	4.3	100	94	0.060						90	5.5	4.5	10.1039/C8TA10438G	polycrystalline, synthesis, lab-made
LiNi0.8Mn0.1Co0.1O2	207	2.7-4.4			0.5	4.4	100	93	0.070						90	5.5	4.5		polycrystalline, synthesis, lab-made
LiNi0.8Mn0.1Co0.1O2	212	2.7-4.5	92		0.5	4.5	100	91	0.090						90	5.5	4.5		polycrystalline, synthesis, lab-made
Li1.02(Ni0.8Mn0.1Co0.1)0.98O1	166	2.7-4.3		0.1										7.9				10.1002/cssc.202001915	polycrystalline, synthesis, lab-made
Li1.02(Ni0.8Mn0.1Co0.1)0.98O2	198	2.7-4.3		0.1										7.6					polycrystalline, commercial
LiNi0.8Mn0.1Co0.1O2	175	2.7-4.2		0.1											80	10	10	10.1039/c8cc10017a	polycrystalline, commercial
LiNi0.8Mn0.1Co0.1O2	192	2.7-4.3		0.1											80	10	10		polycrystalline, commercial
LiNi0.8Mn0.1Co0.1O2	200	2.7-4.4		0.1											80	10	10		polycrystalline, commercial
LiNi0.8Mn0.1Co0.1O2	220	2.7-4.5	87	0.1	0.1	4.5	100	23	0.770						80	10	10		polycrystalline, commercial
LiNi0.8Mn0.1Co0.1O2	198	2.7-4.6	76	0.1	0.1	4.6	100	65	0.350					3.68	80	10	10	10.3390/coatings9020092	polycrystalline, benchmark, lab-made
LiNi0.8Mn0.1Co0.1O2	174	2.8-4.3	87	0.2	1	4.3	200	72	0.140	153	138	125			80	10	10	10.1039/D2TA01186G	polycrystalline, commercial
LiNi0.8Mn0.1Co0.1O2	198	2.8-4.3	86	0.2	1	4.3	200	80	0.100	173	163	152			80	10	10		single crystal, commercial
LiNi0.8Mn0.1Co0.1O2	205	2.8-4.3	90	0.1	1	4.3	100	54	0.460						80	10	10	10.1149/1945-7111/ac2021	polycrystalline, benchmark, commercial
LiNi0.8Mn0.1Co0.1O2	202	3.0-4.3	88	0.09	0.09	4.3	100	99	0.010						90	5	5	10.3389/fmats.2019.00309	polycrystalline, benchmark, lab-made
LiNi0.8Mn0.1Co0.1O2	179	2.8-4.2		0.33	0.33	4.2	379	80	0.053						95	2	3	10.3390/batteries8080079	polycrystalline, benchmark, commercial
LiNi0.8Mn0.1Co0.1O2	195	2.5-4.4	98	0.1											80	10	10	10.1038/s41563-022-01461-5	polycrystalline, synthesis, lab-made
LiNi0.8Mn0.1Co0.1O2	203	2.0-4.5	80	0.14	0.14	4.5	200	20	0.400						80	10	10	10.1016/j.nanoen.2020.105021	polycrystalline, synthesis, lab-made
LiNi0.83Mn0.06Co0.11O2	206	3.0-4.3	90	0.1	1	4.3	200	88	0.060	196	187	174	138	4.6	90	5	5	10.1021/jacs.2c03549	single crystal, synthesis, lab-made
LiNi0.76Mn0.14Co0.1O2	185	2.7-4.2	86	0.1	0.33	4.2	200	87	0.065					1.4	96	2	2	10.1126/science.abc3167	polycrystalline, synthesis, lab-made
LiNi0.76Mn0.14Co0.1O2	194	2.7-4.3	86	0.1	0.33	4.3	200	82	0.090					1.4	96	2	2		polycrystalline, synthesis, lab-made
LiNi0.76Mn0.14Co0.1O2	203	2.7-4.4	86	0.1	0.33	4.4	200	72	0.140					1.4	96	2	2		polycrystalline, synthesis, lab-made
LiNi0.8Mn0.1Co0.1O2	204	2.7-4.3	86	0.1	0.2	4.3	200	88	0.060						80	10	10	10.1016/j.ceramint.2019.12.204	polycrystalline, synthesis, lab-made
LiNi0.8Mn0.1Co0.1O2	201	2.7-4.3	88	0.1	1	4.3	200	81	0.095	196	183	149	105		80	10	10	10.1002/ente.201901437	polycrystalline, benchmark, lab-made
LiNi0.8Mn0.1Co0.1O2	214	2.7-4.3	95	0.1	0.5	4.3	100	96	0.040						90	5.5	4.5	10.1021/acsenergylett.1c01089	polycrystalline, synthesis, lab-made
LiNi0.8Mn0.1Co0.1O2	199	2.7-4.3	89	0.1	0.5	4.3	100	85	0.150						90	5.5	4.5		single crystal, synthesis, lab-made
LiNi0.78Mn0.11Co0.11O2	200	3.0-4.3		0.1	1	4.3	100	90	0.100	183	179	165	141		90	5	5	10.1002/smll.202105833	polycrystalline, synthesis, lab-made
					6	4.3	100	81	0.190						90	5	5		
LiNi0.79Mn0.11Co0.10O2	200	3.0-4.3		0.1	1	4.3	100	91	0.090	183	179	170	160		90	5	5		single crystal, synthesis, lab-made
					6	4.3	100	84	0.160						90	5	5		
LiNi0.77Mn0.12Co0.11O2	200	3.0-4.3		0.1	1	4.3	100	94	0.060	188	185	178	172		90	5	5		single crystal, synthesis, lab-made
					6	4.3	100	94	0.060						90	5	5		
LiNi0.8Mn0.1Co0.1O2	205	3.0-4.3	91	0.1	1	4.3	100	89	0.110						96	2	2	10.1002/adma.202003040	single crystal, commercial
LiNi0.8Mn0.1Co0.1O2	226	3.0-4.4	82	0.08C	0.8	4.4	200	74	0.130						80	10	10	10.1016/j.jpowsour.2020.228207	polycrystalline, synthesis, lab-made
					3.2	4.4	200	79	0.105						80	10	10		
LiNi0.8Mn0.1Co0.1O2	216	3.0-4.4	85	0.08C	0.8	4.4	200	82	0.090						80	10	10		single crystal, synthesis, lab-made
					3.2	4.4	200	85	0.075						80	10	10		
LiNi0.8Mn0.1Co0.1O2	201	2.8-4.3	80	0.1	1	4.3	300	91	0.030	185	177	174	160	3.76	80	10	10	10.1039/c8qm00326b	polycrystalline, synthesis, lab-made
LiNi0.8Mn0.1Co0.1O2	216	2.0-4.5	88	0.13	0.1	4.5	150	55	0.300						80	10	10	10.1016/j.nanoen.2018.09.066	polycrystalline, synthesis, lab-made
LiNi0.799Mn0.10Co0.101O2	188	2.8-4.3	82	0.2	0.2	17	100	85	0.150	179	169	151	124	6.78	80	10	10	10.1021/acsami.9b12113	polycrystalline, synthesis, lab-made
LiNi0.80Mn0.102Co0.098O2	193	2.8-4.3	84	0.2	0.2	4.3	100	96	0.040	192	187	175	166	3.44	80	10	10		polycrystalline, synthesis, lab-made
LiNi0.83Mn0.06Co0.11O2	200	2.75-4.4	89	1	1	4.4	150	85	0.100						89	5	3.5	10.1016/j.nanoen.2020.104450	polycrystalline, synthesis, lab-made
LiNi0.83Mn0.06Co0.11O2	200	2.75-4.4	89	1	1	4.4	150	68	0.213						89	5	3.5		single crystal, synthesis, lab-made
LiNi0.85Mn0.05Co0.10O2	185	2.7-4.3	84	0.1	0.5	4.3	100	89	0.110	165	162	150	141		80	10	10	10.1016/j.jpowsour.2018.08.006	polycrystalline, synthesis, lab-made
LiNi0.8Mn0.1Co0.1O2	185	2.8-4.2	85	0.1										3.1	94	3	3	10.1002/aenm.202103045	polycrystalline, synthesis, lab-made
LiNi0.8Mn0.1Co0.1O2	178	2.8-4.2		0.33	0.33	4.2	512	80	0.039					3.1	94	3	3		polycrystalline, synthesis, lab-made
LiNi0.8Mn0.1Co0.1O2	180	2.8-4.3	95	0.2	0.2	4.3	50	78	0.440	142	130	117			93	3	4	10.1002/aenm.201701682	polycrystalline, benchmark, commercial
LiNi0.8Mn0.1Co0.1O2	194	2.8-4.3		0.09	0.3	4.3	100	63	0.370		196	187			80	10	10	10.1021/acsenergylett.8b02302	polycrystalline, benchmark, commercial
LiNi0.794Mn0.094Co0.112O2	155	2.7-4.3	77	0.045	1	4.3	100	72	0.280						80	10	10	10.1016/j.ceramint.2019.10.195	polycrystalline, synthesis, lab-made
LiNi0.790Mn0.096Co0.114O2	170	2.7-4.3	81	0.045	1	4.3	100	71	0.290						80	10	10		polycrystalline, synthesis, lab-made
LiNi0.792Mn0.096Co0.112O2	195	2.7-4.3	80	0.045	1	4.3	100	71	0.290						80	10	10		polycrystalline, synthesis, lab-made
LiNi0.788Mn0.095Co0.117O2	224	2.7-4.3	83	0.045	1	4.3	100	71	0.290	173	152	127	87		80	10	10		polycrystalline, synthesis, lab-made
LiNi0.786Mn0.096Co0.118O2	218	2.7-4.3	84	0.045	1	4.3	100	67	0.330	170	152	127	87		80	10	10		polycrystalline, synthesis, lab-made
LiNi0.8Mn0.1Co0.1O2	153	2.7-4.3	72	0.1	1	4.3	100	67	0.330									10.20964/2020.03.63	polycrystalline, synthesis, lab-made
LiNi0.8Mn0.1Co0.1O2	231	2.7-4.3	79	0.1	1	4.3	100	79	0.210	175	156	139	108						polycrystalline, synthesis, lab-made
LiNi0.8Mn0.1Co0.1O2	216	2.7-4.3	83	0.1	1	4.3	100	80	0.200										polycrystalline, synthesis, lab-made
LiNi0.8Mn0.1Co0.1O2	202	2.7-4.3	82	0.1	1	4.3	100	68	0.320										polycrystalline, synthesis, lab-made
LiNi0.8Mn0.1Co0.1O2	168	3.0-4.35	79	0.2	0.2	4.35	20	80	1.000						80	10	10	10.20964/2016.12.19	polycrystalline, synthesis, lab-made
LiNi0.8Mn0.1Co0.1O2	173	3.0-4.35	91	0.2	0.2	4.35	20	90	0.500						80	10	10		polycrystalline, synthesis, lab-made
LiNi0.8Mn0.1Co0.1O2	195	3.0-4.35	98	0.2	0.2	4.35	20	84	0.800						80	10	10		polycrystalline, synthesis, lab-made
LiNi0.8Mn0.1Co0.1O2	172	3.0-4.35	91	0.2	0.2	4.35	20	85	0.750						80	10	10		polycrystalline, synthesis, lab-made
LiNi0.8Mn0.1Co0.1O2	169	3.0-4.35	97	0.2	0.2	4.35	20	94	0.300						80	10	10		polycrystalline, synthesis, lab-made
LiNi0.8Mn0.1Co0.1O2	154	3.0-4.35	75	0.2	0.2	4.35	20	89	0.550						80	10	10		polycrystalline, synthesis, lab-made
LiNi0.8Mn0.1Co0.1O2	159	3.0-4.35	76	0.2	0.2	4.35	20	90	0.500						80	10	10		polycrystalline, synthesis, lab-made
LiNi0.8Mn0.1Co0.1O2	163	3.0-4.35	69	0.2	0.2	4.35	20	80	1.000						80	10	10		polycrystalline, synthesis, lab-made
LiNi0.8Mn0.1Co0.1O2	170	3.0-4.35	73	0.2	0.2	4.35	20	99	0.050						80	10	10		polycrystalline, synthesis, lab-made
LiNi0.8Mn0.1Co0.1O2	207	3.0-4.35	75	0.2	0.2	4.35	20	95	0.250						80	10	10		polycrystalline, synthesis, lab-made
LiNi0.8Mn0.1Co0.1O2	178	3.0-4.35	79	0.2	0.2	4.35	20	92	0.400						80	10	10		polycrystalline, synthesis, lab-made
LiNi0.8Mn0.1Co0.1O2	161	3.0-4.35	76	0.2	0.2	4.35	20	93	0.350						80	10	10		polycrystalline, synthesis, lab-made
LiNi0.8Mn0.1Co0.1O2	198	2.7-4.5	72	0.07	0.7	4.5	50	71	0.580						80	10	10	10.1016/j.jallcom.2020.154571	polycrystalline, synthesis, lab-made
LiNi0.8Mn0.1Co0.1O2	196	2.5-4.3	89	0.09	0.45	4.3	50	58	0.840	177	164	152	115	4.02	85	8	7	10.1016/j.ssi.2013.07.023	polycrystalline, synthesis, lab-made
LiNi0.8Mn0.1Co0.1O2	200	2.5-4.3	91	0.09	0.45	4.3	150	65	0.233	177	167	153	138	2.44	85	8	7		polycrystalline, synthesis, lab-made
LiNi0.8Mn0.1Co0.1O2	185	2.5-4.3	85	0.09						177	167	154	142		85	8	7		polycrystalline, synthesis, lab-made
LiNi0.8Mn0.1Co0.1O2	192	2.5-4.3	88	0.09						172	159				85	8	7		polycrystalline, synthesis, lab-made
LiNi0.8Mn0.1Co0.1O2	199	2.5-4.3	89	0.09						180	169				85	8	7		polycrystalline, synthesis, lab-made

LiNi0.8Mn0.1Co0.1O2	200	2.5-4.3	91	0.09						177	167	153	138		85	8	7		polycrystalline, synthesis, lab-made
LiNi0.85Mn0.075Co0.075O2	193	2.8-4.3	97	0.1	0.1	4.3	80	95	0.063	193	188	182	174		80	10	10	10.1016/j.jechem.2017.11.025	polycrystalline, synthesis, lab-made
LiNi0.8Mn0.1Co0.1O2	205	2.8-4.3	81	0.09	1	4.3	100	96	0.040	189	179	165	133	2.1	80	10	10	10.1016/j.jpowsour.2016.12.095	polycrystalline, synthesis, lab-made
LiNi0.8Mn0.1Co0.1O2	198	3.0-4.3	86	0.1	0.2	4.3	60	94	0.100						80	6.8	13.2	10.1016/j.jpowsour.2005.11.083	polycrystalline, synthesis, lab-made
LiNi0.8Mn0.1Co0.1O2	184	2.7-4.3		0.09	0.1	4.3	30	92	0.267					5.5	80	10	10	10.1016/S1003-6326(10)60056-8	polycrystalline, synthesis, lab-made
LiNi0.85Mn0.075Co0.075O2	206	3.0-4.3	94	0.1	0.5	4.3	100	70	0.300						85	7.5	7.5	10.1016/j.jpowsour.2013.01.063	polycrystalline, synthesis, lab-made
LiNi0.8Mn0.1Co0.1O2	203	3.0-4.3	95	0.1	0.5	4.3	100	56	0.440						85	7.5	7.5		polycrystalline, synthesis, lab-made
LiNi0.81Mn0.08Co0.11O2	194	3.0-4.3	89	0.0925	0.925	4.3	100	90	0.100	172	164		140		80	10	10	10.1007/s11814-015-0154-3	polycrystalline, synthesis, lab-made
LiNi0.81Mn0.08Co0.11O2	211	3.0-4.3	94	0.0925											80	10	10		polycrystalline, synthesis, lab-made
LiNi0.81Mn0.08Co0.11O2	186	3.0-4.3	89	0.0925	0.925	4.3	100	85	0.150						80	10	10		polycrystalline, synthesis, lab-made
LiNi0.81Mn0.08Co0.11O2	186	3.0-4.3	87	0.0925	0.925	4.3	100	81	0.190						80	10	10		polycrystalline, synthesis, lab-made
LiNi0.81Mn0.08Co0.11O2	210	3.0-4.5	89	0.0925	0.925	4.5	100	44	0.560						80	10	10		polycrystalline, synthesis, lab-made
LiNi0.81Mn0.08Co0.11O2	218	3.0-4.7	89	0.0925	0.925	4.7	100	48	0.520						80	10	10		polycrystalline, synthesis, lab-made
LiNi0.8Mn0.1Co0.1O2	191	2.7-4.3	87	0.1	1	4.3	100	90	0.100	183	174	166	156		80	10	10	10.1016/j.ceramint.2015.08.159	polycrystalline, synthesis, lab-made
LiNi0.8Mn0.1Co0.1O2	185	2.75-4.3	84	0.3	0.3	4.3	100	95	0.050	183	178	173	165		90	4	6	10.1016/j.electacta.2016.07.040	polycrystalline, synthesis, lab-made
LiNi0.8Mn0.1Co0.1O2	193	2.8-4.3	88	0.2	1	4.3	100	87	0.130	186	180	175	167	1.01	90	5	5	10.1016/j.electacta.2019.135057	polycrystalline, synthesis, lab-made
LiNi0.8Mn0.1Co0.1O2	185	2.8-4.3	83	0.2	1	4.3	100	89	0.110	180	172	166	154	3.02	90	5	5		polycrystalline, synthesis, lab-made
LiNi0.8Mn0.1Co0.1O2	190	2.75-4.3		0.1	1	4.3	100	89	0.110	180	176	165	150	4.28	80	10	10	10.1016/j.electacta.2018.09.158	polycrystalline, benchmark, lab-made
					1	4.5	100	75	0.250					4.28	80	10	10		
LiNi0.8Mn0.1Co0.1O2	195	2.7-4.3		0.1	1	4.3	100	42	0.580	181	167	142	102	9.1	80	10	10	10.1016/j.cej.2020.126195	polycrystalline, benchmark, lab-made
					1	4.5	100	49	0.510					9.1	80	10	10		
LiNi0.8Mn0.1Co0.1O2	208	2.5-4.5	86	0.2	1	4.5	500	58	0.084						90	5	5	10.1021/acsami.9b14729	polycrystalline, benchmark, lab-made
LiNi0.8Mn0.1Co0.1O2	206	2.8-4.5	86	0.2	1	4.5	100	85	0.150					1.05	80	10	10	10.1016/j.jpowsour.2019.03.072	polycrystalline, benchmark, lab-made
LiNi0.84Mn0.06Co0.10O2	198	3.0-4.3	94	0.1	0.5	4.3	50	81	0.380	180	172	160	67		96	2	2	10.1038/s41598-019-54115-z	polycrystalline, benchmark, lab-made
LiNi0.8Mn0.1Co0.1O2	172	3.0-4.3		1	1	4.3	100	74	0.260						90	5	5	10.1016/j.ceramint.2018.12.196	polycrystalline, benchmark, lab-made
LiNi0.8Mn0.1Co0.1O2	211	3.0-4.3		1	1	4.3	50	87	0.260						90	5	5		polycrystalline, benchmark, lab-made
LiNi0.76Mn0.14Co0.10O2	210	2.7-4.5		0.1	0.33	4.5	250	81	0.076					5.33	80	10	10	10.1021/acsaem.9b02372	polycrystalline, benchmark, lab-made
LiNi0.8Mn0.1Co0.1O2	184	2.8-4.3		1	1	4.3	100	68	0.320	188	173	153	120		80	10	10	10.1016/j.jpowsour.2019.227337	polycrystalline, benchmark, lab-made
LiNi0.8Mn0.1Co0.1O2	205	2.8-4.5		1	1	4.5	100	64	0.360						80	10	10		polycrystalline, benchmark, lab-made
LiNi0.8Mn0.1Co0.1O2	205	2.8-4.3		0.2											80	10	10		polycrystalline, benchmark, lab-made
LiNi0.8Mn0.1Co0.1O2	190	2.8-4.3		0.1	0.33	4.3	100	74	0.260						80	10	10	10.14233/ajchem.2020.22543	polycrystalline, benchmark, lab-made
LiNi0.84Mn0.05Co0.11O2	201	3.0-4.3	89	0.1	0.5	4.3	80	72	0.350	186	173	153	25		96	2	2	10.1016/j.ijhydene.2020.04.292	polycrystalline, benchmark, lab-made
LiNi0.84Mn0.05Co0.11O2	225	3.0-4.5		0.1	0.5	4.5	80	63	0.463						96	2	2		polycrystalline, benchmark, lab-made
LiNi0.8Mn0.1Co0.1O2	202	2.5-4.5	81	0.08	0.08	4.5	80	62	0.475						80	10	10	10.1021/acs.inorgchem.7b01035	polycrystalline, benchmark, lab-made
LiNi0.8Mn0.1Co0.1O2	203	2.8-4.3	89	0.1	1	4.3	100	74	0.260	190	180	145		1.58	80	10	10	10.1016/S1003-6326(17)60132-8	polycrystalline, benchmark, lab-made
LiNi0.8Mn0.1Co0.1O2	166	2.8-4.3		0.1	1	4.3	100	70	0.300	162	152		134	4.9	80	10	10	10.1039/C7RA10053A	polycrystalline, benchmark, lab-made
LiNi0.8Mn0.1Co0.1O2	192	2.7-4.3	76	0.09	5	4.3	50	76	0.480		149		123	6.8	80	10	10	10.1016/j.jallcom.2012.01.004	polycrystalline, benchmark, lab-made
LiNi0.8Mn0.1Co0.1O2	200	2.8-4.3	83	0.1	1	4.3	300	67	0.110	174	156	145	136		80	10	10	10.1149/1945-7111/ab6977	polycrystalline, benchmark, lab-made
LiNi0.8Mn0.1Co0.1O2	198	2.75-4.35	78	0.09	0.18	4.35	100	85	0.150	182	175		143	3.05	90	4	6	10.1021/acs.jpcc.9b10526	polycrystalline, benchmark, lab-made
					0.9	4.35	100	71	0.290					3.05	90	4	6		
LiNi0.8Mn0.1Co0.1O2	213	3.0-4.3	88	0.085	0.425	4.3	100	77	0.230						80	10	10	10.1016/j.jallcom.2020.156210	polycrystalline, benchmark, lab-made
					0.85	4.3	100	79	0.210						80	10	10		
LiNi0.795Mn0.102Co0.103O2	201	3.0-4.3	93	0.2	0.5	4.3	70	84	0.229					4.1	80	10	10	10.1016/j.electacta.2009.01.048	polycrystalline, benchmark, lab-made
LiNi0.8Mn0.1Co0.1O2	182	3.0-4.3	83	0.1	0.1	4.3	100	64	0.360						80	10	10	10.1016/j.matlet.2020.127418	polycrystalline, benchmark, lab-made
LiNi0.8Mn0.1Co0.1O2	194	2.8-4.3	83	0.09	1	4.3	100	75	0.250	180	167	144	106	3.59	80	10	10	10.1007/s10008-019-04308-3	polycrystalline, benchmark, lab-made
LiNi0.8Mn0.1Co0.1O2	195	3.0-4.4		1	1	4.4	100	79	0.210						80	10	10	10.1016/j.electacta.2019.134889	polycrystalline, benchmark, lab-made
LiNi0.8Mn0.1Co0.1O2	212	2.7-4.6		0.18	0.9	4.6	100	72	0.280						80	10	10	10.1007/s11581-019-03430-6	polycrystalline, benchmark, lab-made
LiNi0.8Mn0.1Co0.1O2	194	2.7-4.3	86	0.1	1	4.3	100	85	0.150	160	138	109			80	10	10	10.1016/j.ceramint.2018.09.227	polycrystalline, benchmark, lab-made
LiNi0.8Mn0.1Co0.1O2	203	3.0-4.3	85	0.1	0.5	4.3	100	73	0.270	182	172	148			96	2	2	10.1016/j.jallcom.2019.03.308	polycrystalline, benchmark, lab-made
LiNi0.8Mn0.1Co0.1O2	195	2.7-4.3	86	0.1	1	4.3	100	80	0.200	190	180	167	146		80	10	10	10.1016/j.apsusc.2020.147245	polycrystalline, benchmark, lab-made
LiNi0.8Mn0.1Co0.1O2	196	2.7-4.3	82	0.1375	1.375	4.3	200	79	0.105	160	142	122	90		80	10	10	10.1016/j.jelechem.2019.113197	polycrystalline, benchmark, lab-made
LiNi0.8Mn0.1Co0.1O2	200	3.0-4.3		0.1	1	4.3	100	76	0.240		177	164	123		80	10	10	10.1016/j.jallcom.2018.06.020	polycrystalline, benchmark, lab-made
LiNi0.8Mn0.1Co0.1O2	201	2.7-4.5	99	0.09	0.18	4.5	400	63	0.093						80	10	10	10.1021/acsami.0c07931	polycrystalline, benchmark, lab-made
LiNi0.8Mn0.1Co0.1O2	200	3.0-4.4		0.1	0.5	4.4	100	91	0.090					2.2				10.1016/j.jechem.2019.02.006	polycrystalline, benchmark, lab-made
LiNi0.8Mn0.1Co0.1O2	211	2.8-4.3	78	0.1	1	4.3	100	69	0.310	170	150	127	97		80	10	10	10.1016/j.apsusc.2019.04.098	polycrystalline, benchmark, lab-made
					1	4.6	100	73	0.270						80	10	10		
LiNi0.8Mn0.1Co0.1O2	186	3.0-4.3	84	0.09	0.9	4.3	200	85	0.075	177	170	162	150		80	10	10	10.1016/j.electacta.2020.135871	polycrystalline, benchmark, lab-made
LiNi0.8Mn0.1Co0.1O2	203	2.8-4.5	86	0.1	1	4.5	50	85	0.300						85	10	5	10.1016/j.ssi.2019.05.005	polycrystalline, benchmark, lab-made
LiNi0.8Mn0.1Co0.1O2	187	2.7-4.3	80	0.085	1	4.3	170	37	0.371	154	142		113		80	13	7	10.1016/j.electacta.2018.02.049	polycrystalline, benchmark, commercial
LiNi0.8Mn0.1Co0.1O2	203	2.8-4.3	86	0.18	0.9	4.3	100	87	0.130	190	180	168	154		80	10	10	10.1016/j.ceramint.2018.05.024	polycrystalline, benchmark, lab-made
LiNi0.8Mn0.1Co0.1O2	203	2.75-4.5	86	0.09	0.9	4.5	100	51	0.490						80	10	10	10.1016/j.jallcom.2019.07.176	polycrystalline, benchmark, lab-made
LiNi0.85Mn0.10Co0.05O2	206	2.5-4.3	87	0.1	1	4.3	300	84	0.053	180	161	131	99	3.3	85	10	5	10.1149/1945-7111/ac24b8	polycrystalline, benchmark, lab-made
LiNi0.85Mn0.10Co0.05O2	198	2.5-4.3	83	0.2	1	4.3	300	84	0.053	182	154	125	94	1.93	85	10	5	10.1149/1945-7111/abebf9	polycrystalline, benchmark, lab-made
LiNi0.8Mn0.1Co0.1O2	235	2.8-4.5	87	0.1	1	4.3	200	66	0.170						80	10	10	10.1016/j.jallcom.2020.156467	polycrystalline, benchmark, lab-made
					1	4.5	200	52	0.240						80	10	10		
LiNi0.8Mn0.1Co0.1O2	189	3.0-4.3	80	0.14	0.28	4.3	100	77	0.230	179	169	160	152		80	10	10	10.1021/acssuschemeng.9b05539	polycrystalline, benchmark, commercial
					2.8	4.3	100	67	0.330						80	10	10		
LiNi0.8Mn0.1Co0.1O2	193	3.0-4.3	92	0.5	0.5	4.3	80	74	0.325	178	172	160	50		96	2	2	10.1038/s41598-020-67818-5	polycrystalline, benchmark, lab-made
LiNi0.8Mn0.1Co0.1O2	206	3.0-4.3		0.1	1	4.3	50	63	0.740	171	157	100			80	10	10	10.1016/j.apsusc.2019.06.242	polycrystalline, benchmark, commercial
LiNi0.8Mn0.1Co0.1O2	192	2.8-4.3	82	0.1	1	4.3	100	83	0.170	173	160	142	117		80	10	10	10.1016/j.jallcom.2020.156387	polycrystalline, benchmark, commercial
LiNi0.8Mn0.1Co0.1O2	200	2.8-4.3	90	0.1	1	4.3	100	65	0.350	189	173	152	95		90	5	5	10.1016/j.cej.2020.126813	polycrystalline, benchmark, commercial
LiNi0.8Mn0.1Co0.1O2	206	2.8-4.5	85	0.1	0.1	4.5	50	86	0.280						80	10	10	10.1021/acsami.7b08006	polycrystalline, benchmark, lab-made
					1	4.5	200	66	0.170						80	10	10		
LiNi0.8Mn0.1Co0.1O2	210	3.0-4.4	81	0.09	1	4.4	100	83	0.170						80	10	10	10.1007/s10008-020-04523-3	polycrystalline, benchmark, lab-made
LiNi0.8Mn0.1Co0.1O2	210	2.7-4.3	82	0.09	1	4.3	100	75	0.250	190	180		145	4.26	80	10	10	10.1002/adfm.201808825	polycrystalline, benchmark, lab-made
LiNi0.8Mn0.1Co0.1O2	200	2.8-4.3	94	0.1	1	4.3	90	88	0.133	185	172	158	140		80	10	10	10.1016/j.jallcom.2020.156763	polycrystalline, benchmark, lab-made
LiNi0.8Mn0.1Co0.1O2	199	2.5-4.3	81	0.2	0.5	4.5	200	51	0.245						80	10	10	10.1021/acsaem.0c01420	polycrystalline, benchmark, commercial
LiNi0.8Mn0.1Co0.1O2	220	2.5-4.5		0.1											80	10	10		polycrystalline, benchmark, commercial
LiNi0.8Mn0.1Co0.1O2	173	2.7-4.3	91	0.9	0.9	4.3	100	71	0.290						80	13	7	10.1016/j.jpowsour.2019.03.073	polycrystalline, benchmark, lab-made
LiNi0.8Mn0.1Co0.1O2	217	2.8-4.6	90	0.1	0.33	4.6	60	82	0.300						90	5	5	10.1021/acsami.9b09696	polycrystalline, benchmark, commercial
LiNi0.8Mn0.1Co0.1O2	215	3.0-4.4	82	0.1	1	4.4	200	80	0.100					5.57	80	10	10	10.1016/j.jallcom.2019.05.217	polycrystalline, benchmark, lab-made
					1	4.5	100	77	0.230					5.57	80	10	10		

LiNi0.8Mn0.1Co0.1O2	193	2.8-4.3	80	0.5	1	4.3	100	82	0.180	192	176	149	116		85	10	5	10.1016/j.jallcom.2018.09.281	polycrystalline, benchmark, lab-made
					0.5	4.5	100	84	0.160						85	10	5		
LiNi0.8Mn0.1Co0.1O2	200	2.7-4.3		0.1	1	4.3	200	48	0.260						80	10	10	10.1007/s11581-019-03316-7	polycrystalline, benchmark, lab-made
LiNi0.8Mn0.1Co0.1O2	190	2.8-4.3	81	0.1	1	4.3	170	81	0.112						80	10	10	10.1016/j.electacta.2016.06.110	polycrystalline, benchmark, lab-made
LiNi0.8Mn0.1Co0.1O2	200	3.0-4.3	90	0.1	1	4.3	200	53	0.235						96	2	2	10.1039/C8EE00155C	polycrystalline, benchmark, lab-made
LiNi0.8Mn0.1Co0.1O2	196	2.8-4.5	89	1	1	4.5	100	75	0.250						80	10	10	10.1016/j.apsusc.2019.144506	polycrystalline, benchmark, lab-made
LiNi0.8Mn0.1Co0.1O2	188	2.5-4.3	86	0.09	0.5	4.3	400	79	0.053	179	165	144	56		92	4	4	10.1021/acsami.9b02889	polycrystalline, benchmark, commercial
LiNi0.82Mn0.06Co0.12O2	199	3.0-4.3	89	0.095	0.95	4.3	50	93	0.140						95	2	3	10.1021/acsami.8b13766	polycrystalline, benchmark, commercial
LiNi0.8Mn0.1Co0.1O2	189	2.8-4.3	86	0.09	0.5	4.3	184	80	0.109	177	165	151	118	3.34	80	10	10	10.1016/j.cej.2020.128343	polycrystalline, benchmark, lab-made
LiNi0.82Mn0.09Co0.09O2	201	2.5-4.25	86	0.1	0.5	4.3	60	83	0.283					1.29	96	2	2	10.1002/ente.202000800	polycrystalline, benchmark, commercial
LiNi0.8Mn0.1Co0.1O2	214	3.0-4.5	84	0.1	1	4.3	1000	24	0.076					3.68	80	10	10	10.1016/j.jpowsour.2020.228745	polycrystalline, benchmark, lab-made
					1	4.5	380	48	0.137					3.68	80	10	10		
LiNi0.8Mn0.1Co0.1O2	179	2.75-4.5	87	0.1	0.2	4.5	50	93	0.140						80	10	10	10.1002/cssc.201802304	polycrystalline, benchmark, lab-made
					5	4.5	100	71	0.290						80	10	10		
LiNi0.8Mn0.1Co0.1O2	195	2.8-4.3	85	0.2	1	4.3	100	83	0.170	188	182	170	155		80	10	10	10.1016/j.jpowsour.2018.07.037	polycrystalline, benchmark, lab-made
LiNi0.8Mn0.1Co0.1O2	195	3.0-4.3	88	0.095	0.5	4.3	50	91	0.180	183	175	170			95	2	3	10.1016/j.electacta.2018.06.062	polycrystalline, benchmark, lab-made
LiNi0.82Mn0.06Co0.12O2	214	2.7-4.35	91	0.1	1	4.3	100	67	0.330									10.1016/j.jpowsour.2020.228235	polycrystalline, benchmark, commercial
LiNi0.8Mn0.1Co0.1O2	187	3.0-4.25	85	0.1	0.5	4.25	100	60	0.400	179	168	149	18		94	3	3	10.1021/acsami.0c10799	polycrystalline, benchmark, lab-made
LiNi0.8Mn0.1Co0.1O2	217	2.8-4.3	100	0.1	1	4.3	100	66	0.340	197	177	153	112	2.6	80	10	10	10.1021/acsami.9b04050	polycrystalline, benchmark, lab-made
LiNi0.8Mn0.1Co0.1O2	195	2.8-4.3	86	0.2	1	4.3	105	83	0.162	187	178	168	154		80	10	10	10.1021/acsami.8b02396	polycrystalline, benchmark, lab-made
LiNi0.8Mn0.1Co0.1O2	193	3.0-4.3	99	0.2	0.5	4.3	500	50	0.100	180	156	137	105	3.6	80	10	10	10.1021/acsami.9b12578	polycrystalline, benchmark
					0.5	4.5	500	35	0.130					3.6	80	10	10		
LiNi0.8Mn0.1Co0.1O2	174	3.0-4.3	77	0.2	0.5	4.3	150	88	0.080	171	160	144	118	2.56	80	10	10	10.1016/j.jpowsour.2019.03.014	polycrystalline, benchmark, lab-made
LiNi0.8Mn0.1Co0.1O2	207	2.7-4.3	88	0.1	1	4.3	100	59	0.410	189	180	170	147		80	10	10	10.1016/j.nanoen.2019.104043	polycrystalline, benchmark, commercial
LiNi0.8Mn0.1Co0.1O2	223	2.75-4.4	86	0.1	1	4.4	250	47	0.212						80	10	10	10.1039/D0TA08879J	polycrystalline, benchmark, commercial
LiNi0.8Mn0.1Co0.1O2	208	2.7-4.3	83	0.09	0.9	4.3	200	75	0.125	175	165		135	3.56	80	10	10	10.1016/j.electacta.2020.137120	polycrystalline, benchmark, lab-made
LiNi0.78Mn0.11Co0.11O2	189	3.0-4.3	86	0.09	0.9	4.3	50	97	0.060	178	171		153	3.1	93	4	3	10.1149/1945-7111/ac8a1b	polycrystalline, benchmark, lab-made
LiNi0.78Mn0.11Co0.11O2	188	3.0-4.3	86	0.09	0.9	4.3	50	97	0.060	178	171		153	5.1	93	4	3		polycrystalline, benchmark, lab-made
					0.9	4.5	100	80	0.200					5.1	93	4	3		
Li0.98Ni0.812Mn0.097Co0.091O	198	2.7-4.3	82	0.1	1	4.3	300	68	0.107	172	150	137	110	1.73	80	10	10	10.3390/batteries8070067	polycrystalline, synthesis, lab-made
Li1.08Ni0.804Mn0.096Co0.1O2	203	2.7-4.3	87	0.1	1	4.3	300	73	0.090	178	165	151	130	1.67	80	10	10		polycrystalline, synthesis, lab-made
Li0.93Ni0.815Mn0.091Co0.094O	200	2.7-4.3	84	0.1	1	4.3	300	64	0.120	174	154	145	120	1.83	80	10	10		polycrystalline, synthesis, lab-made
Li1.02Ni0.795Mn0.106Co0.099O	199	2.7-4.3	78	0.1	1	4.3	300	69	0.103	178	165	156	131	2.54	80	10	10		polycrystalline, synthesis, lab-made
LiNi0.822Mn0.095Co0.099O2	184	2.7-4.3		0.1	1	4.3	100	91	0.090	160	142			4.63	80	10	10	10.3390/nano10122381	polycrystalline, synthesis, lab-made
LiNi0.786Mn0.110Co0.121O2	181	2.7-4.3		0.1	1	4.3	100	73	0.270	129	101			5.95	80	10	10		polycrystalline, synthesis, lab-made
LiNi0.783Mn0.097Co0.106O2	160	2.7-4.3		0.1	1	4.3	100	85	0.150	128	109			6.34	80	10	10		polycrystalline, synthesis, lab-made
LiNi0.788Mn0.099Co0.103O2	156	2.7-4.3		0.1	1	4.3	93	69	0.333	123	95			6.92	80	10	10		polycrystalline, synthesis, lab-made
LiNi0.792Mn0.103Co0.099O2	199	2.7-4.3		0.1	1	4.3	100	90	0.100	179	164			2.54	80	10	10		polycrystalline, synthesis, lab-made
LiNi0.781Mn0.110Co0.109O2	193	2.7-4.3		0.1	1	4.3	100	81	0.190	174	157			2.12	80	10	10		polycrystalline, synthesis, lab-made
LiNi0.792Mn0.109Co0.106O2	186	2.7-4.3		0.1	1	4.3	100	97	0.030	168	156			3.05	80	10	10		polycrystalline, synthesis, lab-made
LiNi0.785Mn0.107Co0.108O2	181	2.7-4.3		0.1	1	4.3	100	91	0.090	154	135			4.43	80	10	10		polycrystalline, synthesis, lab-made
LiNi0.8Mn0.1Co0.1O2	203	3.0-4.5	75	0.1	1	4.5	100	63	0.370					3.26	80	10	10	10.1021/acsami.7b08802	polycrystalline, synthesis, lab-made
LiNi0.8Mn0.1Co0.1O2	185	3.0-4.3	86	0.0925	0.925	4.3	100	94	0.060	179	171		132		80	10	10	10.1149/2.1631712jes	polycrystalline, synthesis, lab-made
LiNi0.8Mn0.1Co0.1O2	210	3.0-4.5	88	0.0925	0.925	4.5	100	82	0.180						80	10	10		polycrystalline, synthesis, lab-made
LiNi0.8Mn0.1Co0.1O2	205	3.0-4.6		0.1	0.5	4.6	50	58	0.840						80	10	10	10.1016/j.jpowsour.2017.01.066	polycrystalline, benchmark, lab-made
					1	4.6	100	21	0.790						80	10	10		
LiNi0.8Mn0.1Co0.1O2	195	3.0-4.4		0.1	0.5	4.4	100	53	0.470						80	10	10		polycrystalline, benchmark, lab-made
LiNi0.8Mn0.1Co0.1O2	185	3.0-4.5		0.1	0.5	4.5	100	36	0.640						80	10	10		polycrystalline, benchmark, lab-made
LiNi0.8Mn0.1Co0.1O2	190	2.8-4.3	80	0.1	1	4.3	200	77	0.115					5.63	80	10	10	10.1016/j.electacta.2017.03.054	polycrystalline, benchmark, lab-made
LiNi0.8Mn0.1Co0.1O2	202	3.0-4.3	82	0.1	1	4.3	100	54	0.460	185	166	148	125		80	10	10	10.1016/j.jallcom.2017.02.224	polycrystalline, benchmark, lab-made
LiNi0.8Mn0.1Co0.1O2	200	2.8-4.3	83	0.1	1	4.3	100	83	0.170	184	181	162	140		80	10	10	10.1016/j.jelechem.2018.06.035	polycrystalline, benchmark, lab-made
LiNi0.8Mn0.1Co0.1O2	201	3.0-4.3	87	0.1	0.5	4.3	50	74	0.520	184	170	144			80	10	10	10.1016/j.cej.2019.122959	polycrystalline, benchmark, lab-made
LiNi0.8Mn0.1Co0.1O2	206	2.75-4.5	96	0.1	0.4	4.5	113	71	0.257									10.1021/acsnano.7b02561	polycrystalline, benchmark, commercial
LiNi0.8Mn0.1Co0.1O2	159	3.0-4.1	87	0.05	0.2	4.1	57	99	0.018					3.00	92	4	4	10.1149/2.1011507jes	polycrystalline, benchmark, commercial
LiNi0.8Mn0.1Co0.1O2	178	3.0-4.2	89	0.05	0.2	4.1	57	98	0.035					3.00	92	4	4		polycrystalline, benchmark, commercial
LiNi0.8Mn0.1Co0.1O2	195	3.0-4.3	90	0.05	0.2	4.1	57	95	0.088					3.00	92	4	4		polycrystalline, benchmark, commercial
LiNi0.8Mn0.1Co0.1O2	203	3.0-4.4	90	0.05	0.2	4.1	57	74	0.456					3.00	92	4	4		polycrystalline, benchmark, commercial
LiNi0.8Mn0.1Co0.1O2	203	2.7-4.5	81	0.2	0.2	4.5	100	64	0.360					3.62	80	10	10	10.1021/acsami.5b00788	polycrystalline, synthesis, lab-made
LiNi0.76Mn0.14o0.10O2	200	2.7-4.5	82	0.2	0.2	4.5	100	73	0.270					3.96	80	10	10		polycrystalline, synthesis, lab-made
Li 0.994Ni0.794Mn0.102Co0.108	217	2.7-4.6	81	0.2	0.2	4.6	100	56	0.440						80	10	10	10.1021/acsami.5b00645	polycrystalline, synthesis, lab-made
Li1.021Ni0.776Mn0.098Co0.105	208	2.7-4.6	78	0.2	0.2	4.6	100	60	0.400						80	10	10		polycrystalline, synthesis, lab-made
Li1.051Ni0.755Mn0.093Co0.101	195	2.7-4.6	73	0.2	0.2	4.6	100	77	0.230						80	10	10		polycrystalline, synthesis, lab-made
LiNi0.8Mn0.1Co0.1O2	214	2.8-4.6	78	0.2	0.2	4.6	100	78	0.220						80	10	10	10.1016/j.jtice.2020.09.001	polycrystalline, benchmark, lab-made
					0.5	4.6	100	67	0.330						80	10	10		
					1	4.6	100	67	0.330						80	10	10		
					2	4.6	100	52	0.480						80	10	10		
					5	4.6	100	40	0.600						80	10	10		
					10	4.6	100	43	0.570						80	10	10		
LiNi0.8Mn0.1Co0.1O2	206	2.8-4.3	86	0.1	1	4.3	300	70	0.100	186	177	168	158		80	10	10	10.1016/j.jallcom.2015.10.177	polycrystalline, benchmark, lab-made
					2	4.3	100	87	0.130						80	10	10		
					5	4.3	100	78	0.220						80	10	10		
					10	4.3	100	72	0.280						80	10	10		
LiNi0.8Mn0.1Co0.1O2	196	2.8-4.4	91	1	1	4.4	300	61	0.130						80	10	10		polycrystalline, benchmark, lab-made
LiNi0.8Mn0.1Co0.1O2	197	2.7-4.3		0.085	0.85	4.3	500	41	0.118	159	150		125		80	13	7	10.1016/j.jallcom.2018.06.364	polycrystalline, benchmark, lab-made
					4.25	4.3	100	54	0.460						80	13	7		
LiNi0.8Mn0.1Co0.1O2	203	2.8-4.3		0.09	1	4.3	100	83	0.170	188	181		152		80	10	10	10.1016/j.electacta.2020.135870	polycrystalline, benchmark, lab-made
LiNi0.8Mn0.1Co0.1O2 (SC)	203	2.8-4.3	88	0.0925	1	4.3	100	87	0.130	186	180	168	147		80	10	10	10.1016/j.electacta.2020.136075	polycrystalline, benchmark, commercial
LiNi0.8Mn0.1Co0.1O2	193	2.8-4.3		0.2	1	4.3	100	81	0.190	187	179		145		80	10	10	10.1016/j.apsusc.2019.03.116	polycrystalline, benchmark, lab-made
LiNi0.8Mn0.1Co0.1O2	200	2.8-4.4		0.2	1	4.4	100	65	0.350						80	10	10		polycrystalline, benchmark, lab-made
LiNi0.8Mn0.1Co0.1O2	212	2.8-4.5		0.2	1	4.5	100	58	0.420						80	10	10		polycrystalline, benchmark, lab-made
LiNi0.8Mn0.1Co0.1O2	210	2.8-4.5	90	0.3	3	4.5	100	51	0.490						80	10	10	10.1021/acsami.0c00643	polycrystalline, benchmark, commercial
LiNi0.8Mn0.1Co0.1O2	209	2.8-4.5	80	0.1	1	4.5	200	70	0.150						80	10	10	10.1002/cssc.201702451	polycrystalline, benchmark, lab-made

LiNi0.8Mn0.1Co0.1O2	195	2.8-4.3	79	0.09	1	4.3	150	65	0.233	165	146		118	4.4	80	10	10	10.1016/j.electacta.2019.135086	polycrystalline, benchmark, lab-made
					5	4.3	50	83	0.340					4.4	80	10	10		
LiNi0.8Mn0.1Co0.1O2	191	2.8-4.3	84	0.1	1	4.3	200	82	0.090	185	174	160	141	2.9	80	10	10	10.1016/j.electacta.2018.01.119	polycrystalline, benchmark, lab-made
LiNi0.8Mn0.1Co0.1O2	193	2.75-4.3	79	0.2	0.2	4.3	100	90	0.100					4.71	80	10	10	10.1021/acsami.9b12595	polycrystalline, benchmark, lab-made
					1	4.3	200	92	0.040					4.71	80	10	10		
LiNi0.8Mn0.1Co0.1O2	215	2.75-4.5	84	0.2	0.2	4.5	100	85	0.150					4.71	80	10	10		polycrystalline, benchmark, lab-made
					1	4.5	200	64	0.180					4.71	80	10	10		
LiNi0.8Mn0.1Co0.1O2	176	3.0-4.45	82	0.1											95	2.5	2.5	10.1021/am300386j	single crystal, synthesis, lab-made
LiNi0.8Mn0.1Co0.1O2	200	2.7-4.3	79	0.1	1	4.3	100	87	0.130	182	170	156	131	2.4	89	5	6	10.1039/D2YA00211F	polycrystalline, synthesis, lab-made
					1	4.3	200	76	0.120					2.4	89	5	6		
					1	4.3	300	70	0.100					2.4	89	5	6		
LiNi0.8Mn0.1Co0.1O2	200	2.7-4.3	76	0.1	1	4.3	100	95	0.050	187	182	175	162	1.2	89	5	6		single crystal, synthesis, lab-made
					1	4.3	200	88	0.060					1.2	89	5	6		
					1	4.3	300	81	0.063					1.2	89	5	6		
LiNi0.8Mn0.1Co0.1O2	200	3.0-4.3	83	0.05											90	5	5	10.1021/acs.chemmater.9b00140	polycrystalline, commercial
LiNi0.8Mn0.1Co0.1O2	208	3.0-4.4	87	0.05											91.5	4.4	4.1	10.1149/2.0401802jes	polycrystalline, commercial
LiNi0.8Mn0.1Co0.1O2	187	2.5-4.3	89	0.1						152	142	131	110	2.3	80	10	10	10.1021/acs.jpcc.0c00720	polycrystalline, commercial
LiNi0.8Mn0.1Co0.1O2	195	3.0-4.4	93	0.1	0.1	4.4	100	85	0.150					1.6	80	10	10	10.1002/admi.202200035	polycrystalline, benchmark, commercial
					1	4.4	400	55	0.113					1.6	80	10	10		
LiNi0.8Mn0.1Co0.1O2	209	3.0-4.3		0.05	0.5	4.3	200	64	0.180					1.5	88	6	6	10.1021/acs.chemmater.2c02580	polycrystalline, benchmark, commercial
LiNi0.8Mn0.1Co0.1O2	200	3.0-4.5	88	0.1	1	4.5	80	88	0.150					1.8	94	3	3	10.1149/1945-7111/aca360	coprecipitation, benchmark for W doping
LiNi0.8Mn0.1Co0.1O2	190	2.5-4.25		0.08	2.4	4.25	100	42	0.580	166	140	120	94		80	10	10	10.1080/10667857.2021.2009095	polycrystalline, benchmark, commercial
LiNi0.8Mn0.1Co0.1O2	205	2.8-4.3	91	0.1	1	4.3	100	53	0.470						80	10	10	10.1021/acsami.1c16373	polycrystalline, benchmark, commercial
LiNi0.8Mn0.1Co0.1O2	202	3.0-4.4	90	0.1	0.1	4.4	200	40	0.300						90	5	5	10.1016/j.jpowsour.2019.227395	polycrystalline, benchmark, commercial
LiNi0.8Mn0.1Co0.1O2	216	2.8-4.6	89	0.1	0.33	4.4	250	83	0.068						90	5	5	10.1021/acsenergylett.1c00190	polycrystalline, benchmark, commercial
LiNi0.8Mn0.1Co0.1O2	173	3.0-4.3	82	0.1	1	4.3	100	59	0.410						83	10	7	10.1016/j.jmrt.2023.01.102	polycrystalline, benchmark, lab-made
LiNi0.8Mn0.1Co0.1O2	167	2.8-4.3		0.1	0.1	4.3	100	96	0.040						92.6	3.7	3.7	10.1002/smll.202200627	polycrystalline, commercial
LiNi0.8Mn0.1Co0.1O2	194	2.8-4.6		0.1	0.1	4.4	100	73	0.270						92.6	3.7	3.7		polycrystalline, commercial
LiNi0.8Mn0.1Co0.1O2	203	2.5-4.5		0.1	2	4.5	88	69	0.352						80	10	10	10.3390/ma15062146	polycrystalline, benchmark, commercial
LiNi0.8Mn0.1Co0.1O2	200	2.7-4.6	70	0.5	0.5	4.6	300	78	0.073					3.02	80	10	10	10.1039/C9TA07147D	polycrystalline, benchmark, lab-made
LiNi0.8Mn0.1Co0.1O2	204	2.7-4.6	80	0.1	0.1	4.6	50	89	0.220					3.02	80	10	10		polycrystalline, benchmark, lab-made
					1	4.6	250	70	0.120					3.02	80	10	10		
					5	4.6	500	76	0.048					3.02	80	10	10		
					1	4.6	250	67	0.132					3.02	80	10	10		
LiNi0.8Mn0.1Co0.1O2	176	3.0-4.3		0.09	0.5	4.3	200	40	0.300	128	95	38			95.2	2.4	2.4	10.1016/j.jpowsour.2022.232324	polycrystalline, benchmark, commercial
LiNi0.8Mn0.1Co0.1O2	205	3.0-4.5		0.1	0.5	4.3	250	59	0.164						80	10	10	10.1016/j.coco.2022.101356	polycrystalline, benchmark
					0.5	4.5	200	4.3	0.479						80	10	10		
LiNi0.8Mn0.1Co0.1O2	193	2.8-4.3	88	0.1	1	4.3	484	80	0.041					2.23	80	10	10	10.1016/j.ensm.2019.08.006	polycrystalline, benchmark, lab-made
LiNi0.8Mn0.1Co0.1O2	194	2.8-4.4	84	0.1	0.5	4.4	60	81	0.317					4.6	90	5	5	10.1149/1945-7111/ac0020	polycrystalline, benchmark, lab-made
LiNi0.8Mn0.1Co0.1O2	185	2.5-4.3	80	0.1	0.5	4.3	100	93	0.070	158	147		99		80	10	10	10.1016/j.jallcom.2021.158806	polycrystalline, benchmark, lab-made
LiNi0.8Mn0.1Co0.1O2	202	3.0-4.8	88	0.09	0.09	4.8	100	66	0.340						80	10	10	10.1016/j.mtsust.2022.100236	polycrystalline, benchmark, lab-made
LiNi0.8Mn0.1Co0.1O2	200	2.8-4.3	81	0.1	0.33	4.25	300	72	0.093	179	157	141	120	3.4	80	10	10	10.1016/j.xcrp.2022.100767	polycrystalline, benchmark, lab-made
					1	4.25	300	62	0.127					3.4	80	10	10		
LiNi0.8Mn0.1Co0.1O2	200	3.0-4.3		0.1	1	4.3	500	12	0.176	181	172	150	75		86	7	7	10.1016/j.jechem.2022.02.015	polycrystalline, benchmark, lab-made
LiNi0.8Mn0.1Co0.1O2	212	3.0-4.5		0.1	1	4.5	200	50	0.250						86	7	7		polycrystalline, benchmark, lab-made
LiNi0.8Mn0.1Co0.1O2	225	3.0-4.7		0.1	1	4.7	200	47	0.265						86	7	7		polycrystalline, benchmark, lab-made
LiNi0.8Mn0.1Co0.1O2	200	2.7-4.5	83	0.1	0.33	4.5	200	53	0.235						86	7	7		polycrystalline, benchmark, commercial
LiNi0.8Mn0.1Co0.1O2	203	3.0-4.3	89	0.05	0.2	4.3	100	85	0.150					1.5	92	4	4	10.1149/1945-7111/abb350	single cystal, commercial
LiNi0.8Mn0.1Co0.1O2	212	3.0-4.3	93	0.05	0.2	4.3	100	90	0.100						92	4	4		polycrystalline, commercial
LiNi0.8Mn0.1Co0.1O2	171	2.8-4.2		0.1	0.5	4.2	100	92	0.080						92	4	4	10.1007/s00706-022-02995-9	polycrystalline, commercial
LiNi0.8Mn0.1Co0.1O2	211	3.0-4.5		0.1	0.33	4.5	50	64	0.720						90	5	5	10.1021/acsami.7b13597	polycrystalline, benchmark, commercial
LiNi0.8Mn0.1Co0.1O2	189	3.0-4.3	85	0.1	1	4.3	200	83	0.085		174	167			88	7	5	10.1016/j.ssi.2022.116031	polycrystalline, synthesis, lab-made
LiNi0.8Mn0.1Co0.1O2	195	3.0-4.3	86	0.1	1	4.3	200	89	0.055		177	170			88	7	5		polycrystalline, synthesis, lab-made
LiNi0.8Mn0.1Co0.1O2	201	3.0-4.3	86	0.1	1	4.3	132	80	0.152		183	174			88	7	5		polycrystalline, synthesis, lab-made
LiNi0.8Mn0.1Co0.1O2	203	2.8-4.3	96	0.1						183				0.74	80	10	10	10.1002/anie.202012773	single crystal, commercial
LiNi0.8Mn0.1Co0.1O2	200	3.0-4.3		0.1	0.1	4.3	50	82	0.360					2.4	80	10	10	10.1021/acsaem.0c02278	single crystal, synthesis, lab-made
LiNi0.80Mn0.15Co0.05O2	185	3.0-4.3		0.1										2.8	80	10	10		single crystal, synthesis, lab-made
LiNi0.85Mn0.10Co0.05O2	205	3.0-4.3		0.1	0.1	4.3	50	83	0.340					4.5	80	10	10		single crystal, synthesis, lab-made
LiNi0.8Mn0.1Co0.1O2	213	3.0-4.6		0.1	0.1	4.3	50	76	0.480					2.4	80	10	10		single crystal, synthesis, lab-made
LiNi0.80Mn0.15Co0.05O2	203	3.0-4.6		0.1	0.1	4.3	50	83	0.340					2.8	80	10	10		single crystal, synthesis, lab-made
LiNi0.85Mn0.10Co0.05O2	222	3.0-4.6		0.1	0.1	4.3	50	74	0.520					4.5	80	10	10		single crystal, synthesis, lab-made
LiNi0.8Mn0.1Co0.1O2	212	3.0-4.5	89	0.1	1	4.5	100	86	0.140					2.9	94	3	3	10.1149/1945-7111/aca2e5	polycrystalline, benchmark, lab-made
LiNi0.8Mn0.1Co0.1O2	204	2.8-4.3		0.1	1	4.3	100	75	0.250	155	133	109	68		85	10	5	10.1039/c7se00513j	polycrystalline, benchmark, lab-made
LiNi0.8Mn0.1Co0.1O2	176	2.5-4.3	90	0.5	0.5	4.3	100	74	0.260	154	120	105	80	3.51	75	20	5	10.1016/j.apsusc.2021.151663	polycrystalline, benchmark, commercial
LiNi0.8Mn0.1Co0.1O2	192	3.0-4.3	89	0.09	0.9	4.3	80	85	0.188	176			157		95	2	3	10.1016/j.jallcom.2022.166678	polycrystalline, benchmark, lab-made
LiNi0.8Mn0.1Co0.1O2	196	2.5-4.3	87	0.2	0.2	4.3	100	83	0.170	179	160	139	116	4.44	80	10	10	10.1016/j.jallcom.2023.168778	polycrystalline, benchmark, lab-made
					0.5	4.3	200	47	0.265					4.44	80	10	10		
LiNi0.8Mn0.1Co0.1O2	202	2.8-4.3		0.1	1	4.3	300	85	0.050	187	177	156		1.6	80	10	10	10.1016/j.ceramint.2019.06.134	polycrystalline, synthesis, lab-made
LiNi0.8Mn0.1Co0.1O2	203	2.8-4.3		0.1	1	4.3	300	87	0.043	190	181	163		0.2	80	10	10		polycrystalline, synthesis, lab-made
LiNi0.8Mn0.1Co0.1O2	185	2.8-4.35	90	1	1	4.35	100	82	0.180						80	10	10	10.1088/1757-899X/735/1/012007	polycrystalline, benchmark, lab-made
LiNi0.8Mn0.1Co0.1O2	199	3.0-4.3	88	0.2	0.1	4.4	500	81	0.038						80	10	10	10.1021/ja053675g	polycrystalline, synthesis, lab-made
LiNi0.8Mn0.1Co0.1O2	204	3.0-4.3	93	0.2	0.5	4.3	60	84	0.267		186	178	133		85	7.5	7.5	10.1149/1.2776160	polycrystalline, synthesis, lab-made
LiNi0.8Mn0.1Co0.1O2	201	2.8-4.3	84	0.09	0.9	4.3	100	81	0.190	180	172	161			80	10	10	10.1016/j.jallcom.2014.06.049	polycrystalline, synthesis, lab-made
LiNi0.8Mn0.1Co0.1O2	202	3.0-4.3	88	0.1	0.1	4.3	50	94	0.120	190	180	157			80	10	10	10.1149/1.2735916	polycrystalline, synthesis, lab-made
LiNi0.8Mn0.1Co0.1O2	201	2.8-4.3		0.1	0.2	4.3	100	90	0.100	179	168	150	123		80	10	10	10.1021/acsami.7b18933	polycrystalline, synthesis, lab-made
					1	4.3	100	91	0.090						80	10	10		
LiNi0.8Mn0.1Co0.1O2	190	2.8-4.3		0.1	0.2	4.3	100	87	0.130	198	185	175	164		80	10	10		polycrystalline, synthesis, lab-made
					0.2	4.3	100	94	0.060						80	10	10		
LiNi0.8Mn0.1Co0.1O2	203	3.0-4.3		0.1	1	4.3	500	78	0.044	187	180	172	157		92	4	4	10.1002/adfm.202105343	polycrystalline, benchmark, commercial
					3	4.3	387	80	0.052						92	4	4		
					5	4.3	358	80	0.056						92	4	4		
LiNi0.8Mn0.1Co0.1O2	194	3.0-4.4	95	1	1	4.4	100	86	0.140					3.18	80	10	10	10.1016/j.jallcom.2018.09.237	polycrystalline, benchmark, lab-made

LiNi0.8Mn0.1Co0.1O2					8	4.4	100	84	0.160					3.18	80	10	10	10.1016/j.jallcom.2018.09.237	polycrystalline, benchmark, lab-made
LiNi0.8Mn0.1Co0.1O2	206	3.0-4.3		0.1	1	4.3	50	63	0.740	174	150	85			80	10	10	10.1016/j.apsusc.2019.06.242	polycrystalline, benchmark, commercial
LiNi0.8Mn0.1Co0.1O2	212	3.0-4.5	85	0.2	0.2	4.5	40	85	0.375						80	10	10	10.1021/cm061746k	polycrystalline, benchmark, commercial
LiNi0.8Mn0.1Co0.1O2	216	3.0-4.5		0.1	0.1	4.5	100	66	0.340	181	147	97			85	10	5	10.1016/j.apsusc.2020.145580	polycrystalline, benchmark, lab-made
LiNi0.8Mn0.1Co0.1O2	199	2.8-4.3	83	0.1	1	4.3	80	92	0.100	175	163	152	127		80	10	10	10.1016/S1003-6326(15)63838-9	polycrystalline, synthesis, lab-made
LiNi0.8Mn0.1Co0.1O2	188	2.8-4.3	86	0.2	1	4.3	100	72	0.280	180	171	161	148		80	10	10	10.1021/acsaem.1c00827	polycrystalline, benchmark, commercial
LiNi0.8Mn0.1Co0.1O2	188	2.8-4.3	86	0.2	1	4.3	100	72	0.280	180	171	161	148		80	10	10	10.1021/acsaem.0c02865	polycrystalline, benchmark, commercial
LiNi0.8Mn0.1Co0.1O2	198	2.7-4.3	86	0.1	1	4.3	100	83	0.170						80	10	10	10.1016/j.jpowsour.2022.231244	polycrystalline, synthesis, lab-made
LiNi0.8Mn0.1Co0.1O2	185	3.0-4.3		0.5	0.5	4.3	50	94	0.120	188	105				90	5	5	10.1166/jnn.2017.15109	polycrystalline, benchmark, commercial
LiNi0.8Mn0.1Co0.1O2	170	3.0-4.2	100	0.1	1	4.2	1000	55	0.045						80	10	10	10.1021/acsomega.2c05521	polycrystalline, synthesis, lab-made
LiNi0.8Mn0.1Co0.1O2	188	2.8-4.3	83	0.1	0.1	4.3	62	97	0.048	180	176	172		2.7	92	4	4	10.1039/d2dt02246j	polycrystalline, benchmark, commercial
LiNi0.8Mn0.1Co0.1O2	192	2.7-4.3		0.1	1	4.3	300	73	0.090	170	160	153	144		80	10	10	10.1016/j.apsusc.2023.156379	polycrystalline, synthesis, lab-made
LiNi0.8Mn0.1Co0.1O2	183	2.7-4.3		0.1	1	4.3	300	70	0.100	176	171	162	150		80	10	10		polycrystalline, synthesis, lab-made
LiNi0.8Mn0.1Co0.1O2	191	2.7-4.3		0.1	1	4.3	300	86	0.047	181	175	167	153		80	10	10		polycrystalline, synthesis, lab-made
LiNi0.8Mn0.1Co0.1O2	207	2.7-4.3		0.1	1	4.3	300	82	0.060	195	188	175	158		80	10	10		polycrystalline, synthesis, lab-made
LiNi0.8Mn0.1Co0.1O2	181	2.7-4.3		0.1	1	4.3	300	85	0.050	170	160	153	144		80	10	10		polycrystalline, synthesis, lab-made
LiNi0.8Mn0.1Co0.1O2	168	2.8-4.3		1	1	4.3	100	70	0.300	162	150	147	114		80	10	10	10.1002/slct.202200812	polycrystalline, synthesis, lab-made
LiNi0.8Mn0.1Co0.1O2	192	2.7-4.3	75	0.09	0.09	4.3	40	92	0.200		150	140	123	6.8	80	10	10	10.1016/j.powtec.2010.09.010	polycrystalline, synthesis, lab-made
LiNi0.8Mn0.1Co0.1O2	184	2.7-4.3	74	0.09	0.09	4.3	40	81	0.475					7.6	80	10	10		polycrystalline, synthesis, lab-made
LiNi0.8Mn0.1Co0.1O2	175	2.7-4.3	73	0.09	0.09	4.3	40	70	0.750					13.4	80	10	10		polycrystalline, synthesis, lab-made
LiNi0.8Mn0.1Co0.1O2	149	2.7-4.3	69	0.09	0.09	4.3	40	54	1.150					16.6	80	10	10		polycrystalline, synthesis, lab-made
LiNi0.8Mn0.1Co0.1O2	138	2.7-4.3	71	0.09	0.09	4.3	40	50	1.250					18.8	80	10	10		polycrystalline, synthesis, lab-made
LiNi0.8Mn0.1Co0.1O2	187	2.7-4.3	88	0.1	1	4.3	100	96	0.040		172		156	5.00	80	10	10	10.1557/jmr.2019.307	polycrystalline, synthesis, lab-made
LiNi0.8Mn0.1Co0.1O2	203	2.7-4.5	89	0.1	1	4.5	100	88	0.120					5.00	80	10	10		polycrystalline, synthesis, lab-made
LiNi0.8Mn0.1Co0.1O2	200	2.8-4.3	87	0.1	2	4.3	200	72	0.140	182	173	163	141	1.6	80	10	10	10.1016/S1003-6326(14)63564-0	polycrystalline, synthesis, lab-made
LiNi0.8Mn0.1Co0.1O2	194	2.8-4.3	88	0.2	0.5	4.3	100	78	0.220	183	175	166	133		80	10	10	10.1142/S1793292019501030	polycrystalline, synthesis, lab-made
LiNi0.8Mn0.1Co0.1O2	242	2.5-4.25	97	0.1	0.5	4.25	100	72	0.280						80	10	10	10.1109/ITEC-AP.2016.7513031	polycrystalline, synthesis, lab-made
LiNi0.8Mn0.1Co0.1O2	173	3.0-4.5	91	0.1	1	4.3	60	86	0.233						85	12	3	10.4028/www.scientific.net/AMR.512-515.2028	polycrystalline, synthesis, lab-made
LiNi0.8Mn0.1Co0.1O2	180	2.8-4.3	76	0.1	1	4.3	100	84	0.160	152	137	119	89	6.34	80	15	5	10.1007/s12274-022-4532-y	polycrystalline, synthesis, lab-made
LiNi0.8Mn0.1Co0.1O2	206	2.8-4.3	83	0.1	1	4.3	100	90	0.100	193	175	165	138	3.14	80	15	5		polycrystalline, synthesis, lab-made
LiNi0.8Mn0.1Co0.1O2	211	2.8-4.3	89	0.1	1	4.3	100	93	0.070	205	191	175	150	2.24	80	15	5		polycrystalline, synthesis, lab-made
LiNi0.8Mn0.1Co0.1O2	196	2.8-4.3	86	0.1	1	4.3	100	92	0.080	185	173	160	134	1.67	80	15	5		polycrystalline, synthesis, lab-made
LiNi0.8Mn0.1Co0.1O2	183	2.8-4.3	83	0.1	1	4.3	100	87	0.130	174	169	152	131	1.37	80	15	5		polycrystalline, synthesis, lab-made
LiNi0.8Mn0.1Co0.1O2	205	2.8-4.3	87	0.1	1	4.3	60	86	0.233	190	177	143	38		80	10	10	10.1021/acssuschemeng.7b02178	polycrystalline, benchmark, commercial
LiNi0.8Mn0.1Co0.1O2	210	2.8-4.5	84	0.1	1	4.5	60	82	0.300						80	10	10		polycrystalline, benchmark, commercial
LiNi0.8Mn0.1Co0.1O2	203	3.0-4.3	87	0.1	0.5	4.3	100	94	0.060	180	167	148	109	3.61	80	10	10	10.1021/acsaem.8b00994	polycrystalline, synthesis, lab-made
LiNi0.8Mn0.1Co0.1O2	203	3.0-4.3	86	0.1	0.5	4.3	100	97	0.030	185	176	163	129	3.37	80	10	10		polycrystalline, synthesis, lab-made
LiNi0.8Mn0.1Co0.1O2	204	3.0-4.3	88	0.1	0.5	4.3	100	99	0.010					3.29	80	10	10		polycrystalline, synthesis, lab-made
LiNi0.8Mn0.1Co0.1O2	203	3.0-4.3	86	0.1	0.5	4.3	100	96	0.040					2.52	80	10	10		polycrystalline, synthesis, lab-made
LiNi0.8Mn0.1Co0.1O2	216	3.0-4.8	87	0.1	0.5	4.8	100	85	0.150					3.61	80	10	10		polycrystalline, synthesis, lab-made
LiNi0.8Mn0.1Co0.1O2	219	3.0-4.8	86	0.1	0.5	4.8	100	89	0.110					3.37	80	10	10		polycrystalline, synthesis, lab-made
LiNi0.8Mn0.1Co0.1O2	219	3.0-4.8	87	0.1	0.5	4.8	100	94	0.060					3.29	80	10	10		polycrystalline, synthesis, lab-made
LiNi0.8Mn0.1Co0.1O2	216	3.0-4.8	86	0.1	0.5	4.8	100	87	0.130					2.52	80	10	10		polycrystalline, synthesis, lab-made
LiNi0.8Mn0.1Co0.1O2	185	3.0-4.3	84	0.09	1	4.3	50	97	0.060	174	167				80	10	10	10.15625/0866-7144.2016-00394	polycrystalline, synthesis, lab-made
LiNi0.8Mn0.1Co0.1O2	182	3.0-4.3	86	0.09	1	4.3	50	82	0.360	169	160				80	10	10		polycrystalline, synthesis, lab-made
LiNi0.8Mn0.1Co0.1O2	199	2.8-4.3	93	0.19	0.19	4.3	50	95	0.100						80	10	10	10.2991/icmea-17.2018.3	polycrystalline, synthesis, lab-made
LiNi0.8Mn0.1Co0.1O2	188	2.8-4.3		0.1	1	4.3	300	55	0.150	164	160	143	117	6.00	80	10	10	10.1021/acsaem.1c02526	polycrystalline, benchmark, commercial
LiNi0.8Mn0.1Co0.1O2	181	3.0-4.3	82	0.1	0.1	4.3	100	64	0.360									10.1142/S1793604721500028	polycrystalline, benchmark, lab-made
LiNi0.8Mn0.1Co0.1O2	199	3.0-4.5	97	0.2	0.2	4.5	100	96	0.040						89	6	5	10.20964/2020.10.65	polycrystalline, synthesis, lab-made
LiNi0.8Mn0.1Co0.1O2	185	2.8-4.3		0.1	0.5	4.3	200	64	0.180	171	157	144	116		80	10	10	10.1088/1755-1315/474/5/052039	polycrystalline, synthesis, lab-made
LiNi0.8Mn0.1Co0.1O2	204	3.0-4.3	87	0.2	2	4.3	100	95	0.050	197	192	186	172	2.14	92.5	5	2.5	10.1007/s12613-021-2296-0	polycrystalline, synthesis, lab-made
					2	4.4	100	91	0.090					2.14	92.5	5	2.5		
LiNi0.8Mn0.1Co0.1O2	192	3.0-4.3	87	0.2	2	4.3	100	95	0.050	183	176	168	157	2.24	92.5	5	2.5		polycrystalline, synthesis, lab-made
					2	4.4	100	93	0.070					2.24	92.5	5	2.5		
LiNi0.8Mn0.1Co0.1O2	185	3.0-4.3	85	0.2	2	4.3	100	95	0.050	178	172	166	152	2.41	92.5	5	2.5		single crystal, synthesis, lab-made
					2	4.4	100	95	0.050					2.41	92.5	5	2.5		
LiNi0.8Mn0.1Co0.1O2	204	2.8-4.3	89	0.1	1	4.3	300	52	0.160	187	176	162	130	3.2	90	5	5	10.1039/D1TA00124H	polycrystalline, benchmark, lab-made
LiNi0.8Mn0.1Co0.1O2	182	3.0-4.3	87	0.1	1	4.3	50	91	0.180	170	164				80	10	10	10.15625/0866-7144.2016-00270	polycrystalline, synthesis, lab-made
LiNi0.8Mn0.1Co0.1O2	186	3.0-4.3	87	0.1	1	4.3	50	97	0.060	174	167				80	10	10		polycrystalline, synthesis, lab-made
LiNi0.8Mn0.1Co0.1O2	181	3.0-4.3	87	0.1	1	4.3	50	95	0.100	169	162				80	10	10		polycrystalline, synthesis, lab-made
LiNi0.8Mn0.1Co0.1O2	186	2.8-4.35		0.2	0.2	4.35	50	85	0.300	169	160	151	135					10.3389/fenrg.2019.00125	polycrystalline, benchmark, lab-made
					1	4.35	50	74	0.520										
LiNi0.8Mn0.1Co0.1O2	199	3.0-4.3	88	0.1	1	4.3	150	59	0.273					3.96	92.5	5	2.5	10.20964/2020.06.03	polycrystalline, synthesis, lab-made
LiNi0.8Mn0.1Co0.1O2	205	3.0-4.3	88	0.1	1	4.3	150	96	0.027					4.13	92.5	5	2.5		single crystal, synthesis, lab-made
LiNi0.8Mn0.1Co0.1O2	200	2.7-4.3	87	0.2	1	4.3	100	82	0.180	190	183	168	154		92.5	5	2.5		polycrystalline, benchmark, lab-made
LiNi0.8Mn0.1Co0.1O2	203	2.8-4.3	83	0.1	1	4.3	100	71	0.290	189	183	169	153	4.98	80	10	10	10.1016/j.ceramint.2020.09.143	polycrystalline, benchmark, lab-made
LiNi0.8Mn0.1Co0.1O2	180	3.0-4.3	82	0.138	0.138	4.3	100	87	0.130	177	156	128			94	3	3	10.1149/1945-7111/acbc51	polycrystalline, synthesis, lab-made
LiNi0.8Mn0.1Co0.1O2	201	3.0-4.35		0.2	0.2	4.35	20	96	0.200						80	10	10	10.1016/S1003-6326(15)63759-1	polycrystalline, synthesis, lab-made
LiNi0.8Mn0.1Co0.1O2	206	2.7-4.3	92	0.1	0.5	4.3	100	95	0.050						90	5.5	4.5	10.1021/acs.chemmater.7b05269	polycrystalline, synthesis, lab-made
LiNi0.8Mn0.1Co0.1O2	202	2.8-4.3	86	0.1	0.2	4.3	50	88	0.240	180	165	154	140		80	10	10	10.1016/S1003-6326(16)64244-9	polycrystalline, synthesis, lab-made
LiNi0.8Mn0.1Co0.1O2	192	2.8-4.3	85	0.2						183	177	166	135					10.1088/1755-1315/252/2/022053	polycrystalline, synthesis, lab-made
LiNi0.8Mn0.1Co0.1O2	187	2.8-4.3	83	0.2	1	4.3	100	84	0.160	181	176	164	144		80	10	10	10.1016/j.electacta.2021.138440	polycrystalline, benchmark, commercial
LiNi0.8Mn0.1Co0.1O2	197	2.8-4.4	91	0.2	1	4.3	200	75	0.125						85	10	5	10.1021/acsami.2c13832	polycrystalline, synthesis, lab-made
					1	4.5	200	75	0.125						85	10	5		
LiNi0.8Mn0.1Co0.1O2	198	2.8-4.4	94	0.2	1	4.3	200	83	0.085						85	10	5		polycrystalline, synthesis, lab-made
					1	4.5	200	74	0.130						85	10	5		
LiNi0.8Mn0.1Co0.1O2	205	2.8-4.4	92	0.2	1	4.3	200	92	0.040					1.16	85	10	5		single crystal, synthesis, lab-made
					1	4.5	200	83	0.085					1.16	85	10	5		
LiNi0.83Mn0.06Co0.11O2	196	2.7-4.3	90	0.1	1	4.3	100	93	0.070	182	176	170	162	2.39	80	10	10	10.1016/j.cej.2021.133731	single crystal, synthesis, lab-made
					1	4.5	100	81	0.190					2.39	80	10	10		
					5	4.3	100	78	0.220					2.39	80	10	10		
LiNi0.83Mn0.05Co0.12O2	194	2.7-4.3	83	0.1	1	4.3	100	79	0.210		169	153	128	6.75	80	10	10	10.1016/j.jmst.2022.01.037	polycrystalline, benchmark, lab-made

LiNi0.8Mn0.1Co0.1O2	176	3.0-4.3	78	0.5	0.5	4.3	200	77	0.115						80	10	10	10.1016/j.cej.2022.139431	single crystal, benchmark, lab-made
					2	4.3	300	91	0.030						80	10	10		
LiNi0.8Mn0.1Co0.1O2	220	2.8-4.3	99	0.1	0.5	4.3	200	59	0.205	200	192	175	158		80	10	10	10.1021/acsaem.0c00859	polycrystalline, benchmark, lab-made
LiNi0.8Mn0.1Co0.1O2	206	2.8-4.3	88	0.1	2	4.3	200	81	0.095	187	172	168	146	4.49	80	10	10	10.1021/acsami.8b11112	polycrystalline, benchmark, lab-made
LiNi0.8Mn0.1Co0.1O2	220	2.8-4.5	87	0.1	2	4.5	100	86	0.140					4.49	80	10	10		polycrystalline, benchmark, lab-made
LiNi0.8Mn0.1Co0.1O2	195	2.5-4.3	95	0.1	1	4.3	100	69	0.310	183	175		144		80	10	10	10.1016/j.cej.2020.127150	polycrystalline, synthesis, lab-made
					1	4.5	100	23	0.770						80	10	10		
LiNi0.8Mn0.1Co0.1O2	200	2.5-4.3	99	0.1	1	4.3	100	85	0.150	190	177		160		80	10	10		polycrystalline, synthesis, lab-made
					1	4.5	100	58	0.420						80	10	10		
LiNi0.8Mn0.1Co0.1O2	187	2.8-4.3	84	0.1	1	4.3	100	81	0.190	170	157	148	138		80	10	10	10.1021/acssuschemeng.2c05777	polycrystalline, benchmark, commercial
LiNi0.8Mn0.1Co0.1O2	202	3.0-4.5	98	0.085	0.425	4.5	50	64	0.720									10.33961/jecst.2020.01445	polycrystalline, benchmark, lab-made
LiNi0.8Mn0.1Co0.1O2	205	3.0-4.3	91	0.1	1	4.3	100	84	0.160	190	184		149		92.5	5	2.5	10.20964/2021.01.59	polycrystalline, benchmark, lab-made
LiNi0.8Mn0.1Co0.1O2	225	2.7-4.3	90	0.1	1	4.3	80	90	0.125		190	166	127		80	10	10	10.1007/s11664-018-6453-9	polycrystalline, benchmark, lab-made
					5	4.3	80	76	0.300						80	10	10		
LiNi0.8Mn0.1Co0.1O2	192	2.7-4.3	82	0.2	1	4.3	100	89	0.110	180	165	146	104	3.1	80	10	10	10.1039/C8DT00893K	polycrystalline, synthesis, lab-made
LiNi0.8Mn0.1Co0.1O2	202	3.0-4.5	84	0.25	0.25	4.5	100	69	0.310						80	10	10	10.1021/acsaem.9b02008	polycrystalline, benchmark, lab-made
LiNi0.8Mn0.1Co0.1O2	188	3.0-4.3	97	0.032	0.4	4.3	120	53	0.392	160	149	135		7.5	90	5	5	10.1021/acsaem.1c03000	polycrystalline, benchmark, lab-made
LiNi0.8Mn0.1Co0.1O2	209	2.7-4.3	93	0.1	1	4.3	100	60	0.400	184	168		128		80	10	10	10.6023/A21120600	polycrystalline, benchmark, lab-made
LiNi0.8Mn0.1Co0.1O2	201	3.0-4.3	83	0.09	0.9	4.3	200	45	0.275						80	10	10	10.1149/1945-7111/ac5655	polycrystalline, benchmark, lab-made
LiNi0.8Mn0.1Co0.1O2	189	2.7-4.3		0.1	0.1	4.3	100	78	0.220	177	157		131	3.2	80	10	10	10.1016/j.apsusc.2020.148034	polycrystalline, synthesis, lab-made
LiN0.787Mn0.098Co0.099O2	202	2.7-4.3		0.1	0.1	4.3	100	81	0.190	197	177		156	3.17	80	10	10		polycrystalline, synthesis, lab-made
LiNi0.790Mn0.099Co0.099O2	220	2.7-4.3		0.1	0.1	4.3	100	82	0.180	205	181		153	3.15	80	10	10		polycrystalline, synthesis, lab-made
LiNi0.8Mn0.1Co0.1O2	186	3.0-4.3	85	0.1	1	4.3	50	81	0.380	168	160		126		80	10	10	10.15625/0866-7144.2016-00400	polycrystalline, synthesis, lab-made
LiNi0.8Mn0.1Co0.1O2	204	2.7-4.3	79	0.1	1	4.3	200	72	0.140	204	186	164	142		80	10	10	10.1016/j.ceramint.2021.10.116	polycrystalline, benchmark, lab-made
LiNi0.83Mn0.06Co0.11O2	210	2.7-4.3	84	0.1	1	4.3	100	88	0.120	197	190	176	154		70	20	10	10.1016/j.matchemphys.2023.127521	polycrystalline, benchmark, lab-made
LiNi0.8Mn0.1Co0.1O2	188	2.5-4.3		0.1	0.2	4.3	100	82	0.180	155	134	110	60	12.48	80	10	10	10.1002/celc.202101654	polycrystalline, benchmark, lab-made
LiNi0.8Mn0.1Co0.1O2	204	2.7-4.3	90	0.1	0.5	4.3	50	73	0.540						90	5	5	10.1016/j.jallcom.2021.159079	polycrystalline, benchmark, lab-made
LiNi0.8Mn0.1Co0.1O2	219	2.7-4.6			0.5	4.6	100	44	0.560						90	5	5		polycrystalline, benchmark, lab-made
LiNi0.8Mn0.1Co0.1O2	215	2.8-4.3	85	0.1	1	4.3	200	65	0.175	203	192	163	133		80	10	10	10.1016/j.jcis.2022.08.061	polycrystalline, benchmark, commercial
LiNi0.8Mn0.1Co0.1O2	235	2.7-4.5		0.2	1	4.5	700	39	0.087					3.48	70	20	10	10.1016/j.ensm.2022.01.056	polycrystalline, benchmark, lab-made
					1	4.7	500	44	0.112					3.48	70	20	10		
LiNi0.8Mn0.1Co0.1O2		2.7-4.3								188	181	173	149	3.48	70	20	10		polycrystalline, benchmark, lab-made
LiNi0.8Mn0.1Co0.1O2	198	3.0-4.6	94	0.2	0.2	4.6	80	51	0.613						90	5	5	10.1039/D1RA00857A	polycrystalline, benchmark, commercial
LiNi0.8Mn0.1Co0.1O2	219	2.7-4.8	81	0.1	2	4.8	200	67	0.165						80	10	10	10.1016/j.electacta.2021.138775	polycrystalline, benchmark, lab-made
LiNi0.8Mn0.1Co0.1O2	186	2.8-4.3	79	0.09	0.9	4.3	100	85	0.150		150	133	126	2.1	75	15	10	10.1002/aenm.202203999	polycrystalline, benchmark, lab-made
LiNi0.8Mn0.1Co0.1O2	201	2.8-4.5	78	0.09	0.9	4.5	100	80	0.200					2.1	75	15	10		polycrystalline, benchmark, lab-made
LiNi0.8Mn0.1Co0.1O2	206	3.0-4.4	78	0.09	0.9	4.4	100	84	0.160	186	179	173			80	10	10	10.1007/s11581-022-04662-9	polycrystalline, benchmark, lab-made
LiNi0.8Mn0.1Co0.1O2	216	3.0-4.5	85	0.1	1	4.5	100	70	0.300					2.6	80	10	10	10.1016/j.jallcom.2022.164286	polycrystalline, benchmark, commercial
LiNi0.8Mn0.1Co0.1O2	192	3.0-4.3	85	0.0925	0.925	4.3	100	90	0.100	175	163		135		80	10	10	10.1007/s10008-017-3863-1	polycrystalline, benchmark, lab-made
LiNi0.8Mn0.1Co0.1O2	209	3.0-4.5	91	0.0925	0.925	4.5	100	50	0.500						80	10	10		polycrystalline, benchmark, lab-made
LiNi0.83Mn0.05Co0.12O2	186	2.8-4.3	83	0.1	1	4.3	100	83	0.170	178	171	164	154	2.8	80	10	10	10.1021/acssuschemeng.1c07637	polycrystalline, benchmark, lab-made
LiNi0.83Mn0.05Co0.12O2	219	2.8-4.5	88	0.1	1	4.3	100	63	0.370					2.8	80	10	10		polycrystalline, benchmark, lab-made
LiNi0.85Mn0.1Co0.05O2	186	2.75-4.2		0.18	0.2	4.2	100	73	0.270						90	4	6	10.1149/2.0151908jes	polycrystalline, synthesis, lab-made
					1	4.2	200	80	0.100						90	4	6		
LiNi0.85Mn0.1Co0.05O2	190	2.75-4.2		0.18	0.2	4.2	100	85	0.150						90	4	6		polycrystalline, synthesis, lab-made
					1	4.2	200	82	0.090						90	4	6		
LiNi0.85Mn0.1Co0.05O2	197	2.75-4.2		0.18	0.2	4.2	100	84	0.160						90	4	6		polycrystalline, synthesis, lab-made
					1	4.2	200	88	0.060						90	4	6		
LiNi0.8Mn0.1Co0.1O2	193	2.7-4.3		0.1	0.5	4.3	100	76	0.240	170	156	138	106		80	10	10	10.1149/2.1301914jes	polycrystalline, benchmark, lab-made
LiNi0.8Mn0.1Co0.1O2	198	2.7-4.3	84	0.18	0.9	4.3	200	59	0.205		145		120		84	8	8	10.1007/s11581-020-03758-4	polycrystalline, benchmark, lab-made
LiNi0.8Mn0.1Co0.1O2	203	2.7-4.6	86	0.09	0.9	4.3	100	75	0.250						60	20	20	10.1007/s11581-020-03893-y	polycrystalline, benchmark, lab-made
					0.9	4.6	100	65	0.350						60	20	20		
LiNi0.8Mn0.1Co0.1O2	100	3.0-4.3	73	0.1	0.2	4.3	100	53	0.470					7.53	80	10	10	10.1016/j.jallcom.2023.169358	single crystal, synthesis, lab-made
LiNi0.8Mn0.1Co0.1O2	138	3.0-4.3	80	0.1	0.2	4.3	100	53	0.470					3.91	80	10	10		single crystal, synthesis, lab-made
LiNi0.8Mn0.1Co0.1O2	173	3.0-4.3	77	0.1	0.2	4.3	100	59	0.410					3.73	80	10	10		single crystal, synthesis, lab-made
LiNi0.8Mn0.1Co0.1O2	176	3.0-4.3	77	0.1	0.2	4.3	100	72	0.280					2.86	80	10	10		single crystal, synthesis, lab-made
LiNi0.8Mn0.1Co0.1O2	187	3.0-4.3	85	0.1	0.2	4.3	100	78	0.220					1.86	80	10	10		single crystal, synthesis, lab-made
LiNi0.8Mn0.1Co0.1O2	203	3.0-4.3	99	0.1	0.1	4.3	50	87	0.260	173	153	137	88					10.1016/j.scp.2022.100959	polycrystalline, synthesis, lab-made
					1	4.3	100	90	0.100										
LiNi0.8Mn0.1Co0.1O2	205	2.7-4.3	82	0.05	10	4.3	100	85	0.150	182	167	148	115	5.21	80	10	10	10.1016/j.jallcom.2022.168575	polycrystalline, benchmark, lab-made
LiNi0.8Mn0.1Co0.1O2	176	2.8-4.3	87	0.1	2	4.3	100	79	0.210	175	162	144	124		80	10	10	10.1016/j.matlet.2022.133768	polycrystalline, benchmark, commercial
LiNi0.8Mn0.1Co0.1O2	201	2.7-4.5	99	0.09	0.5	4.5	500	26	0.148					3.58	80	10	10	10.1016/j.jtice.2023.104730	polycrystalline, benchmark, lab-made
LiNi0.83Mn0.06Co0.11O2	182	2.8-4.3	77	1	1	4.3	100	77	0.230	178	166	148	107		80	10	10	10.1016/j.jelechem.2023.117202	single crystal, benchmark, lab-made
LiNi0.8Mn0.1Co0.1O2	200	2.7-4.3		0.1	0.5	4.3	50	95	0.100						80	10	10	10.1016/j.jechem.2022.11.016	single crystal, synthesis, lab-made
LiNi0.8Mn0.1Co0.1O2	210	2.7-4.7		0.1	0.5	4.3	50	80	0.400						80	10	10		single crystal, synthesis, lab-made
LiNi0.8Mn0.1Co0.1O2	219	2.7-4.9		0.1	0.5	4.3	50	69	0.620						80	10	10		single crystal, synthesis, lab-made
LiNi0.8Mn0.1Co0.1O2	200	3.0-4.3	86	0.2	1	4.3	100	82	0.180	191	180		158	2.34	90	5	5	10.1016/j.surfin.2022.102480	polycrystalline, benchmark, commercial
LiNi0.8Mn0.1Co0.1O2	182	2.8-4.3		0.2	0.5	4.3	1000	71	0.029	167	152	142	125		80	10	10	10.1002/adfm.202211171	single crystal, commercial
LiNi0.8Mn0.1Co0.1O2	214	2.5-4.4	89	0.1	1	4.4	200	77	0.115						70	20	10	10.1021/acssuschemeng.2c05779	polycrystalline, benchmark
LiNi0.8Mn0.1Co0.1O2	223	2.7-4.5	82	0.11	1.1	4.5	400	53	0.118						85	10	5	10.1016/j.cej.2022.139405	polycrystalline, benchmark, lab-made
LiNi0.8Mn0.1Co0.1O2	196	2.8-4.3		0.2	1	4.3	100	72	0.280	188	171	148	104	9.68	80	10	10	10.1007/s10008-023-05427-8	polycrystalline, benchmark, lab-made
LiNi0.8Mn0.1Co0.1O2	211	3.0-4.3	99	0.1	0.5	4.5	200	54	0.230	200	188	182		3.97	80	10	10	10.1007/s13391-023-00414-7	polycrystalline, benchmark, lab-made
LiNi0.8Mn0.1Co0.1O2	211	2.8-4.3	91	0.06	0.95	4.3	200	55	0.225		180	165			80	10	10	10.1016/j.mtener.2022.101207	polycrystalline, benchmark, commercial
					3.8	4.3	200	58	0.210						80	10	10		
LiNi0.8Mn0.1Co0.1O2	202	2.8-4.3	85	0.1	1	4.3	200	61	0.195	178	173	165	152		80	10	10	10.1016/j.electacta.2022.141474	single crystal, benchmark, commercial
LiNi0.8Mn0.1Co0.1O2	208	2.8-4.5	85	0.1	1	4.5	100	58	0.420						80	10	10		single crystal, benchmark, commercial
LiNi0.8Mn0.1Co0.1O2	200	2.5-4.3		0.1	0.2	4.3	100	80	0.200	172	158	152	121		92	5	3	10.1016/j.jechem.2022.09.008	polycrystalline, commercial
					1	4.3	450	61	0.087						92	5	3		
					0.2	4.5	100	51	0.490						92	5	3		
					0.2	4.7	100	31	0.690						92	5	3		
LiNi0.8Mn0.1Co0.1O2	178	2.8-4.3	88	0.5	0.5	4.3	200	58	0.210	177	164	137	106		80	10	10	10.1007/s12274-022-4754-z	polycrystalline, benchmark, lab-made
LiNi0.8Mn0.1Co0.1O2	185	2.8-4.3	85	0.1	1	4.3	500	45	0.110	158	143	126	95		80	10	10	10.1016/j.jallcom.2022.166967	polycrystalline, benchmark, commercial

LiNi0.8Mn0.1Co0.1O2	187	3.0-4.2		0.1	1	4.2	200	75	0.125					1.77	90	5	5	10.1016/j.cej.2022.138382	polycrystalline, synthesis, lab-made
LiNi0.8Mn0.1Co0.1O2	195	3.0-4.2		0.1	1	4.2	200	80	0.100					1.84	90	5	5		polycrystalline, synthesis, lab-made
LiNi0.8Mn0.1Co0.1O2	198	2.5-4.3	85	0.1	1	4.3	500	68	0.064	174	157	147	118		92	5	3	10.1016/j.cej.2022.137959	polycrystalline, benchmark, commercial
					0.2	4.3	100	83	0.170						92	5	3		
					0.2	4.7	100	34	0.660						92	5	3		
LiNi0.8Mn0.1Co0.1O2	223	2.5-4.7	84	0.1	1	4.7	125	77	0.184						92	5	3		polycrystalline, benchmark, commercial
LiNi0.8Mn0.1Co0.1O2	214	2.7-4.3	88	0.1	1	4.3	300	35	0.217	174	158	145	102		80	10	10	10.1016/j.electacta.2022.141411	polycrystalline, benchmark, lab-made
LiNi0.8Mn0.1Co0.1O2	195	2.7-4.5	87	0.11	1.1	4.5	300	42	0.193					3.81	90	5	5	10.1002/adfm.202206428	polycrystalline, benchmark, lab-made
LiNi0.8Mn0.1Co0.1O2	209	3.0-4.5	84	0.125	1.25	4.5	100	78	0.220						80	10	10	10.1007/s42864-022-00178-x	polycrystalline, benchmark, commercial
LiNi0.8Mn0.1Co0.1O2	184	3.0-4.3	80	0.1	1	4.3	200	68	0.160	140	130	118	90		80	10	10	10.1002/ente.202200843	polycrystalline, benchmark, commercial
LiNi0.776Mn0.117Co0.097O2	202	2.8-4.55	82	0.1	0.3	4.55	200	68	0.160					6.00	80	10	10	10.1016/j.ensm.2022.09.008	polycrystalline, synthesis, lab-made
LiNi0.8Mn0.1Co0.1O2	205	2.7-4.3	82	0.09	1	4.3	100	60	0.400	183	167	148	115	5.21	80	10	10	10.1016/j.jallcom.2022.166317	polycrystalline, benchmark, lab-made
LiNi0.8Mn0.1Co0.1O2	169	2.8-4.5	91	0.1	1	4.5	100	90	0.100						80	10	10	10.1039/D2NR04773J	polycrystalline, benchmark, commercial
					5	4.5	100	68	0.320						80	10	10		
LiNi0.8Mn0.1Co0.1O2	197	2.7-4.3	72	0.1	1	4.3	200	62	0.190	188	176	157	118	2.16	85	7	8	10.1016/j.cej.2022.137663	polycrystalline, benchmark, lab-made
					1	4.3	200	61	0.195					2.16	85	7	8		
LiNi0.8Mn0.1Co0.1O2	179	3.0-4.2	99	0.085	0.85	4.2	100	75	0.250						85	5	10	10.1039/D2TA07322F	polycrystalline, benchmark, commercial
LiNi0.8Mn0.1Co0.1O2	193	3.0-4.3	88	0.1	0.5	4.3	100	93	0.070	180	170	153	117	4.19	90	5	5	10.1016/j.jallcom.2022.165488	polycrystalline, benchmark, lab-made
LiNi0.8Mn0.1Co0.1O2	191	2.8-4.3		0.2	3	4.3	120	80	0.167		175		126		95	3	2	10.1016/j.cej.2022.137051	polycrystalline, benchmark
LiNi0.8Mn0.1Co0.1O2	163	2.7-4.3	90	0.1	0.5	4.3	100	65	0.350	145	136	123	102		80	10	10	10.3390/nano12203610	polycrystalline, synthesis, lab-made
LiNi0.8Mn0.1Co0.1O2	176	2.8-4.3	94	0.5	0.5	4.3	200	47	0.265	187	170	136	117		80	10	10	10.1016/j.electacta.2022.140745	polycrystalline, benchmark, commercial
LiNi0.8Mn0.1Co0.1O2	213	2.7-4.3	81	0.05	1	4.3	100	58	0.420	179	166	143	104	9.1	80	10	10	10.1016/j.jallcom.2022.164712	polycrystalline, benchmark, lab-made
					1	4.5	100	49	0.510					9.1	80	10	10		
LiNi0.8Mn0.1Co0.1O2	196	2.7-4.3	91	0.1	1	4.3	79	80	0.253	181	173		140	2.00	80	10	10	10.1021/acsami.2c06264	polycrystalline, benchmark, commercial
LiNi0.8Mn0.1Co0.1O2	175	2.7-4.3	87	0.1	1	4.3	300	61	0.130	170	162	153	138	5.29	80	10	10	10.1016/j.ceramint.2022.02.289	polycrystalline, synthesis, lab-made
					5	4.3	300	51	0.163					5.29	80	10	10		
					10	4.3	300	57	0.143					5.29	80	10	10		
LiNi0.8Mn0.1Co0.1O2	203	2.7-4.3	89	0.1	1	4.3	300	63	0.123	182	175	164	151	4.29	80	10	10		polycrystalline, synthesis, lab-made
					5	4.3	300	46	0.180					4.29	80	10	10		
					10	4.3	300	46	0.180					4.29	80	10	10		
LiNi0.8Mn0.1Co0.1O2	197	2.7-4.3	87	0.1	1	4.3	300	74	0.087	185	178	175	163	3.57	80	10	10		polycrystalline, synthesis, lab-made
					5	4.3	300	55	0.150					3.57	80	10	10		
					10	4.3	300	60	0.133					3.57	80	10	10		
LiNi0.8Mn0.1Co0.1O2	163	2.8-4.3	88	0.1	1	4.3	100	88	0.120	144	136	124	98		80	10	10	10.1016/j.jcis.2022.02.095	polycrystalline, benchmark, commercial
					5	4.3	100	68	0.320						80	10	10		
LiNi0.8Mn0.1Co0.1O2	190	2.8-4.3	74	0.1	1	4.3	200	70	0.150	185	181	163	105	3.77	80	10	10	10.1016/j.ceramint.2022.03.020	single crystal, benchmark
LiNi0.8Mn0.1Co0.1O2	201	3.0-4.4	85	0.1	1	4.4	200	51	0.245					3.5	80	10	10	10.1021/acsami.2c04666	polycrystalline, benchmark, lab-made
LiNi0.8Mn0.1Co0.1O2	193	2.5-4.3		0.2	1	4.3	100	73	0.270	185	175		143	1.68	80	10	10	10.1021/acssuschemeng.2c01712	polycrystalline, benchmark, commercial
LiNi0.8Mn0.1Co0.1O2	191	2.8-4.3		0.1	1	4.3	300	57	0.143	176	166	157	138		80	10	10	10.1007/s40145-022-0582-6	polycrystalline, benchmark
LiNi0.8Mn0.1Co0.1O2	213	2.8-4.3	86	0.1	1	4.3	300	82	0.060	200	190	175	150		80	10	10	10.1002/adsu.202200002	polycrystalline, benchmark, commercial
LiNi0.8Mn0.1Co0.1O2	209	2.8-4.3	87	0.09	0.9	4.3	100	73	0.270	190	178	163	148		80	10	10	10.1021/acs.iecr.2c00181	polycrystalline, benchmark, lab-made
LiNi0.8Mn0.1Co0.1O2	208	2.6-4.3	85	0.1	1	4.3	300	49	0.170	172	155	135	90					10.1016/j.jallcom.2021.162848	polycrystalline, benchmark, commercial
					1	4.5	300	44	0.187										
LiNi0.8Mn0.1Co0.1O2	211	3.0-4.4	86	0.09	0.9	4.4	300	66	0.113					3.25	80	10	10	10.1016/j.jpowsour.2022.231035	polycrystalline, benchmark, lab-made
					0.9	4.5	200	51	0.245					3.25	80	10	10		
LiNi0.8Mn0.1Co0.1O2	200	2.7-4.3		0.2	1	4.3	200	69	0.155	187	180	152	107		80	10	10	10.3390/coatings12030319	polycrystalline, benchmark, commercial
LiNi0.8Mn0.1Co0.1O2	201	2.8-4.3	89	0.09	1	4.3	500	67	0.066	176	165	150	119		80	10	10	10.1007/s11581-021-04375-5	polycrystalline, benchmark, lab-made
LiNi0.8Mn0.1Co0.1O2	190	2.8-4.5	94	0.1	0.25	4.5	500	28	0.144						80	10	10	10.1016/j.apsusc.2021.151716	polycrystalline, benchmark, commercial
LiNi0.8Mn0.1Co0.1O2	200<br>197	2.8-4.5	83<br>86	0.1<br>0.2	0.1	4.5	100	77	0.230						80	10	10	10.1016/j.ceramint.2021.11.118	polycrystalline, benchmark, lab-made
					0.2	4.5	100	79	0.210						80	10	10		
					0.5	4.5	100	75	0.250						80	10	10		
					1	4.5	200	38	0.310						80	10	10		
LiNi0.8Mn0.1Co0.1O2	210	3.0-4.4	81	0.09	0.9	4.4	100	83	0.170						80	10	10	10.1016/j.jallcom.2021.162155	polycrystalline, benchmark, lab-made
LiNi0.8Mn0.1Co0.1O2	222	2.75-4.5	84	0.1	1	4.5	300	35	0.217						85	10	5	10.1016/j.cej.2021.132985	polycrystalline, benchmark, lab-made
LiNi0.8Mn0.1Co0.1O2	215	2.8-4.3	96	0.1	0.33	4.3	100	56	0.440		187	175			80	10	10	10.1002/smll.202104625	polycrystalline, benchmark, commercial
					1	4.3	200	62	0.190						80	10	10		
LiNi0.8Mn0.1Co0.1O2	184	2.8-4.3	98	0.1	1	4.3	100	69	0.310	170	160	147	115	2.65	80	10	10	10.1016/j.jre.2020.12.010	polycrystalline, benchmark, lab-made
LiNi0.8Mn0.1Co0.1O2	189	2.8-4.3	70	0.1	1	4.3	100	70	0.300	183	172	148	117		80	10	10	10.1021/acsaem.1c02841	polycrystalline, benchmark, lab-made
LiNi0.8Mn0.1Co0.1O2	216	2.8-4.5	69	0.1	1	4.5	100	58	0.420						80	10	10		polycrystalline, benchmark, lab-made
LiNi0.8Mn0.1Co0.1O2	198	2.8-4.3		0.2	1	4.3	100	87	0.130	189	181	159	134	2.52	80	10	10	10.1021/acsaem.2c00658	polycrystalline, synthesis, lab-made
LiNi0.8Mn0.1Co0.1O2	200	2.8-4.3		0.2	1	4.3	100	93	0.070	193	186	170	151	1.89	80	10	10		single crystal, synthesis, lab-made
LiNi0.8Mn0.1Co0.1O2	195	2.8-4.3	82	0.09	0.45	4.3	50	63	0.740	132	114	105	83		70	20	10	10.3390/ma15010396	polycrystalline, benchmark, lab-made
					1.8	4.3	100	58	0.420						70	20	10		
LiNi0.813Mn0.093Co0.094O2	192	2.8-4.3		0.1	0.5	4.5	100	81	0.190	175	162	152	118		80	10	10	10.1021/acsaem.1c03257	polycrystalline, benchmark, lab-made
LiNi0.8Mn0.1Co0.1O2	205	2.8-4.3	77	0.1	0.5	4.3	200	65	0.175	180	169	154	130		80	10	10	10.1016/j.jallcom.2021.161594	polycrystalline, benchmark, commercial
					0.5	4.5	200	57	0.215						80	10	10		
LiNi0.8Mn0.1Co0.1O2	209	3.0-4.5	83	0.1	1	4.3	200	74	0.130						80	10	10	10.1039/D1NR05912B	polycrystalline, benchmark, commercial
					1	4.5	100	68	0.320						80	10	10		
LiNi0.8Mn0.1Co0.1O2	200	2.7-4.3	72	0.1	0.1	4.3	100	73	0.270	173	165	132		2.81	80	10	10	10.1016/j.jechem.2021.08.012	polycrystalline, benchmark, lab-made
					1	4.3	500	56	0.088					2.81	80	10	10		
					5	4.3	100	54	0.460					2.81	80	10	10		
LiNi0.8Mn0.1Co0.1O2	203	2.7-4.3	80	0.1	1	4.3	100	58	0.420	181	172	152	131		80	10	10	10.1016/j.ceramint.2021.08.167	polycrystalline, benchmark, lab-made
LiNi0.8Mn0.1Co0.1O2	204	2.8-4.3		0.2	0.2	4.3	100	57	0.430	183	172	157	126		80	10	10	10.1002/celc.202101230	polycrystalline, benchmark, commercial
LiNi0.8Mn0.1Co0.1O2	201	2.8-4.3	88	0.09	0.9	4.3	500	65	0.070						80	10	10	10.1002/smll.202102981	polycrystalline, benchmark, lab-made
					0.9	4.5	500	62	0.076						80	10	10		
LiNi0.8Mn0.1Co0.1O2	200	3.0-4.3	89	0.1	0.1	4.3	50	90	0.200						90	5	5	10.1016/j.ssi.2021.115734	polycrystalline, benchmark, commercial
LiNi0.8Mn0.1Co0.1O2	210	2.7-4.5		0.1	1	4.5	100	87	0.130						80	10	10	10.1134/S0036024421100277	polycrystalline, synthesis, lab-made
LiNi0.8Mn0.1Co0.1O2	209	3.0-4.3	92	0.1	0.5	4.3	100	74	0.260						90	5	5	10.1007/s12598-021-01710-7	polycrystalline, benchmark, commercial
					1	4.3	100	76	0.240						90	5	5		
LiNi0.8Mn0.1Co0.1O2	205	2.8-4.3	85	0.18	0.9	4.3	100	78	0.220	195	185	177	166		80	10	10		polycrystalline, synthesis, lab-made
LiNi0.8Mn0.1Co0.1O2	212	2.8-4.5	82	0.18	0.9	4.5	100	54	0.460						80	10	10		polycrystalline, synthesis, lab-made

LiNi0.8Mn0.1Co0.1O2	195	2.8-4.3	86	0.18	0.9	4.3	100	91	0.090	187	172	163	145		80	10	10	10.1016/j.ceramint.2021.05.294	single crystal, synthesis, lab-made
LiNi0.8Mn0.1Co0.1O2	202	2.8-4.5	83	0.18	0.9	4.5	100	82	0.180						80	10	10		single crystal, synthesis, lab-made
LiNi0.8Mn0.1Co0.1O2	197	2.8-4.3	89	0.18	0.9	4.3	100	95	0.050	187	176	168	155		80	10	10		single crystal, synthesis, lab-made
LiNi0.8Mn0.1Co0.1O2	204	2.8-4.5	85	0.18	0.9	4.5	100	91	0.090						80	10	10		single crystal, synthesis, lab-made
LiNi0.8Mn0.1Co0.1O2	207	2.7-4.3	85	0.1	0.5	4.3	100	65	0.350						80	10	10	10.1115/1.4048491	polycrystalline, benchmark, lab-made
LiNi0.8Mn0.1Co0.1O2	192	3.0-4.3	89	0.2											93	3.5	3.5	10.1016/j.jallcom.2021.159259	polycrystalline, benchmark, commercial
LiNi0.8Mn0.1Co0.1O2	192	2.7-4.5	89	0.1	1	4.5	200	73	0.135						80	10	10	10.1007/s10008-021-04984-0	polycrystalline, benchmark, lab-made
LiNi0.8Mn0.1Co0.1O2	184	2.8-4.3	82	0.425	0.5	4.3	100	70	0.300						80	10	10	10.1021/acssuschemeng.1c00802	polycrystalline, benchmark, lab-made
LiNi0.8Mn0.1Co0.1O2	269	2.5-4.6		0.2	0.2	4.6	100	34	0.660						80	10	10	10.1007/s11581-021-04036-7	polycrystalline, benchmark, commercial
LiNi0.8Mn0.1Co0.1O2	206	2.7-4.3	92	0.1	1	4.3	300	68	0.107	187	176		139		80	10	10	10.1039/D1TA02563E	polycrystalline, benchmark, commercial
LiNi0.8Mn0.1Co0.1O2	170	2.7-4.3		0.8	0.8	4.5	200	74	0.130	156	130		104	1.4	80	10	10	10.1021/acs.jpcc.1c01727	polycrystalline, benchmark, lab-made
LiNi0.8Mn0.1Co0.1O2	186	2.8-4.3	81	0.1	0.5	4.3	400	39	0.153	155	143	127	108	5.32	70	20	10	10.6023/A21010019	polycrystalline, synthesis, lab-made
LiNi0.8Mn0.1Co0.1O2	190	2.8-4.3	81	0.1	0.5	4.3	400	83	0.043	170	160	150	129	3.89	70	20	10		polycrystalline, synthesis, lab-made
LiNi0.8Mn0.1Co0.1O2	190	2.8-4.3		0.2	3	4.3	300	65	0.117	187	177	166	135		80	10	10	10.1149/1945-7111/abf4ed	polycrystalline, benchmark, lab-made
LiNi0.8Mn0.1Co0.1O2	212	2.7-4.6		0.18	0.9	4.6	100	54	0.460						80	10	10	10.1007/s11581-021-03997-z	polycrystalline, benchmark, lab-made
LiNi0.8Mn0.1Co0.1O2	207	2.8-4.3	85	0.1	2	4.3	500	54	0.092	194	186	173	147		80	10	10	10.1016/j.ceramint.2021.01.161	polycrystalline, benchmark, lab-made
					1	4.2	1000	53	0.047						80	10	10		
LiNi0.85Mn0.05Co0.05O2	188	2.8-4.3		0.3	0.3	4.3	60	74	0.433	186	175	163		5.00	88	8	4	10.3390/ma14082070	polycrystalline, benchmark, lab-made
LiNi0.8Mn0.1Co0.1O2	178	2.8-4.3	84	0.1	0.5	4.3	100	82	0.180	167	161	148	121	5.82	80	10	10	10.1002/er.6296	polycrystalline, synthesis, lab-made
LiNi0.8Mn0.1Co0.1O2	173	2.8-4.3	82	0.1	0.5	4.3	100	75	0.250	163	155	140	112	9.26	80	10	10		polycrystalline, synthesis, lab-made
LiNi0.8Mn0.1Co0.1O2	203	2.8-4.3	91	0.1	0.5	4.3	100	92	0.080	193	183	168	144	1.08	80	10	10		polycrystalline, synthesis, lab-made
LiNi0.8Mn0.1Co0.1O2	195	2.8-4.3	89	0.1	0.5	4.3	100	83	0.170	182	173	156	132	4.64	80	10	10		polycrystalline, synthesis, lab-made
LiNi0.8Mn0.1Co0.1O2	160	3.0-4.5	62	0.18	0.9	4.5	100	59	0.410					7.86	80	10	10	10.1016/j.jallcom.2020.158325	polycrystalline, benchmark, lab-made
LiNi0.8Mn0.1Co0.1O2	183	3.0-4.5	87	0.18	0.9	4.5	100	71	0.290					3.35	80	10	10		polycrystalline, benchmark, lab-made
LiNi0.8Mn0.1Co0.1O2	183	2.8-4.3	85	0.1	0.5	4.3	110	47	0.482						90	5	5	10.1039/D0CP06422J	polycrystalline, benchmark, commercial
LiNi0.8Mn0.1Co0.1O2	197	2.8-4.3		0.2	1	4.3	100	74	0.260						80	10	10	10.1016/j.ceramint.2020.10.213	polycrystalline, benchmark, commercial
LiNi0.8Mn0.1Co0.1O2	233	2.8-4.6	84	0.2	1	4.6	50	75	0.500						80	10	10		polycrystalline, benchmark, commercial
LiNi0.8Mn0.1Co0.1O2	197	2.7-4.3	78	0.05	1	4.3	100	62	0.380	170	149	114	70		80	10	10	10.1021/acsomega.1c01552	solid state, benchmark for La and Al doping
LiNi0.8Mn0.1Co0.1O2	199	2.8-4.3	90	0.1	1	4.3	50	77	0.460	186	177	165	142		90	5	5	10.1016/j.proci.2020.06.332	polycrystalline, benchmark, lab-made
LiNi0.8Mn0.1Co0.1O2	181	2.8-4.3	87	0.1	1	4.3	200	72	0.140	176	163	150	137	10.7	80	10	10	10.1007/s10853-020-05306-x	single crystal, benchmark, lab-made
					1	4.6	250	67	0.132					10.7	80	10	10		
LiNi0.8Mn0.1Co0.1O2	195	3.0-4.3	88	0.1	0.5	4.3	50	91	0.180						95	2	3	10.1021/acsaem.0c02258	polycrystalline, benchmark, lab-made
LiNi0.8Mn0.1Co0.1O2	195	2.75-4.3	80	0.09	0.9	4.3	200	88	0.060	183	176	171	163	1.3	80	10	10	10.1016/j.ssi.2020.115504	polycrystalline, benchmark, lab-made
					0.9	4.5	200	73	0.135					1.3	80	10	10		
LiNi0.8Mn0.1Co0.1O2	204	2.8-4.3	93	0.09	0.9	4.3	200	86	0.070		185	177	165	3.27	80	10	10	10.1016/j.jpowsour.2020.228701	polycrystalline, synthesis, lab-made
					4.5	4.3	100	94	0.060					3.27	80	10	10		
LiNi0.8Mn0.1Co0.1O2	198	2.8-4.3	90	0.09	0.9	4.3	200	66	0.170		174	166	152	5.59	80	10	10		polycrystalline, synthesis, lab-made
					4.5	4.3	100	76	0.240					5.59	80	10	10		
LiNi0.8Mn0.1Co0.1O2	186	2.8-4.3	83	0.09	0.9	4.3	200	34	0.330		153	137	115	7.41	80	10	10		polycrystalline, synthesis, lab-made
					4.5	4.3	100	70	0.300					7.41	80	10	10		
LiNi0.8Mn0.1Co0.1O2	195	2.7-4.3	89	1	1	4.3	200	83	0.085	180	167	155	134					10.1088/1742-6596/1676/1/012001	polycrystalline, benchmark, lab-made
LiNi0.8Mn0.1Co0.1O2	195	3.0-4.5	77	0.5	0.5	4.5	200	57	0.215						80	10	10	10.1016/j.nanoen.2020.105034	polycrystalline, benchmark, commercial
LiNi0.8Mn0.1Co0.1O2	213	2.5-4.6	81	0.1	1	4.6	100	83	0.170						80	10	10	10.1007/s10008-020-04742-8	polycrystalline, benchmark, lab-made
LiNi0.8Mn0.1Co0.1O2	199	2.7-4.3	83	0.1	0.1	4.3	100	74	0.260	175	167	140		2.01	80	10	10	10.1039/D0TA00924E	polycrystalline, benchmark, lab-made
					1	4.3	500	63	0.074					2.01	80	10	10		
LiNi0.8Mn0.1Co0.1O2	205	2.7-4.4	83	0.1										2.01	80	10	10		polycrystalline, benchmark, lab-made
LiNi0.8Mn0.1Co0.1O2	218	2.7-4.5	83	0.1	1	4.5	180	63	0.206					2.01	80	10	10		polycrystalline, benchmark, lab-made
LiNi0.8Mn0.1Co0.1O2	224	2.7-4.6	82	0.1										2.01	80	10	10		polycrystalline, benchmark, lab-made
LiNi0.8Mn0.1Co0.1O2	193	2.8-4.3	85	0.22	0.22	4.3	100	93	0.070	181	170	158			80	10	10	10.1016/j.ceramint.2020.05.160	polycrystalline, benchmark, lab-made
LiNi0.8Mn0.1Co0.1O2	191	3.0-4.3	84	0.135	1.35	4.3	350	81	0.054	165	158	149	133		80	10	10	10.1016/j.matchemphys.2020.123085	polycrystalline, benchmark, lab-made
LiNi0.8Mn0.1Co0.1O2	200	3.0-4.3	100	0.2	0.2	4.3	20	90.8	0.460	192	177	165			80	10	10	10.1007/s11814-020-0570-x	polycrystalline, benchmark, commercial
LiNi0.8Mn0.1Co0.1O2	195	2.8-4.3	95	0.2	0.5	4.3	50	101	-0.020						80	10	10	10.1016/j.jechem.2019.10.027	polycrystalline, commercial
LiNi0.8Mn0.1Co0.1O2	208	2.8-4.6	97	0.2	0.5	4.6	50	93	0.140						80	10	10		polycrystalline, commercial
LiNi0.8Mn0.1Co0.1O2	211	2.8-4.9	89	0.2	0.5	4.6	50	82	0.360						80	10	10		polycrystalline, commercial
LiNi0.8Mn0.1Co0.1O2	226	2.8-4.6	73	0.1	0.2	4.6	150	82	0.120					2.01	80	10	10	10.1021/acsaem.0c00803	polycrystalline, synthesis, lab-made
					1	4.6	200	70	0.150					2.01	80	10	10		
					5	4.4	300	74	0.087					2.01	80	10	10		
					10	4.6	200	66	0.170					2.01	80	10	10		
LiNi0.8Mn0.1Co0.1O2	240	2.8-4.6	77	0.1	0.2	4.6	150	85	0.100					2.54	80	10	10		polycrystalline, synthesis, lab-made
					1	4.6	200	76	0.120					2.54	80	10	10		
					1	4.4	300	83	0.057					2.54	80	10	10		
					1	4.4	300	80	0.067					2.54	80	10	10		
LiNi0.8Mn0.1Co0.1O2	192	3.0-4.4	99	0.09	0.9	4.4	100	79	0.210						80	10	10	10.1016/j.matchemphys.2020.123135	polycrystalline, benchmark, lab-made
LiNi0.8Mn0.1Co0.1O2	184	2.7-4.3		0.1	1	4.3	100	65	0.350	170	162		142		80	13	7	10.1007/s11581-019-03399-2	polycrystalline, benchmark, lab-made
LiNi0.8Mn0.1Co0.1O2	180	2.8-4.3	90	0.1	1	4.3	300	80	0.067	174	170	162	149		80	10	10	10.1021/acssuschemeng.0c02729	polycrystalline, benchmark, commercial
LiNi0.8Mn0.1Co0.1O2	183	2.8-4.3	97	0.1	1	4.3	300	75	0.083	161	146	127			80	10	10	10.1007/s11581-020-03500-0	polycrystalline, synthesis, lab-made
LiNi0.8Mn0.1Co0.1O2	207	2.8-4.3		0.1	1	4.3	100	66	0.340	192	180	161	133		80	10	10	10.1002/cssc.201902219	polycrystalline, benchmark, lab-made
LiNi0.8Mn0.1Co0.1O2	175	2.8-4.3	82	0.3	0.3	4.5	100	73	0.270						85	10	5	10.1021/acsami.9b20872	polycrystalline, benchmark, lab-made
					0.3	4.3	100	83	0.170						85	10	5		
					1.8	4.5	100	72	0.280						85	10	5		
LiNi0.8Mn0.1Co0.1O2	208	3.0-4.7	88	0.1	0.5	4.3	110	87	0.118						80	10	10	10.1016/j.jechem.2019.05.013	polycrystalline, commercial
					0.5	4.5	110	73	0.245						80	10	10		
					0.5	4.7	110	42	0.527						80	10	10		
LiNi0.8Mn0.1Co0.1O2	200	2.8-4.3	84	0.1	1	4.3	90	88	0.133	186	174	159	142		80	10	10	10.1016/j.electacta.2019.135054	polycrystalline, benchmark, lab-made
LiNi0.8Mn0.1Co0.1O2	194	3.0-4.3	84	0.09	0.9	4.3	200	77	0.115	188	181	171			80	10	10	10.1016/j.jelechem.2019.113582	polycrystalline, benchmark, commercial
					7.2	4.3	200	75	0.125						80	10	10		
LiNi0.8Mn0.1Co0.1O2	194	2.8-4.3	85	0.1	1	4.3	200	93	0.035	189	184	173	159		80	10	10	10.1016/j.jpowsour.2019.227195	polycrystalline, benchmark, lab-made
LiNi0.8Mn0.1Co0.1O2	203	2.8-4.3		0.1	1	4.3	100	81	0.190	190	182	170	148	9.73	80	10	10	10.1166/mex.2019.1581	polycrystalline, benchmark, lab-made
					2	4.3	100	71	0.290					9.73	80	10	10		
					10	4.3	100	35	0.650					9.73	80	10	10		
LiNi0.8Mn0.1Co0.1O2	192	3.0-4.5	77	0.095	0.475	4.5	350	67	0.094					3.2	80	10	10	10.1016/j.jpowsour.2019.227017	polycrystalline, benchmark, lab-made
LiNi0.8Mn0.1Co0.1O2	199	2.8-4.5		0.2	1	4.5	200	37	0.315					2.01	80	10	10	10.1021/acsaem.9b01414	polycrystalline, synthesis, lab-made

LiNi0.8Mn0.1Co0.1O2	207	2.8-4.5		0.2	1	4.5	200	86	0.070					1.47	80	10	10	10.1021/acsaem.9b01414	polycrystalline, synthesis, lab-made
LiNi0.8Mn0.1Co0.1O2	175	3.0-4.5		0.2	5	4.5	500	49	0.102		157		101		80	10	10	10.1007/s12274-019-2469-6	polycrystalline, benchmark, lab-made
LiNi0.8Mn0.1Co0.1O2	174	2.8-4.3	78	0.1	0.5	4.3	100	77	0.230	165		157	145	4.42	80	10	10	10.1007/s10854-019-02174-3	polycrystalline, benchmark, lab-made
LiNi0.8Mn0.1Co0.1O2	197	2.8-4.3	90	0.1	1	4.3	200	49	0.255		185		113		80	10	10	10.1002/celc.201901208	polycrystalline, benchmark, commercial
LiNi0.8Mn0.1Co0.1O2	211	2.8-4.6	82	0.2	1	4.6	100	70	0.300					6.76	80	10	10	10.1016/j.mtener.2019.05.003	polycrystalline, benchmark, lab-made
LiNi0.8Mn0.1Co0.1O2	192	2.5-4.3	98	0.475	0.475	4.3	100	69	0.310	191	187				80	10	10	10.1016/j.ceramint.2019.04.092	polycrystalline, benchmark, commercial
LiNi0.8Mn0.1Co0.1O2	180	2.8-4.3	81	0.1	0.5	4.3	45	81	0.422						90	3	7	10.1021/acsaem.8b01892	polycrystalline, synthesis, lab-made
LiNi0.8Mn0.1Co0.1O2	204	3.0-4.3	92	0.1	1	4.3	100	40	0.600						94	3	3	10.1016/j.matchemphys.2018.09.076	polycrystalline, commercial
LiNi0.8Mn0.1Co0.1O2	180	2.8-4.3		0.1	0.2	4.3	60	80	0.333	163	150	133	103		80	10	10	10.1016/j.jpowsour.2018.10.094	polycrystalline, benchmark, lab-made
					2	4.3	60	69	0.517						80	10	10		
LiNi0.8Mn0.1Co0.1O2	148	2.8-4.3	58	0.2	0.2	4.3	60	66	0.567	137	120	90		11.37	80	10	10	10.1021/acsami.8b19349	polycrystalline, synthesis, lab-made
LiNi0.8Mn0.1Co0.085O2	144	2.8-4.3	60	0.2	0.2	4.3	60	38	1.033	103	71	30		8.69	80	10	10		polycrystalline, synthesis, lab-made
LiNi0.8Mn0.1Co0.075O2	141	2.8-4.3	61	0.2	0.2	4.3	60	50	0.833	100	75	38		8.59	80	10	10		polycrystalline, synthesis, lab-made
LiNi0.8Mn0.1Co0.1O2	181	3.0-4.3	91	0.1	1	4.3	100	70	0.300						90	5	5	10.1016/j.matchemphys.2018.04.078	polycrystalline, benchmark, commercial
LiNi0.8Mn0.1Co0.1O2	200	2.5-4.3	82	0.1	5	4.3	125	52	0.384	184	175	165	145		80	10	10	10.1007/s10854-018-9093-1	polycrystalline, benchmark, lab-made
LiNi0.8Mn0.1Co0.1O2	198	2.8-4.4	81	0.2	1	4.4	100	81	0.190						80	10	10	10.1039/C7NJ04936F	polycrystalline, benchmark, lab-made
LiNi0.8Mn0.1Co0.1O2	228	2.8-4.6	81	0.2	1	4.6	100	67	0.330						80	10	10		polycrystalline, benchmark, lab-made
LiNi0.8Mn0.1Co0.1O2	188	2.8-4.3	87	0.075	0.75	4.3	50	88	0.240	176	167	155	132		85	10	5	10.1016/j.ssi.2017.10.017	polycrystalline, benchmark, lab-made
					3.75	4.3	50	72	0.560						85	10	5		
LiNi0.8Mn0.1Co0.1O2	200	2.8-4.5	86	0.075	0.75	4.5	50	78	0.440						85	10	5		polycrystalline, benchmark, lab-made
					3.75	4.5	50	73	0.540						85	10	5		
LiNi0.8Mn0.1Co0.1O2	194	2.8-4.3	88	0.09	1.8	4.3	100	88	0.120		182	164	141		80	10	10	10.1016/j.apsusc.2014.01.121	polycrystalline, benchmark, commercial
LiNi0.8Mn0.1Co0.1O2	190	2.7-4.3	84	0.18	0.18	4.3	500	66	0.068	177	170	158	140		80	10	10	10.1016/j.jcis.2023.03.032	polycrystalline, benchmark, lab-made
					0.45	4.3	100	65	0.350						80	10	10		
LiNi0.8Mn0.1Co0.1O2	199	3.0-4.3	90	0.5	0.5	4.3	100	82	0.180		196	185			96	2	2	10.1016/j.ceramint.2022.12.065	polycrystalline, benchmark, lab-made
					0.5	4.6	100	64	0.360						96	2	2		
LiNi0.8Mn0.1Co0.1O2	169	3.0-4.3	91	0.1	0.1	4.3	20	95.9	0.205						80	15	5	https://www.jproeng.com/EN/Y2007/V7/I4/817	polycrystalline, synthesis, lab-made
LiNi0.8Mn0.1Co0.1O2	204	2.8-4.3	98	0.1	2	4.3	100	81	0.190	188	176	160		2.4	80	10	10	10.1016/j.jpowsour.2012.08.029	polycrystalline, synthesis, lab-made
LiNi0.8Mn0.1Co0.1O2	205	2.8-4.3	82	0.1	2	4.3	100	80	0.200	194	188	173	150		80	10	10	10.1016/j.powtec.2012.12.061	polycrystalline, benchmark, lab-made
LiNi0.8Mn0.1Co0.1O2	199	2.7-4.6	81	0.2	0.2	4.6	100	82	0.180					0.58	80	10	10	10.1016/j.cej.2023.142093	single crystal, benchmark, commercial
					5	4.6	200	79	0.105					0.58	80	10	10		
LiNi0.8Mn0.1Co0.1O2	210	2.8-4.5	87	0.05	0.5	4.5	100	82	0.180					2.99	80	10	10	10.1021/acsami.2c11773	polycrystalline, benchmark, lab-made
LiNi0.8Mn0.1Co0.1O2	195	2.8-4.3		0.2	1	4.3	200	62	0.190	189	164	148	111	9.68	80	10	10	10.1007/s11581-022-04572-w	polycrystalline, benchmark, lab-made
LiNi0.8Mn0.1Co0.1O2	205	3.0-4.35	98	0.2	1	4.35	200	68	0.160	199	185		134		80	10	10	10.1002/cssc.202200543	polycrystalline
LiNi0.8Mn0.1Co0.1O2	193	2.7-4.3	95	0.1	1	4.3	100	67	0.330	176	168		150	2.59	80	10	10	10.1007/s11581-023-04945-9	polycrystalline, benchmark, lab-made
LiNi0.83Mn0.05Co0.12O2	219	2.8-4.5	79	0.09	0.9	4.3	200	62	0.190						80	10	10	10.1007/s10008-022-05150-w	polycrystalline, benchmark, lab-made
LiNi0.8Mn0.1Co0.1O2	209	3.0-4.3	82	0.09	0.5	4.3	200	78	0.110	175	160	141	109		80	10	10	10.1007/s10008-023-05470-5	polycrystalline, benchmark, lab-made
LiNi0.8Mn0.1Co0.1O2	210	2.8-4.3	81	0.1	1	4.3	100	85	0.150	190	171	160	133		80	15	5	10.1007/s12274-021-3784-2	polycrystalline, synthesis, lab-made
LiNi0.8Mn0.1Co0.1O2	234	2.8-4.4	82	0.1	1	4.4	50	78	0.440						80	15	5		polycrystalline, synthesis, lab-made
LiNi0.8Mn0.1Co0.1O2	214	2.7-4.3	92	0.1	1	4.3	300	78	0.073		177	168			80	10	10	10.1007/s11581-020-03849-2	polycrystalline, synthesis, lab-made
LiNi0.83Mn0.12Co0.05O2	197	2.75-4.3	90	0.1	0.5	4.3	200	69	0.155						89	8.5	2.5	10.1016/j.jcis.2021.07.027	polycrystalline, benchmark, lab-made
					0.5	4.2	500	67	0.066						89	8.5	2.5		
LiNi0.83Mn0.12Co0.05O2	205	2.75-4.4		0.1	0.5	4.4	200	54	0.230						89	8.5	2.5		polycrystalline, benchmark, lab-made
LiNi0.8Mn0.1Co0.1O2	190	2.8-4.3	90	0.1	0.1	4.3	100	68	0.320					5.55	80	10	10	10.1016/j.electacta.2015.12.080	polycrystalline, benchmark, lab-made
LiNi0.8Mn0.1Co0.1O2	218	2.8-4.5	90	0.1	0.1	4.5	100	59	0.410					5.55	80	10	10		polycrystalline, benchmark, lab-made
LiNi0.8Mn0.1Co0.1O2	187	2.7-4.3	79	0.085	0.85	4.3	200	49	0.255						80	13	7	10.1016/j.electacta.2018.08.124	polycrystalline, benchmark
LiNi0.8Mn0.1Co0.1O2	180	3.0-4.3	90	0.1	0.1	4.3	50	91.3	0.174						92	4	4	10.1016/j.electacta.2018.09.103	polycrystalline, benchmark, lab-made
LiNi0.8Mn0.1Co0.1O2	173	2.7-4.2		0.1	0.2	4.2	30	99	0.033						80	10	10	10.1016/j.electacta.2019.06.034	polycrystalline, benchmark, commercial
LiNi0.8Mn0.1Co0.1O2	194	2.7-4.3		0.1	0.2	4.2	30	91	0.300						80	10	10		polycrystalline, benchmark, commercial
LiNi0.8Mn0.1Co0.1O2	200	2.7-4.4	85	0.1	0.2	4.4	100	30	0.700						80	10	10		polycrystalline, benchmark, commercial
LiNi0.8Mn0.1Co0.1O2	224	2.7-4.5		0.1	0.2	4.5	30	71	0.967						80	10	10		polycrystalline, benchmark, commercial
LiNi0.8Mn0.1Co0.1O2	193	2.8-4.3	82	0.09	0.9	4.3	100	75	0.250	179	168	152	120		80	10	10	10.1016/j.electacta.2020.136541	polycrystalline, benchmark, lab-made
LiNi0.8Mn0.1Co0.1O2	215	2.8-4.5		0.09	0.9	4.5	100	64	0.360						80	10	10		polycrystalline, benchmark, lab-made
LiNi0.8Mn0.1Co0.1O2	206	3.0-4.3	89	0.1	0.5	4.3	30	97	0.100						90	5	5	10.1016/j.electacta.2020.137380	polycrystalline, synthesis, lab-made
					0.5	4.35	400	54	0.115						90	5	5		
LiNi0.8Mn0.1Co0.1O2	219	3.0-4.5	88	0.1	0.5	4.5	30	86	0.467						90	5	5		polycrystalline, synthesis, lab-made
LiNi0.8Mn0.1Co0.1O2	245	3.0-4.9	90	0.1	0.1	4.9	15	81	1.267						90	5	5		polycrystalline, synthesis, lab-made
LiNi0.8Mn0.1Co0.1O2	205	3.0-4.3	88	0.1	0.5	4.3	30	99	0.033						90	5	5		single crystal, synthesis, lab-made
					0.5	4.35	400	85	0.038						90	5	5		
LiNi0.8Mn0.1Co0.1O2	219	3.0-4.5	89	0.1	0.5	4.5	30	95	0.167						90	5	5		single crystal, synthesis, lab-made
LiNi0.8Mn0.1Co0.1O2	246	3.0-4.9	91	0.1	0.1	4.9	15	93	0.467						90	5	5		single crystal, synthesis, lab-made
LiNi0.8Mn0.1Co0.1O2	203	2.8-4.3	84	0.18	1	4.3	100	81	0.190	193	183	172	159		80	10	10	10.1016/j.electacta.2021.138297	polycrystalline, benchmark, lab-made
LiNi0.8Mn0.1Co0.1O2	209	2.8-4.5	82	0.18	1	4.5	100	57	0.430						80	10	10		polycrystalline, benchmark, lab-made
LiNi0.8Mn0.1Co0.1O2	202	3.0-4.3	82	0.1	0.5	4.3	270	62	0.141	185	173		150		80	10	10	10.1016/j.electacta.2021.139468	polycrystalline, benchmark, lab-made
LiNi0.8Mn0.1Co0.1O2	195	2.8-4.3	84	0.1	1	4.3	200	72	0.140	177	172	165	155		88	7	5	10.1016/j.electacta.2021.139743	single crystal, benchmark, commercial
LiNi0.8Mn0.1Co0.1O2	203	2.8-4.5	84	0.1	1	4.5	100	77	0.230						88	7	5		single crystal, benchmark, commercial
LiNi0.8Mn0.1Co0.1O2	213	2.8-4.3	89	0.1	1	4.3	400	44	0.140	194	178	158	142		80	10	10	10.1016/j.electacta.2022.140436	polycrystalline, benchmark, lab-made
LiNi0.8Mn0.1Co0.1O2	210	2.8-4.3	87	0.1	1	4.3	200	80	0.100	200	190	177	127	2.16	80	10	10	10.1016/j.electacta.2022.140559	polycrystalline, benchmark, commercial
LiNi0.8Mn0.1Co0.1O2	201	2.7-4.3	86	0.1	0.1	4.3	40	71	0.725	147	114	82	40		80	10	10	10.1016/j.electacta.2022.140973	polycrystalline, benchmark, lab-made
					1	4.3	100	57	0.430						80	10	10		
LiNi0.83Mn0.06Co0.11O2	204	2.8-4.3	88	0.1	2	4.3	500	54	0.092	174	169	140	110	3.11	80	10	10	10.1016/j.jcis.2022.08.085	polycrystalline, benchmark, lab-made
LiNi0.83Mn0.06Co0.11O2	202	2.8-4.3	90	0.1	1	4.3	200	61	0.195	165	149	132	105	3.11	80	10	10	10.1016/j.jcis.2022.12.142	polycrystalline, benchmark, lab-made
LiNi0.8Mn0.1Co0.1O2	203	3.0-4.3	92	0.1	1	4.3	100	45	0.550						94	3	3	10.1016/j.materresbull.2020.111008	polycrystalline, benchmark, commercial
LiNi0.85Mn0.10Co0.05O2	204	2.5-4.3	86	0.1	1	4.3	300	84	0.053	183	168	135	102	4.89	85	10	5	10.1016/j.powtec.2021.08.083	polycrystalline, benchmark, lab-made
LiNi0.80Mn0.05Co0.15O2	214	2.8-4.35	96	0.095	0.1	4.3	100	93	0.070	195	187	178	169		82	8	10	10.1016/j.jpowsour.2020.228592	polycrystalline, benchmark, lab-made
LiNi0.8Mn0.1Co0.1O2	203	2.8-4.5	88	0.1	1	4.3	100	79	0.210					3.18	80	10	10	10.1016/j.jpowsour.2020.228699	polycrystalline, benchmark, lab-made
					5	4.3	250	70	0.120					3.18	80	10	10		
LiNi0.8Mn0.1Co0.1O2	192	3.0-4.3	86	0.1	0.3	4.3	150	39	0.407	181	174	155			90	5	5	10.1016/j.jpowsour.2020.229218	polycrystalline, benchmark, commercial
LiNi0.83Mn0.10Co0.07O2	208	3.0-4.3	92	0.1	0.5	4.3	100	63	0.370	206	195	178			90	5	5	10.1016/j.jpowsour.2022.232202	polycrystalline, benchmark, commercial
LiNi0.8Mn0.1Co0.1O2	200	2.8-4.3	98	0.1	0.1	4.3	100	88	0.120	170	150	140	97		80	10	10	10.1016/j.apt.2021.10.016	polycrystalline, benchmark, lab-made
LiNi0.8Mn0.1Co0.1O2	187	2.8-4.3	86	0.5	0.5	4.3	200	56	0.220						80	10	10	10.1016/j.jallcom.2021.160642	polycrystalline, benchmark, commercial
					5	4.3	300	26	0.247						80	10	10		

LiNi0.8Mn0.1Co0.1O2	168	2.8-4.3	81	0.5	0.5	4.3	200	75	0.125						80	10	10	10.1016/j.jallcom.2021.[illegible]	single crystal, synthesis, lab-made
					5	4.3	300	62	0.127						80	10	10		
LiNi0.8Mn0.05Co0.15O2	194	2.8-4.3	96	0.1	0.2	4.3	400	53	0.118	182	170	156	129		80	10	10	10.1016/j.cej.2021.129217	polycrystalline, benchmark, lab-made
LiNi0.8Mn0.1Co0.1O2	205	2.7-4.5	85	0.1	1	4.5	200	58	0.210					2.93	85	10	5	10.1016/j.cej.2022.138911	polycrystalline, benchmark, lab-made
					1	4.5	100	72	0.280					2.93	85	10	5		
LiNi0.8Mn0.1Co0.1O2	210	2.75-4.4	80	0.09	0.18	4.4	100	87	0.130						90	4	6	10.1016/j.ssi.2020.115292	polycrystalline, benchmark, lab-made
LiNi0.8Mn0.1Co0.1O2	185	2.75-4.3	85	0.09	0.9	4.3	300	80	0.067	164	153	147		4.26	80	10	10	10.1016/j.ssi.2020.115520	polycrystalline, benchmark, lab-made
LiNi0.8Mn0.1Co0.1O2	189	2.8-4.3	79	0.1	1	4.3	500	47	0.106	161	130	83			80	10	10	10.1016/j.apsusc.2018.05.027	polycrystalline, benchmark, lab-made
LiNi0.8Mn0.1Co0.1O2	179	2.8-4.3	92	1	1	4.3	300	80	0.067	176	171	161	132	2.48	90	5	5	10.1016/j.apsusc.2022.153928	single crystal, benchmark, commercial
					5	4.3	300	44	0.187					2.48	90	5	5		
LiNi0.8Mn0.1Co0.1O2	213	2.7-4.3	89	0.09	1	4.3	100	60	0.400	189	180	170	148		80	10	10	10.1016/j.jechem.2020.06.055	polycrystalline, benchmark, lab-made
LiNi0.8Mn0.1Co0.1O2	197	3.0-4.3	88	0.1	0.5	4.3	150	99	0.007						95	2.5	2.5	10.1016/j.nanoen.2019.104309	polycrystalline
LiNi0.83Mn0.05Co0.12O2	225	3.0-4.8	86	0.1											80	10	10	10.1016/j.nanoen.2022.107335	polycrystalline, commercial
LiNi0.83Mn0.05Co0.12O2	208	3.0-4.5	89	0.1											80	10	10		polycrystalline, commercial
LiNi0.8Mn0.1Co0.1O2	212	2.8-4.5	81	0.075	0.75	4.5	45	84.8	0.338						85	10	5	10.1016/j.ceramint.2018.10.219	polycrystalline, benchmark, commercial
LiNi0.83Mn0.06Co0.11O2	195	2.8-4.3	95	0.1	1	4.3	500	48	0.104	182	175	166	157		80	10	10	10.1016/j.ceramint.2021.08.373	polycrystalline, benchmark, lab-made
LiNi0.83Mn0.06Co0.11O2	216	2.7-4.3	76	0.09	0.9	4.3	100	87	0.130	175	160		101		70	20	10	10.1016/j.ceramint.2021.12.079	polycrystalline, benchmark, lab-made
LiNi0.8Mn0.1Co0.1O2	199	2.5-4.3		0.1	0.2	4.3	100	70	0.300	172	155	146	127		94	3	3	10.1016/j.apenergy.2022.119282	polycrystalline, commercial
					0.2	4.5	100	25	0.750						94	3	3		
LiNi0.8Mn0.1Co0.1O2	174	2.8-4.3	77	0.2	0.2	4.3	100	79	0.210					8.65	80	10	10	10.1016/j.jpowsour.2018.06.091	polycrystalline, benchmark, lab-made
					1	4.3	100	75	0.250					8.65	80	10	10		
LiNi0.8Mn0.1Co0.1O2	174	2.8-4.3	78	0.2	0.2	4.3	100	91	0.090					4.83	80	10	10		polycrystalline, synthesis, lab-made
					1	4.3	100	85	0.150					4.83	80	10	10		
LiNi0.8Mn0.1Co0.1O2	194	3.0-4.3	88	0.1	1	4.3	100	54	0.460						90	5	5	10.1016/j.jpowsour.2018.11.008	polycrystalline, benchmark, commercial
LiNi0.8Mn0.1Co0.1O2	198	3.0-4.3	88	0.18	0.9	4.3	50	83	0.340					3.2	80	10	10	10.1016/j.jpowsour.2018.11.053	polycrystalline, commercial
LiNi0.8Mn0.1Co0.1O2	223	3.0-4.7	89	0.18	0.9	4.7	50	77	0.460					3.2	80	10	10		polycrystalline, commercial
LiNi0.8Mn0.1Co0.1O2	186	2.8-4.3	81	0.2	0.5	4.3	200	65	0.175	175	158	149	136		80	10	10	10.1016/j.jpowsour.2018.11.062	polycrystalline, benchmark, lab-made
LiNi0.8Mn0.1Co0.1O2	186	3.0-4.3	93	0.09	0.9	4.3	100	85	0.150						90	5	5	10.1016/j.jpowsour.2019.01.050	polycrystalline, benchmark, commercial
LiNi0.82Mn0.09Co0.09O2	201	2.5–4.25	89	0.2	0.5	4.25	150	51	0.327						96	2	2	10.1016/j.jpowsour.2019.227625	polycrystalline, benchmark, commercial
LiNi0.83Mn0.06Co0.11O2	202	3.0-4.3	90	0.105	1.05	4.3	150	60	0.267	189	183		137	1.4	92.5	5	2.5	10.1016/j.jpowsour.2021.229857	polycrystalline, benchmark, lab-made
LiNi0.8Mn0.1Co0.1O2	211	2.8-4.5	88	0.1	0.5	4.5	100	84	0.160					3.19	80	10	10	10.1016/j.jpowsour.2022.231963	polycrystalline, synthesis, lab-made
LiNi0.8Mn0.1Co0.1O2	199	2.8-4.5	85	0.1	0.5	4.5	100	64	0.360					2.39	80	10	10		single crystal, synthesis, lab-made
LiNi0.8Mn0.1Co0.1O2	185	2.7-4.3	84	0.1	0.1	4.3	300	51	0.163	135	120	94		0.85	90	6	4	10.1016/j.jpowsour.2022.232150	polycrystalline, benchmark, commercial
LiNi0.8Mn0.1Co0.1O2	194	3.0-4.3	81	0.1	0.5	4.3	300	81	0.063	180	173		142	0.95	80	10	10	10.1016/j.jpowsour.2023.232774	polycrystalline, synthesis, lab-made
					1	4.3	700	48	0.074					0.95	80	10	10		
LiNi0.8Mn0.1Co0.1O2	191	3.0-4.3	81	0.1	0.5	4.3	100	80	0.200	173	160		134	1.21	80	10	10		single crystal, synthesis, lab-made
					1	4.3	300	64	0.120					1.21	80	10	10		
LiNi0.8Mn0.1Co0.1O2	190	3.0-4.3	81	0.1	0.5	4.3	100	91	0.090	154	143		110	2.33	80	10	10		single crystal, synthesis, lab-made
					1	4.3	300	77	0.077					2.33	80	10	10		
LiNi0.8Mn0.1Co0.1O2	194	3.0-4.3	82	0.1	0.5	4.3	100	96	0.040	156	147		122	0.53	80	10	10		single crystal, synthesis, lab-made
					0.5	4.3	300	88	0.040					0.53	80	10	10		
					1	4.3	300	87	0.043					0.53	80	10	10		
					1	4.3	700	63	0.053					0.53	80	10	10		
LiNi0.8Mn0.1Co0.1O2	196	3.0-4.3	81	0.1	0.5	4.3	100	96	0.040	154	143		119	3.27	80	10	10		single crystal, synthesis, lab-made
					1	4.3	300	83	0.057					3.27	80	10	10		
LiNi0.8Mn0.1Co0.1O2	199	2.8-4.5	83	0.09	1	4.5	50	84	0.320		177	170	160		85	10	5	10.1016/j.jallcom.2019.01.072	polycrystalline, benchmark, lab-made
LiNi0.8Mn0.1Co0.1O2	195	2.8-4.3	84	0.2	0.2	4.3	60	98	0.033					1.16	80	10	10	10.1016/j.jallcom.2019.05.065	polycrystalline, synthesis, lab-made
					1	4.3	60	94	0.100					1.16	80	10	10		
LiNi0.8Mn0.1Co0.1O2	198	2.8-4.3	84	0.2	0.2	4.3	60	88	0.200					1.83	80	10	10		polycrystalline, synthesis, lab-made
					1	4.3	60	87	0.217					1.83	80	10	10		
LiNi0.8Mn0.1Co0.1O2	195	2.8-4.3	82	0.2	0.2	4.3	60	87	0.217					2.04	80	10	10		polycrystalline, synthesis, lab-made
					1	4.3	60	83	0.283					2.04	80	10	10		
LiNi0.8Mn0.1Co0.1O2	216	2.8-4.6		0.2	0.2	4.3	120	84	0.133					1.16	80	10	10		polycrystalline, synthesis, lab-made
					1	4.3	120	83	0.142					1.16	80	10	10		
LiNi0.8Mn0.1Co0.1O2	217	2.8-4.6		0.2	0.2	4.3	120	78	0.183					1.83	80	10	10		polycrystalline, synthesis, lab-made
					1	4.3	120	81	0.158					1.83	80	10	10		
LiNi0.8Mn0.1Co0.1O2	214	2.8-4.6		0.2	0.2	4.3	120	69	0.258					2.04	80	10	10		polycrystalline, synthesis, lab-made
					1	4.3	120	70	0.250					2.04	80	10	10		
LiNi0.8Mn0.1Co0.1O2	203	2.8-4.5	86	0.1	1	4.5	50	88	0.240					2.75	85	10	5	10.1016/j.jallcom.2019.151683	polycrystalline, synthesis, lab-made
LiNi0.8Mn0.1Co0.1O2	211	2.8-4.5	87	0.1										3.78	85	10	5		polycrystalline, synthesis, lab-made
LiNi0.8Mn0.1Co0.1O2	192	2.8-4.3	72	0.1	0.5	4.3	500	24	0.152					3.8	80	10	10	10.1016/j.jallcom.2020.157877	polycrystalline, benchmark, commercial
LiNi0.8Mn0.1Co0.1O2	210	2.8-4.3	92	0.1	0.5	4.3	100	64	0.360	189	176	170			90	5	5	10.1016/j.jallcom.2021.159153	polycrystalline, benchmark, commercial
LiNi0.8Mn0.1Co0.1O2	183	2.7-4.3		0.1	0.2	4.3	100	82	0.180	160	151	125	81	4.51	80	10	10	10.1016/j.jallcom.2022.167862	polycrystalline, benchmark, lab-made
					1	4.3	200	73	0.135					4.51	80	10	10		
					5	4.3	200	68	0.160					4.51	80	10	10		
LiNi0.8Mn0.1Co0.1O2	197	2.7-4.5	81	0.1	1	4.5	200	46	0.270					4.51	80	10	10		polycrystalline, benchmark, lab-made
LiNi0.8Mn0.1Co0.1O2	187	2.8-4.4	81	0.1	0.5	4.4	100	56	0.440						80	10	10	10.1016/j.psep.2022.02.018	polycrystalline, benchmark, commercial
LiNi0.8Mn0.1Co0.1O2	195	2.7-4.3	82	0.1	1	4.3	100	68	0.320	183	177	168	156		80	10	10	10.1016/S1003-6326(22)66047-3	polycrystalline, benchmark, lab-made
					5	4.5	100	51	0.490						80	10	10		
LiNi0.8Mn0.1Co0.1O2	213	3.0-4.3		0.1	0.5	4.3	100	68	0.320	192	183	167			90	5	5	10.1016/j.jmst.2021.01.037	polycrystalline, benchmark
LiNi0.82Co0.13Mn0.05O2	205	2.75-4.35	82	0.1	1	4.35	250	54	0.184	194	186	159	135		96	2	2	10.1016/j.cej.2021.132908	polycrystalline, benchmark, lab-made
LiNi0.8Mn0.1Co0.1O2	202	3.0-4.3		0.1	0.5	4.3	100	86	0.140	185	171	157	140		96	2	2	10.1016/j.cap.2022.08.010	polycrystalline, benchmark, lab-made
LiNi0.8Mn0.1Co0.1O2	197	2.8-4.3	80	0.1	1	4.3	200	83	0.085						80	10	10	10.1016/j.jelechem.2020.114910	polycrystalline, benchmark, commercial
LiNi0.85Mn0.05Co0.10O2	208	3.0-4.3	90	0.05	0.33	4.3	50	84	0.320						96.5	1.5	2	10.1016/j.jelechem.2023.117242	polycrystalline, benchmark, lab-made
LiNi0.8Mn0.1Co0.1O2	195	2.7-4.4	90	0.1	1	4.4	120	67	0.275						94	3	3	10.1016/j.jtice.2019.06.006	polycrystalline, benchmark, lab-made
LiNi0.8Mn0.1Co0.1O2	197	2.7-4.5	77	0.1	0.1	4.4	200	63	0.185					11.00	80	10	10	10.1016/j.jechem.2019.12.009	polycrystalline, synthesis, lab-made
LiNi0.8Mn0.1Co0.1O2	228	2.7-4.5	85	0.1	0.1	4.4	200	81	0.095					2.00	80	10	10		polycrystalline, synthesis, lab-made
LiNi0.8Mn0.1Co0.1O2	228	2.7-4.5	80	0.1	0.1	4.4	200	66	0.170					5.00	80	10	10		polycrystalline, synthesis, lab-made
LiNi0.8Mn0.1Co0.1O2	213	2.7-4.5	90	0.1	1	4.5	200	61	0.195					2.5	80	10	10	10.1016/j.jechem.2022.03.053	polycrystalline, benchmark, lab-made
LiNi0.825Mn0.06Co0.115O2	200	2.7-4.3	87	0.09	2	4.3	200	83	0.085	187	178	166	140	1.61	80	10	10	10.1016/j.jechem.2022.04.037	polycrystalline, benchmark, lab-made
					5	4.3	200	51	0.245					1.61	80	10	10		
					1	4.25	500	77	0.046					1.61	80	10	10		

LiNi0.76Mn0.14Co0.10O2	200	2.7-4.5	85	0.1	0.33	4.5	200	80	0.100					4.94	80	10	10	10.1016/j.nanoen.2018.04.077	polycrystalline, synthesis, lab-made
LiNi0.76Mn0.14Co0.10O2	219	2.7-4.5	90	0.1	0.33	4.5	200	79	0.105					4.79	80	10	10		polycrystalline, synthesis, lab-made
LiNi0.76Mn0.14Co0.10O2	213	2.7-4.5	86	0.1	0.33	4.5	200	79	0.105					3.65	80	10	10		polycrystalline, synthesis, lab-made
LiNi0.76Mn0.14Co0.10O2	212	2.7-4.5	86	0.1	0.33	4.5	200	73	0.135					4.06	80	10	10		polycrystalline, synthesis, lab-made
LiNi0.76Mn0.14Co0.10O2	212	2.7-4.5	87	0.1	0.33	4.5	200	56	0.220					4.48	80	10	10		polycrystalline, synthesis, lab-made
LiNi0.76Mn0.14Co0.10O2	202	2.7-4.5	84	0.1	0.33	4.5	200	42	0.290					5.16	80	10	10		polycrystalline, synthesis, lab-made
LiNi0.8Mn0.1Co0.1O2	193	2.8-4.3	86	0.1	1	4.3	200	74	0.130	169	153	142	113		80	10	10	10.1016/j.nanoen.2020.105282	polycrystalline, benchmark, lab-made
					5	4.3	400	24	0.190						80	10	10		
					10	4.3	400	17	0.208						80	10	10		
LiNi0.8Mn0.1Co0.1O2	201	3.0-4.3	89	0.1	0.5	4.3	200	77	0.115					1.27	90	5	5	10.1016/j.nanoen.2021.106172	single crystal, benchmark, commercial
LiNi0.8Mn0.1Co0.1O2	208	2.7-4.3	87	0.2	1	4.3	200	72	0.140	199	192	184	168		70	20	10	10.1016/j.nanoen.2021.106301	polycrystalline, benchmark, commercial
LiNi0.8Mn0.1Co0.1O2	202	3.0-4.3	87	0.1	1	4.3	200	79	0.105	187	176	162			90	5	5	10.1016/j.nanoen.2021.106901	single crystal, benchmark, commercial
LiNi0.85Mn0.09Co0.06O2	203	2.7-4.3	85	0.1	1	4.3	90	81	0.211	179	170	158	142	0.75	80	10	10	10.1016/j.nanoen.2022.107626	polycrystalline, synthesis, lab-made
LiNi0.8Mn0.1Co0.1O2	179	2.8-4.3	87	0.1	0.1	4.3	100	87	0.130						80	10	10		polycrystalline, benchmark, lab-made
LiNi0.8Mn0.1Co0.1O2	195	2.8-4.5	85	0.1	0.1	4.5	100	75	0.250						80	10	10		polycrystalline, benchmark, lab-made
LiNi0.8Mn0.1Co0.1O2	218	2.75-4.5	89	0.1	1	4.5	500	4	0.192						80	10	10	10.1016/j.ensm.2021.06.018	polycrystalline, benchmark, lab-made
					1	4.3	100	83	0.170						80	10	10		
LiNi0.8Mn0.1Co0.1O2	177	3.0-4.2		0.5	0.5	4.2	570	80	0.035						96.3	1.2	2.5	10.1016/j.ensm.2021.11.017	polycrystalline, benchmark
					0.5	4.4	221	80	0.090						96.3	1.2	2.5		
LiNi0.83Mn0.05Co0.12O2	205	2.7-4.3	88	0.09	0.9	4.3	100	89	0.110	178	169	158	138	1.11	80	10	10	10.1016/j.gee.2022.09.003	single crystal, synthesis, lab-made
LiNi0.83Mn0.05Co0.12O2	195	2.7-4.3	82	0.09	0.9	4.3	100	86	0.140	178	169	156	135	1.77	80	10	10		single crystal, synthesis, lab-made
LiNi0.83Mn0.05Co0.12O2	190	2.7-4.3	81	0.09	0.9	4.3	100	85	0.150	178	169	151	125	2.34	80	10	10		single crystal, synthesis, lab-made
LiNi0.83Mn0.05Co0.12O2	205	2.7-4.3	88	0.09	0.9	4.3	100	72	0.280	169	160	144	113	2.73	80	10	10		single crystal, synthesis, lab-made
LiNi0.8Mn0.1Co0.1O2	202	2.8-4.5		0.19	0.5	4.5	200	69	0.155						80	10	10	10.1016/j.esci.2022.05.003	polycrystalline, benchmark
LiNi0.83Mn0.06Co0.11O2	194	2.8-4.3		0.1	1	4.3	100	67	0.330	174	160	143	122	2.36	80	10	10	10.1016/j.fmre.2022.03.001	polycrystalline, synthesis, lab-made
LiNi0.83Mn0.06Co0.11O2	199	2.8-4.3		0.1	1	4.3	100	74	0.260	184	178	168	149	2.74	80	10	10		polycrystalline, synthesis, lab-made
LiNi0.8Mn0.1Co0.1O2	194	2.8-4.3	86	0.2	0.5	4.3	200	67	0.165	172	160	151	130		80	10	10	10.1021/acs.inorgchem.9b02533	polycrystalline, benchmark, lab-made
LiNi0.8Mn0.1Co0.1O2	197	2.8-4.35		0.1	0.2	4.35	100	84	0.160	182	174	163	148	0.94	80	10	10	10.1021/acs.nanolett.2c01002	polycrystalline, benchmark, lab-made
					1	4.35	300	68	0.107					0.94	80	10	10		
LiNi0.8Mn0.1Co0.1O2	220	3.0-4.5	88	0.5	0.5	4.5	60	70	0.500						96	2	2	10.1021/acsaem.1c00130	polycrystalline, benchmark, lab-made
LiNi0.8Mn0.1Co0.1O2	191	3.0-4.3	85	1	1	4.3	120	71	0.242	195	185	175	153		80	10	10	10.1021/acsaem.1c00982	polycrystalline, benchmark, commercial
LiNi0.8Mn0.1Co0.1O2	191	3.0-4.3	86	0.1	1	4.3	800	47	0.066	188	170	150	125	2.53	80	10	10	10.1021/acsaem.1c01934	polycrystalline, benchmark, lab-made
LiNi0.8Mn0.1Co0.1O2	200	3.0-4.5		0.2	1	4.5	450	49	0.113					2.53	80	10	10		polycrystalline, benchmark, lab-made
LiNi0.8Mn0.1Co0.1O2	210	2.8-4.5	85	0.1	1	4.5	400	52	0.120					1.93	80	10	10	10.1021/acsaem.2c01812	single crystal, benchmark, lab-made
LiNi0.8Mn0.1Co0.1O2	173	2.8-4.3		0.08	0.8	4.3	500	50	0.100					4.00	80	10	10	10.1021/acsaem.2c02305	polycrystalline, benchmark, lab-made
LiNi0.83Mn0.06Co0.11O2	206	2.75-4.3	91	0.1	0.5	4.3	300	73	0.090	195	189		174		80	10	10	10.1021/acsaem.2c02746	polycrystalline, synthesis, lab-made
LiNi0.83Mn0.06Co0.11O2	203	2.75-4.3	91	0.1	0.5	4.3	300	62	0.127	189	182		167		80	10	10		polycrystalline, synthesis, lab-made
LiNi0.83Mn0.06Co0.11O2	213	2.75-4.3	92	0.1	0.5	4.3	300	80	0.067	198	192		180		80	10	10		polycrystalline, synthesis, lab-made
LiNi0.83Mn0.06Co0.11O2	205	2.75-4.3	90	0.1	0.5	4.3	300	92	0.027	193	188		177		80	10	10		polycrystalline, synthesis, lab-made
LiNi0.83Mn0.06Co0.11O2	203	2.75-4.3	87	0.1	0.5	4.3	300	93	0.023	190	186		175		80	10	10		polycrystalline, synthesis, lab-made
LiNi0.83Mn0.06Co0.11O2	201	2.75-4.3	87	0.1	0.5	4.3	300	94	0.020	190	184		172		80	10	10		polycrystalline, synthesis, lab-made
LiNi0.8Mn0.1Co0.1O2	209	3.0-4.4	93	0.1	0.5	4.4	200	74	0.130						80	10	10	10.1021/acsaem.9b00786	polycrystalline, benchmark, lab-made
LiNi0.8Mn0.1Co0.1O2	200	3.0-4.3	89	0.09	0.9	4.3	200	69	0.155						80	10	10	10.1021/acsaem.9b02486	polycrystalline, benchmark, commercial
LiNi0.8Mn0.1Co0.1O2	168	3.0-4.2	86	0.5	1	4.2	200	64	0.180						80	10	10	10.1021/acsami.0c05623	polycrystalline, benchmark, commercial
LiNi0.8Mn0.1Co0.1O2	193	3.0-4.35	84	0.5	1	4.35	200	57	0.215	193	190	175	152		80	10	10		polycrystalline, benchmark, commercial
LiNi0.8Mn0.1Co0.1O2	208	3.0-4.5	80	0.5	1	4.5	200	46	0.270						80	10	10		polycrystalline, benchmark, commercial
LiNi0.8Mn0.1Co0.1O2	199	3.0-4.2	90	0.1											95	2.5	2.5	10.1021/acsami.0c06830	polycrystalline, benchmark
LiNi0.83Mn0.05Co0.12O2	210	3.0-4.3	85	0.09	0.9	4.3	200	79	0.105						80	10	10	10.1021/acsami.1c00443	polycrystalline, benchmark, commercial
					0.9	4.4	200	66	0.170						80	10	10		
LiNi0.8Mn0.1Co0.1O2	167	2.7-4.3	75	1.1	1.1	4.3	400	90	0.025	175	166	150	121	3.41	70	20	10	10.1021/acsami.1c03195	polycrystalline, benchmark, lab-made
LiNi0.8Mn0.1Co0.1O2	199	2.7-4.5	80	1.1	1.1	4.5	400	72	0.070					3.41	70	20	10		polycrystalline, benchmark, lab-made
LiNi0.8Mn0.1Co0.1O2	238	2.7-4.7	68	1.1	1.1	4.7	400	60	0.100					3.41	70	20	10		polycrystalline, benchmark, lab-made
LiNi0.85Mn0.05Co0.1O2	209	3.0-4.3	91	0.1	1	4.3	120	80	0.167	193	186	179	157		94	3	3	10.1021/acsami.1c06839	polycrystalline, benchmark, lab-made
LiNi0.8Mn0.1Co0.1O2	206	3.0-4.3	91	0.09	0.27	4.3	100	74	0.260						90	5	5	10.1021/acsami.1c15271	polycrystalline, benchmark, commercial
LiNi0.8Mn0.1Co0.1O2	184	2.8-4.3	81	0.1	1	4.3	400	63	0.093	176	169	158	140		80	10	10	10.1021/acsami.1c17991	polycrystalline, benchmark, lab-made
LiNi0.83Mn0.06Co0.11O2	201	2.7-4.5		0.1	0.1	4.5	100	84	0.160						80	10	10	10.1021/acsami.2c09937	single crystal, benchmark, commercial
LiNi0.85Mn0.05Co0.1O2	215	2.8-4.3	79	0.1	3	4.3	100	68	0.320	184	162	139			80	10	10	10.1021/acsami.2c10613	polycrystalline, benchmark, commercial
LiNi0.75Mn0.11Co0.14O2	199	2.7-4.4	90	0.1	1	4.4	100	37	0.630					3.1	80	10	10	10.1021/acsami.2c20405	polycrystalline, synthesis, lab-made
LiNi0.8Mn0.1Co0.1O2	201	2.8-4.3	89	0.1	1	4.3	200	89	0.055	185	178	170	154	2.05	80	10	10	10.1021/acsami.3c00607	polycrystalline, synthesis, lab-made
LiNi0.8Mn0.1Co0.1O2	203	2.8-4.3	89	0.1	1	4.3	100	88	0.120	186	180	172	159	3.48	80	10	10		polycrystalline, synthesis, lab-made
LiNi0.8Mn0.1Co0.1O2	232	2.0-4.8	85	0.1	0.5	4.8	200	40	0.300						80	10	10	10.1021/acsami.5b09938	polycrystalline, benchmark, lab-made
LiNi0.76Mn0.14Co0.1O2	195	2.7-4.5	82	0.33	0.33	4.5	200	68	0.160						80	10	10	10.1021/acsami.6b06172	polycrystalline, benchmark, lab-made
LiNi0.8Mn0.1Co0.1O2	219	2.7-4.4	88	0.1	0.5	4.4	100	80	0.200						85	10	5	10.1021/acsami.6b09197	polycrystalline, benchmark, lab-made
					1	4.4	100	81	0.190						85	10	5		
LiNi0.8Mn0.1Co0.1O2	184	2.5-4.5		1	1	4.5	50	92	0.160						90	5	5	10.1021/acsami.8b06399	polycrystalline, synthesis, lab-made
LiNi0.8Mn0.1Co0.1O2	190	2.8-4.3	80	0.09	0.9	4.3	100	84	0.160	156	150	142	124		75	15	10	10.1021/acsami.9b10310	polycrystalline, benchmark, lab-made
LiNi0.8Mn0.1Co0.1O2	196	3.0-4.3	81	0.085	0.85	4.3	100	70	0.300	193	183	174	150	3.06	90	5	5	10.1021/acsami.9b18946	polycrystalline, benchmark, lab-made
					4.25	4.3	300	26	0.247					3.06	90	5	5		
LiNi0.8Mn0.1Co0.1O2	216	3.0-4.5	88	0.085	0.85	4.5	100	74	0.260					3.06	90	5	5		polycrystalline, benchmark, lab-made
LiNi0.8Mn0.1Co0.1O2	200	2.8-4.3	84	0.1	1	4.3	200	80	0.100	192	177	174	160		85	10	5	10.1021/acsenergylett.0c01032	single crystal, benchmark, commercial
LiNi0.8Mn0.1Co0.1O2	203	2.8-4.5	83	0.1	1	4.5	100	74	0.260						85	10	5		single crystal, benchmark, commercial
LiNi0.8Mn0.1Co0.1O2	160	2.6-4.2	83	1	1	4.2	225	76	0.107						80	10	10	10.1021/acsomega.2c03074	polycrystalline, benchmark, commercial
LiNi0.8Mn0.1Co0.1O2	215<br>194	2.8-4.5	90	0.1<br>0.2	0.1	4.5	100	72	0.280						80	10	10	10.1021/acssuschemeng.0c00011	polycrystalline, benchmark, lab-made
					0.2	4.5	100	79	0.210						80	10	10		
					0.5	4.5	100	75	0.250						80	10	10		
					1	4.5	200	64	0.180						80	10	10		
					2	4.5	450	19	0.180						80	10	10		
LiNi0.8Mn0.1Co0.1O2	186	2.5-4.25	95	0.2	0.2	4.25	100	52	0.480						96	2	2	10.1021/acssuschemeng.0c00404	polycrystalline, benchmark, commercial
LiNi0.8Mn0.1Co0.1O2	198	3.0-4.3	88	0.1	1	4.3	100	79	0.210	185	179		131	2.97	92.5	5	2.5	10.1021/acssuschemeng.1c04076	polycrystalline, benchmark, lab-made
					1	4.5	100	56	0.440					2.97	92.5	5	2.5		
LiNi0.83Mn0.05Co0.12O2	192	3.0-4.3	87	0.09	0.27	4.3	100	87	0.130						90	5	5	10.1021/acssuschemeng.2c06720	polycrystalline, benchmark, commercial

LiNi0.8Mn0.1Co0.1O2	207	3.0-4.3	87	0.09	0.5	4.3	100	62	0.380	190	182	174	161	1.8	85	10	5	10.1557/adv.2020.90	polycrystalline, benchmark, lab-made
LiNi0.8Mn0.1Co0.1O2	195	3.0-4.3	86	0.1	1	4.3	300	85	0.050	184	175	161	127		80	10	10	10.1002/aenm.201803963	polycrystalline, benchmark, lab-made
LiNi0.8Mn0.1Co0.1O2	198	3.0-4.3	81	0.1	1	4.3	300	96	0.013	189	182	172	153		80	10	10		polycrystalline, synthesis, lab-made
LiNi0.8Mn0.1Co0.1O2	200	3.0-4.3	89	0.0925	0.46	4.3	200	72	0.140	170	138	102			94	4	2	10.1002/aenm.202001069	polycrystalline, benchmark, commercial
LiNi0.8Mn0.1Co0.1O2	203	2.8-4.4	87	0.1	0.33	4.4	100	91	0.090					2.2	90	5	5	10.1002/adma.202100804	polycrystalline, synthesis, lab-made
LiNi0.8Mn0.1Co0.1O2	230	2.5-4.5	84	0.1	0.5	4.5	300	84	0.053					6.75	80	10	10	10.1002/advs.201600262	polycrystalline, synthesis, lab-made
LiNi0.8Mn0.1Co0.1O2	192	3.0-4.3	85	0.2	1	4.3	100	76	0.240	185	180	174			92.5	4.5	3	10.1002/advs.202001809	polycrystalline, benchmark, lab-made
					0.5	4.3	300	77	0.077						92.5	4.5	3		
LiNi0.85Mn0.05Co0.1O2	209	3.0-4.3	91	0.1	1	4.3	120	80	0.167	193	186	178	157		94	3	3	10.1002/batt.202000191	polycrystalline, benchmark, lab-made
					1	4.3	500	52	0.096						94	3	3		
LiNi0.85Mn0.05Co0.1O2	198	3.0-4.3	88	0.1	1	4.3	50	91	0.180						96.5	1.5	2	10.1002/batt.202200466	polycrystalline, benchmark, lab-made
LiNi0.85Mn0.05Co0.1O2	203	3.0-4.3	90	0.1	1	4.3	50	84	0.320						96.5	1.5	2		polycrystalline, benchmark, lab-made
LiNi0.8Mn0.1Co0.1O2	203	3.0-4.3	96	0.1	0.1	4.3	100	84	0.160						92	4	4	10.1002/bkcs.12118	polycrystalline, benchmark, commercial
LiNi0.8Mn0.1Co0.1O2	200	3.0-4.5		0.1	5	4.5	500	70	0.060					2.4	80	10	10	10.1039/c9ta13014d	polycrystalline, benchmark, lab-made
LiNi0.8Mn0.1Co0.1O2	182	3.0-4.3	82	0.1	0.1	4.3	50	81	0.380						80	10	10	10.1149/2.0291902jes	polycrystalline
LiNi0.8Mn0.1Co0.1O2	195	3.0-4.3	90	0.1	0.1	4.3	50	71	0.580	178	156	120			80	10	10		polycrystalline
LiNi0.8Mn0.1Co0.1O2	200	3.0-4.3	91	0.1	0.1	4.3	50	72	0.560	183	170	145			80	10	10		polycrystalline
LiNi0.8Mn0.1Co0.1O2	202	3.0-4.3	92	0.1	0.1	4.3	50	93	0.140	184	174	153			80	10	10		polycrystalline
LiNi0.8Mn0.1Co0.1O2	189	2.75-4.3	93	0.2	0.2	4.3	50	95	0.100	177	163	150	144		80	10	10	10.1002/celc.201900511	polycrystalline, synthesis, lab-made
					1	4.3	100	82	0.180						80	10	10		
LiNi0.8Mn0.1Co0.1O2	196	2.75-4.3	84	0.2	0.2	4.3	100	86	0.140	182	176	169	159	3.59	80	10	10	10.1002/celc.202100693	polycrystalline, benchmark, lab-made
					1	4.3	200	64	0.180					3.59	80	10	10		
LiNi0.85Mn0.05Co0.1O2	194	2.8-4.3	80	0.1	0.5	4.3	100	71	0.290	178	165	145	123		70	20	10	10.1002/cnma.202100168	polycrystalline, benchmark, lab-made
LiNi0.8Mn0.1Co0.1O2	181	3.0-4.3	91	0.1	1	4.3	100	38	0.620						90	5	5	10.1002/cphc.201700921	polycrystalline, benchmark, commercial
LiNi0.76Mn0.14Co0.10O2	215	2.7–4.5	88	0.1	0.3	4.5	200	85	0.075						80	10	10	10.1002/cssc.201800706	polycrystalline, benchmark, lab-made
					1	4.5	200	73	0.135						80	10	10		
					2	4.5	200	58	0.210						80	10	10		
					5	4.5	200	53	0.235						80	10	10		
LiNi0.83Mn0.05Co0.12O2	215	2.75-4.3	89	0.1	0.2	4.3	100	90	0.100	196	188	178	160		90	4	6	10.1039/d0nj05914e	polycrystalline, synthesis, lab-made
LiNi0.83Mn0.05Co0.12O2	210	2.75-4.3	91	0.1	0.2	4.3	100	100	0.000	188	184	178	167		90	4	6		single crystal, synthesis, lab-made
LiNi0.83Mn0.05Co0.12O2	210	2.7–4.5	95	0.2	1	4.5	200	52	0.240						70	20	10	10.1039/D2TA02472A	polycrystalline, benchmark, lab-made
LiNi0.80Mn0.05Co0.15O2	208	2.8–4.35	93	0.1	1	4.35	50	92	0.160						92	4	4	10.1002/ente.201700950	polycrystalline, benchmark, lab-made
LiNi0.8Mn0.1Co0.1O2	194	2.7–4.3	86	0.2	1	4.3	50	95	0.100	182	167	145			80	10	10	10.1002/ente.201800415	polycrystalline, synthesis, lab-made
LiNi0.82Mn0.09Co0.09O2	201	2.5-4.25	86	0.2						192	170		12	1.29	96	2	2	https://doi/org/10.1002/ente.202000800	polycrystalline, benchmark, commercial
LiNi0.8Mn0.1Co0.1O2	201	2.75–4.3		0.1	0.5	4.3	200	72	0.140	173	158	132	91		90	5	5	10.1002/ente.202101013	polycrystalline, benchmark, commercial
LiNi0.8Mn0.1Co0.1O2	190	2.8-4.3	87	0.2	1	4.3	100	93	0.070	183	167	146			71.4	14.3	14.3	10.5229/JECST.2019.10.2.196	polycrystalline, benchmark, commercial
					5	4.3	500	47	0.106						71.4	14.3	14.3		
LiNi0.75Mn0.13Co0.12O2	199	2.5-4.3	80	0.1	1	4.3	100	75	0.250	187	180	167	158		80	10	10	10.30919/es8d502	polycrystalline, benchmark, lab-made
LiNi0.8Mn0.1Co0.1O2	202	2.7–4.3	81	0.09	0.9	4.3	200	76	0.120	192	184		142		80	10	10	10.3389/fchem.2019.00500	polycrystalline, benchmark, lab-made
LiNi0.8Mn0.1Co0.1O2	198	3.0-4.3	88	0.2	1	4.3	100	72	0.280	189	183	178			80	10	10	10.1149/2.0331908jes	polycrystalline, benchmark, lab-made
LiNi0.83Mn0.07Co0.10O2	220	3.0-4.3	87	0.1	1	4.3	100	57	0.430	207	193	187			90	5	5	10.1002/er.8511	polycrystalline, benchmark, commercial
LiNi0.8Mn0.1Co0.1O2	198	3.0-4.3	89	0.1	1	4.3	200	50	0.250	175	158	129	62		94	3	3	https://doi/org/10.1002/er.8227	polycrystalline, benchmark, lab-made
LiNi0.8Mn0.1Co0.1O2	194	3.0-4.25	85	0.09	1	4.25	50	76	0.480						96	2	2	10.33961/jecst.2020.00829	polycrystalline, benchmark
LiNi0.85Mn0.05Co0.1O2	210	3.0-4.3	91	0.1	1	4.3	120	80	0.167						94	3	3	10.1149/1945-7111/ac0ab0	polycrystalline, benchmark, lab-made
LiNi0.83Mn0.05Co0.12O2	188	2.7–4.5	81	0.1	0.1	4.5	50	68	0.640						80	10	10	10.3740/MRSK.2018.28.5.273	polycrystalline, benchmark, lab-made
LiNi0.8Mn0.1Co0.1O2	200	2.7–4.3	85	0.1						188	175		127		80	10	10	10.3740/MRSK.2020.30.11.636	polycrystalline, synthesis, lab-made
LiNi0.8Mn0.1Co0.1O2	218	2.7–4.3	90	0.1	0.6	4.3	90	73	0.300	202	193	180	168	2.91	80	10	10	10.1007/s10008-017-3564-9	polycrystalline, benchmark, lab-made
LiNi0.8Mn0.05Co0.15O2	196	2.8-4.3	88	0.1	0.1	4.3	200	87	0.065	181	171	136	100		80	10	10	10.1007/s10008-018-4130-9	polycrystalline, benchmark, lab-made
LiNi0.82Mn0.06Co0.12O2	210	2.8-4.3	88	0.1	1	4.3	100	86	0.140	187	180	172	161		80	10	10	10.1007/s11581-021-04171-1	polycrystalline, benchmark, commercial
LiNi0.8Mn0.1Co0.1O2	194	2.8-4.3	84	0.1	1	4.3	200	76	0.120	190	182	172	147		80	10	10	10.1007/s40843-017-9162-3	polycrystalline, benchmark, lab-made
LiNi0.84Mn0.05Co0.11O2	195	3.0-4.3	85	0.1	0.5	4.3	80	47	0.663	170	153	123	25		96	2	2	10.1038/s41598-020-64546-8	polycrystalline, benchmark, lab-made
					0.5	4.5	80	42	0.725						96	2	2		
LiNi0.8Mn0.1Co0.1O2	201	2.8-4.5	83	0.1	1	4.5	200	52	0.240						80	10	10	10.1142/S1793292021500417	polycrystalline, benchmark, commercial
LiNi0.8Mn0.1Co0.1O2	201	3.0-4.3	92	0.1	0.5	4.3	50	88	0.240	185	175	160			80	10	10	10.1002/smll.202107346	polycrystalline, synthesis, lab-made
LiNi0.8Mn0.1Co0.1O2	215	2.7–4.3	96	0.1	0.5	4.3	100	94	0.060						90	5.5	4.5	10.1002/smll.201803179	polycrystalline, synthesis, lab-made
LiNi0.8Mn0.1Co0.1O2	196	2.8-4.35		0.19	0.19	4.3	50	95	0.100						80	10	10	10.1088/1757-899X/301/1/012039	polycrystalline, benchmark, lab-made
					0.95	4.3	50	92	0.160						80	10	10		
LiNi0.8Mn0.1Co0.1O2	191	2.8-4.3	91	0.1	0.1	4.3	500	43	0.114						80	10	10	10.1149/2.0841613jes	polycrystalline, benchmark, lab-made
LiNi0.83Mn0.06Co0.11O2	210	2.7–4.3	85	0.1	1	4.3	100	88	0.120	198	190	177	135		70	20	10	10.1016/j.apsusc.2022.154101	polycrystalline, benchmark, lab-made
