# Supplementary material for: Benchmarking the electrochemical parameters of the LiNi0.8Mn0.1Co0.1O2 positive electrode material for Li-ion batteries
Source: Heliyon. 2023 Nov 1;9(12):e21881. doi: 10.1016/j.heliyon.2023.e21881 (PMC10709181; doi:10.1016/j.heliyon.2023.e21881)
Supplement: Multimedia component 2 [file mmc2.docx]

**Supporting information.**

**Benchmarking the electrochemical parameters of the LiNi_0.8_Mn_0.1_Co_0.1_O_2_ positive electrode material for Li-ion batteries**

Aleksandra A. Savina and Artem M. Abakumov*

Center for Energy Science and Technology, Skolkovo Institute of Science and Technology, Bolshoy Boulevard 30, bld. 1, 121205 Moscow

Corresponding Author: [*a.abakumov@skoltech.ru](mailto:*a.abakumov@skoltech.ru)


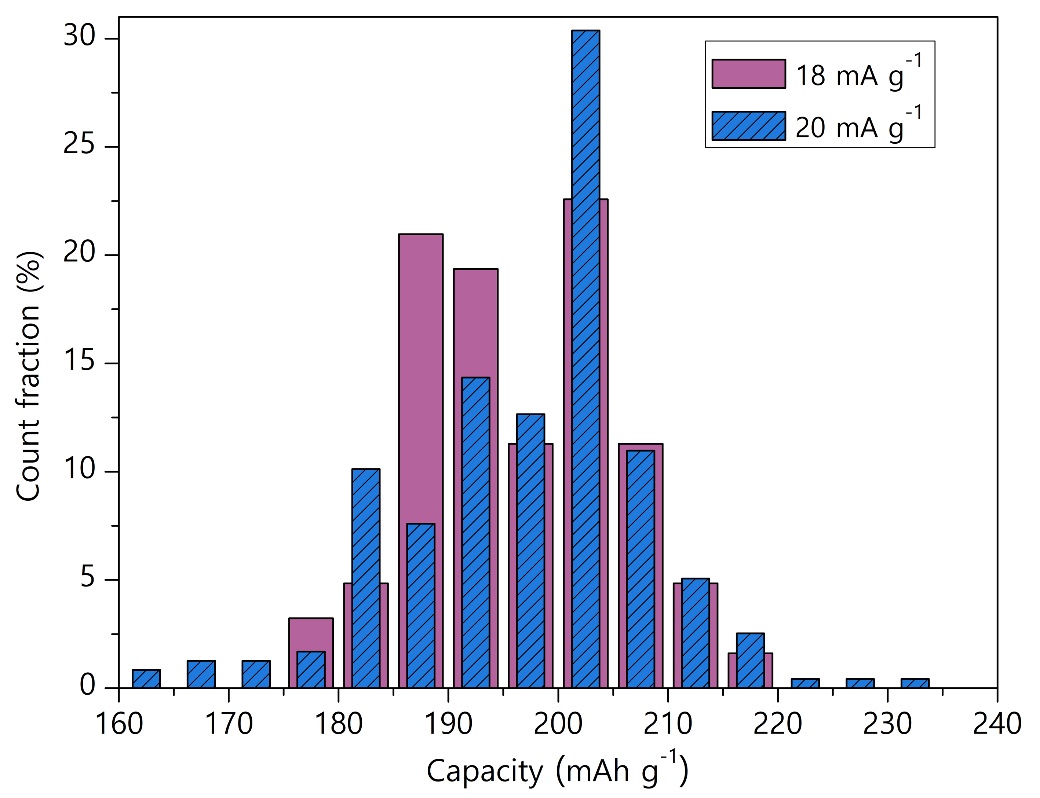


Figure S1. Distribution histograms of first discharge capacities measured at E_U_ = 4.3 V vs Li/Li^+^ and current densities of 20 and 18 mA/g (C-rates of 0.1C and 0.09C, respectively). The distributions are nearly bimodal, with the first broad peak centered at ~192-195 mAh/g and the second narrow peak at ~202-203 mAh/g. Although two distributions differ in the relative weights of these two peaks, the peak centers are nearly the same taking into account the large spread of the observed capacities. This indicates that the discharge capacity is not significantly affected by the 0.01C difference in the C-rate.


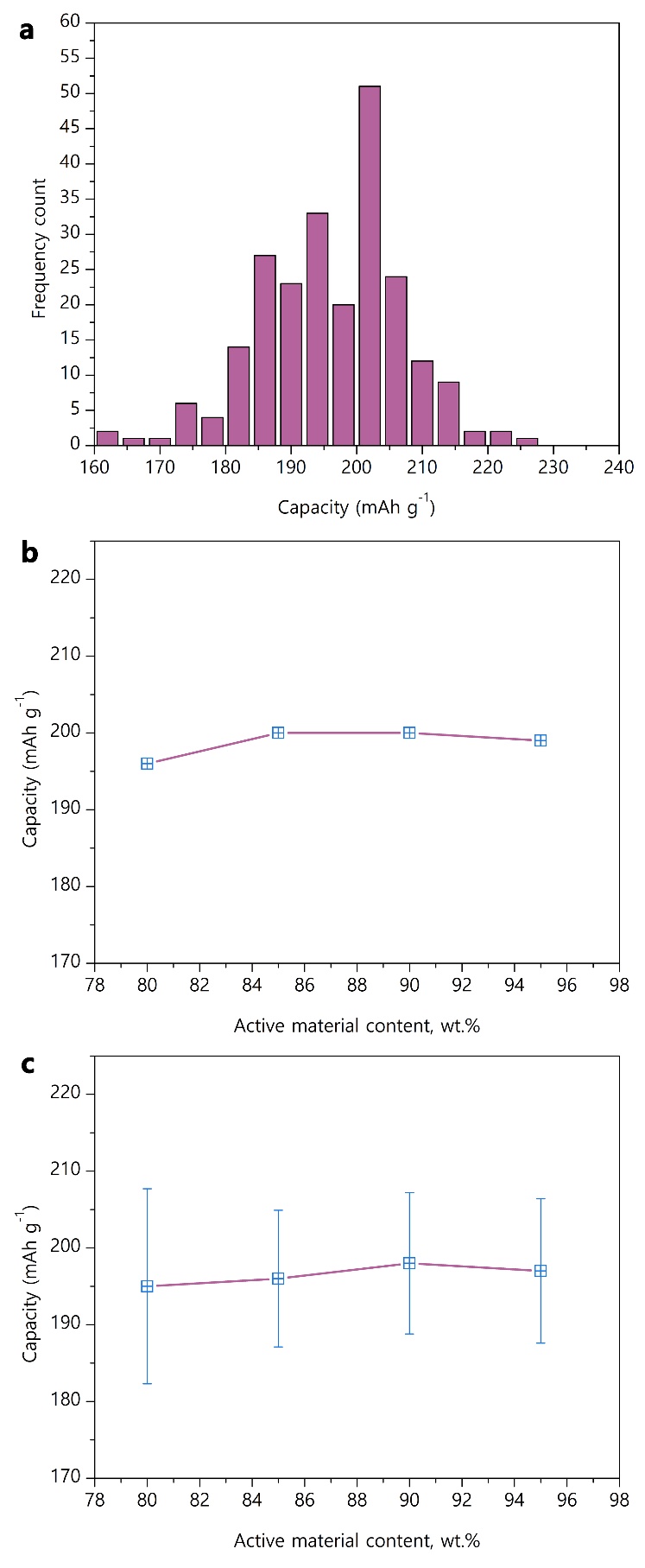


Figure S2. (a) Distribution histogram of first discharge capacity at *E*_U_ = 4.3 V, C-rate of 0.1C at the 80:10:10 wt.% ratio of active material to conductive additive to binder in the electrode composition. Dependence of the median (b) and mean (with standard deviation) values of first discharge capacity on the active material content in the electrodes.


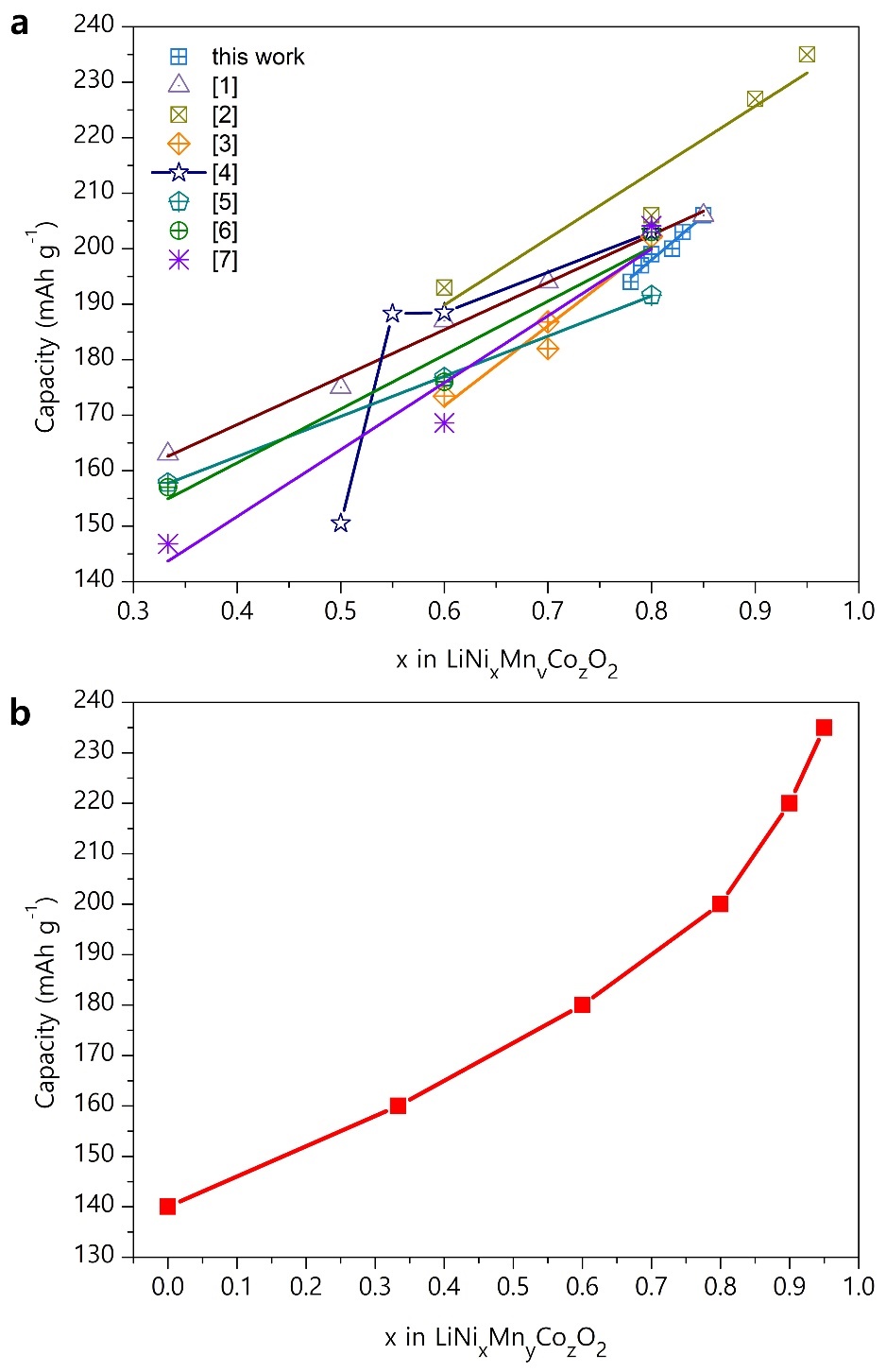


Figure S3. (a) Dependence of the first discharge capacity of the LiNi_x_Mn_y_Co_z_O_2_ layered oxides on the Ni content along with the literature data (E_U_ = 4.3 V vs Li/Li^+^, C-rate 0.1C) [1-7]. (b) Tentative C-x dependence according to the commonly accepted discharge capacity values (LiCoO_2_: 140 mAh/g, NMC111: 160 mAh/g, NMC622: 180 mAh/g, NMC811: 200 mAh/g, NMC9 0.5 0.5: 220 mAh/g, NMC9.5 0.25 0.25: 235 mAh/g.


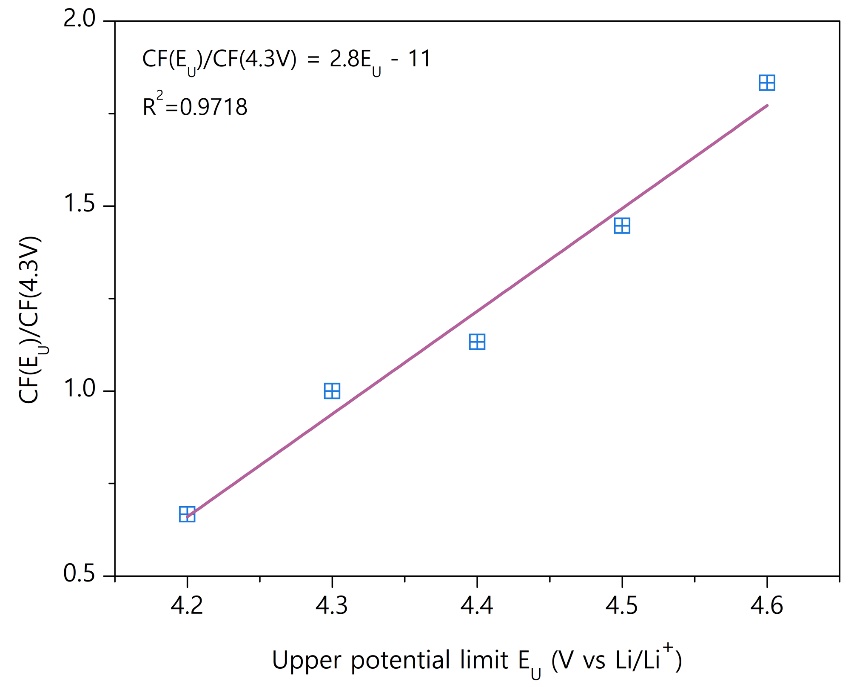


Figure S4. Capacity fade at E_U_ relative to the capacity fade at E_U_ = 4.3V at the C-rate of 1 C. The linear fit corresponds to the slope of 2.78±0.23 and intercept of -11±1.0.


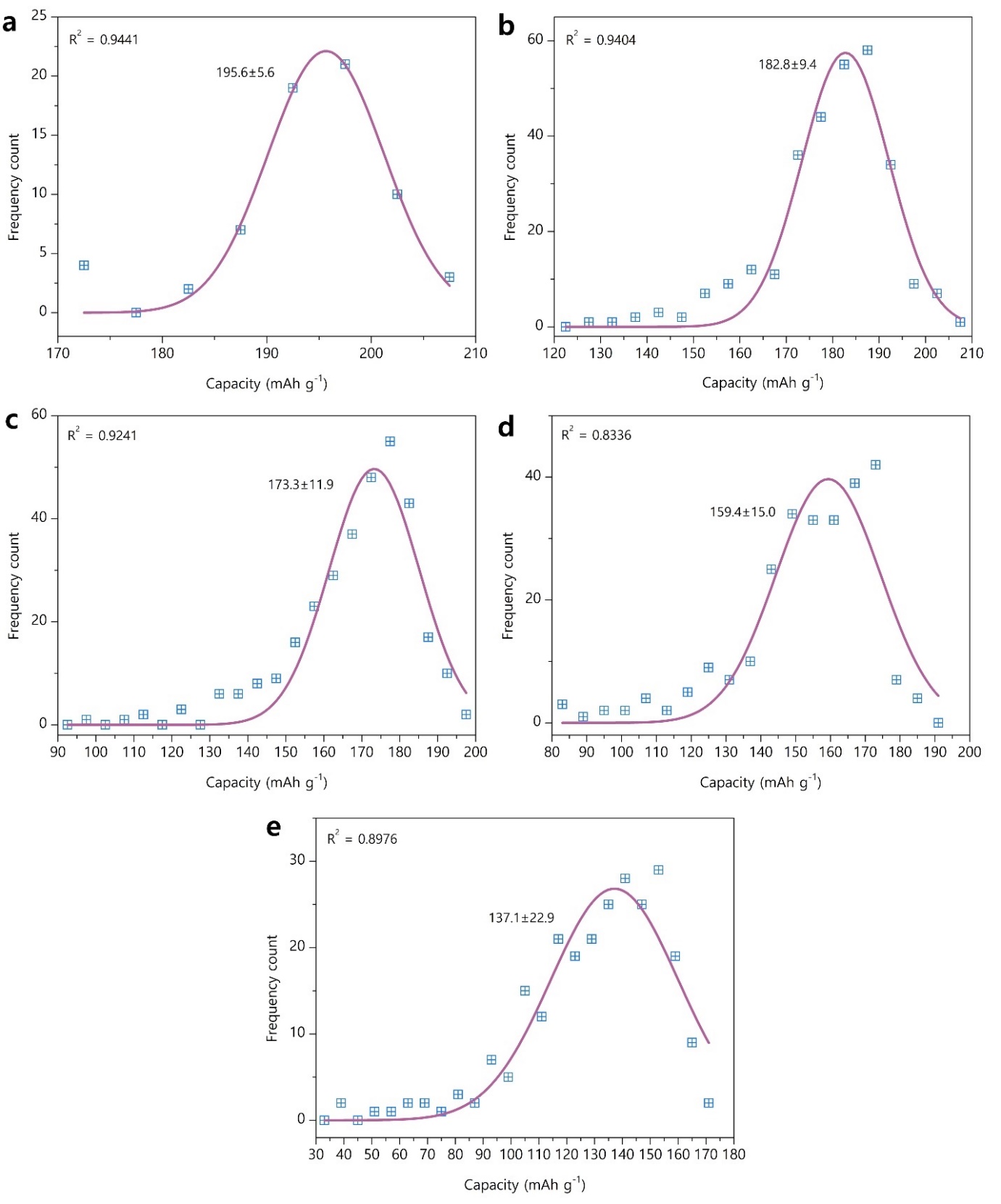


Figure S5. Discharge capacity at E_U_ = 4.3V vs Li/Li^+^ at the current rate of 0.2C (a), 0.5C (b), 1C (c), 2C (d) and 5C (e) fitted with the Gaussian functions. The mean values and standard deviations are marked. Adjusted R^2^ coefficients are provided as a goodness of fit measures.

**Synthesis and characterization of NMC811.**

In order to corroborate the parameters retrieved from the analysis of the published data, NMC811 cathode material has been prepared via a conventional hydroxide co-precipitation method followed by high temperature lithiation. The synthesis of a mixed transition metal (TM) hydroxide precursor was performed in a 5L continuously stirred batch reactor under Ar atmosphere. The reactor was filled with 1.25L of 2 mol/L NH_3_·H_2_O (SigmaTec) and preheated to 50°C. The aqueous solution of Ni^2+^, Mn^2+^ and Co^2+^ sulfates (Alfa Aesar, ≥99%) taken in the 0.8:0.1:0.1 molar ratio and total concentration of 2 mol/L was poured into the reactor together with the 4 mol/L solution of NaOH (Alfa Aesar, ≥99%) and 4 mol/L solution of NH_3_∙H_2_O as the precipitating and complexing agents, respectively, at the constant rate of 0.3 L/h keeping the continuous stirring at 1000 rpm. The temperature of the reactor and pH value were fixed to 50°C and 11.2-11.3, respectively. After finalizing the co-precipitation step, the reaction mixture was kept at the same conditions for 20 hours under stirring for aging. The obtained mixed precursor was filtered, washed with distilled water, and dried at 90°C for 12 h under dynamic vacuum. In order to obtain the final cathode material, the precursor was mixed with LiOH∙H_2_O (Alfa Aesar, ≥99.9%) in a molar ratio of 1:1.05, and annealed at 750°С for 12 h in flowing oxygen.

Powder X-ray diffraction (PXRD) data were collected using a Huber G670 Guinier diffractometer (CoK$\alpha$_1_ radiation $(\lambda$=1.78892 $Å$), Ge (111) monochromator, image plate detector). The crystal structure of NMC811 was refined using the Rietveld method according to the following scheme. The 3*a* (0,0,0) site was jointly occupied by Li and Ni, while Li, Ni, Mn, Co were placed at the 3*b* (0,0, 1⁄2) site of the *R*-3*m* α-NaFeO_2_-type structure. The sum of occupancy factors gLi(3*a*) and gNi(3*a*) was fixed to 1, whereas the occupancy factors gMn(3*b*) and gCo(3*b*) were fixed to 0.1 according to the nominal chemical composition, and the sum gLi(3*b*) + gNi(3*b*) was set to 0.8. Overall atomic displacement parameter (ADP) and March-Dollase preferred orientation parameter along [001] direction were also refined [8]. The JANA2006 [9] program package was used for data processing.

The specific surface area (SSA) of the sample was determined using nitrogen physisorption method (NovaTouch, Quantachrome, USA). Before the experiments, each sample was degassed under vacuum at 200 °C for 8 h. The specific surface area was calculated with the Brunauer–Emmett–Teller (BET) method.

Tap density of the material was determined with a tapped density analyzer (Quantachrome Autotap, USA). At least 50 g of Ni-rich NMC powder was poured into 50 ml glass cylinder and the cylinder was tapped 3000 times before measuring the sample volume. Before the measurements, sample was dried under vacuum at 110 °C for 2-5 h.

Particle size and morphology were investigated by scanning electron microscopy (SEM) using a ThermoFisher Quattro S microscope.

High angle annular dark field scanning transmission electron microscopy (HAADF-STEM) images and energy-dispersive X-ray spectra in the STEM mode (STEM-EDS) were acquired using a ThermoFisher Titan Themis Z transmission electron microscope at 200 kV supplied with Super-X EDS system. The samples were prepared in air by crushing the crystals with an agate mortar and pestle in ethanol and dropping the suspension onto a carbon film supported by a copper grid.

In order to investigate electrochemical properties of NMC811, galvanostatic charge/discharge cycling was carried out at 25°C in the different potential windows (from 2.7 V to 4.2-4.8 V vs. Li/Li^+^) and at different current densities from 0.1C to 5C (1C = 200 mA/g) with a Neware BTS-4008 battery tester under control of BTSDA software. The electrode composition was prepared by mixing 80 wt. % of the active material, 10 wt. % of carbon Super-P and 10 wt. % of polyvinylidene fluoride (PVDF) in N-methylpyrrolidone (NMP). The resulting homogeneous slurry was deposited onto a carbon-coated Al current collector using an automatic film applicator Zehntner ZAA 2300 with 150 µm layer thickness and dried at 70°C until complete evaporation of NMP. The resulting electrode tape was compacted on steel rolls, punched into round discs with an area of ~2 cm^2^ (d = 16 mm) and active mass loading of ~3-4 mg/cm^2^, and dried under vacuum at 110°C for 12 hours. Coin-type half-cells (2032 R) were assembled in an Ar-filled glove box using 1M LiPF_6_ (Sigma-Aldrich, $\geq$99.99%) solution in the ethylene carbonate/propylene carbonate/dimethyl carbonate mixture (EC:PC:DMC=1:1:3 vol. ratio) as electrolyte.

The PXRD pattern of the NMC811 sample demonstrates a layered α-NaFeO_2_-type structure (sp. gr. *R*$\bar{3}$*m*) without impurity phases (Figure S5). The lattice parameters, as well as the amount of Ni^2+^/Li^+^ disorder, originated from the partial interchange of the Li^+^ (r = 0.76 Å) and Ni^2+^(r = 0.69 Å) cations between the 3*b* and 3*a* sites calculated with the Rietveld refinement are shown in Table S1. The material yields the expected morphology of Ni-rich NMCs obtained by the co-precipitation method and consists of roundish agglomerates of ~10-12 µm in size (Figure S6). Each secondary particle consists of primary particles with a size of 50-100 nm. The tap density of NMC811 is found to be 2.5 g/cm^3^, the specific surface area is 0.6 m^2^/g. STEM-EDS analysis proves that the cation composition of the NMC811 sample corresponds to the target stoichiometry (molar Ni:Mn:Co ratio is 80.1(9):9.8(7):10.1(2)). TM elements are distributed uniformly throughout the sample (Figure S7).

Figure S9 exhibits galvanostatic charge-discharge curves at different current densities increasing from 0.1C to 0.2C, 0.5C, 1C, 2C and 5C. The corresponding discharge capacities reach 200, 192, 179, 165, 157 and 132 mAh/g, respectively (Figure S9). After 100 and 200 cycles, the reversible discharge capacity at 1C (165 mAh/g) is maintained at about 150 and 128 mAh/g, respectively (Figure S10). Besides, the NMC811 cathode was cycled in half-cell at a rate of 0.1 C with an upper cutoff voltage ranging from 4.2 to 4.8 V (Figure S11). At 4.2 V, the discharge capacity is 180 mAh/g, which progressively increases with the cutoff voltage to 205 mAh/g for 4.4 V, 210 mAh/g for 4.5 V, 214 mAh/g for 4.6 V, 217 mAh/g for 4.7 V and 221 mAh/g for 4.8 V.


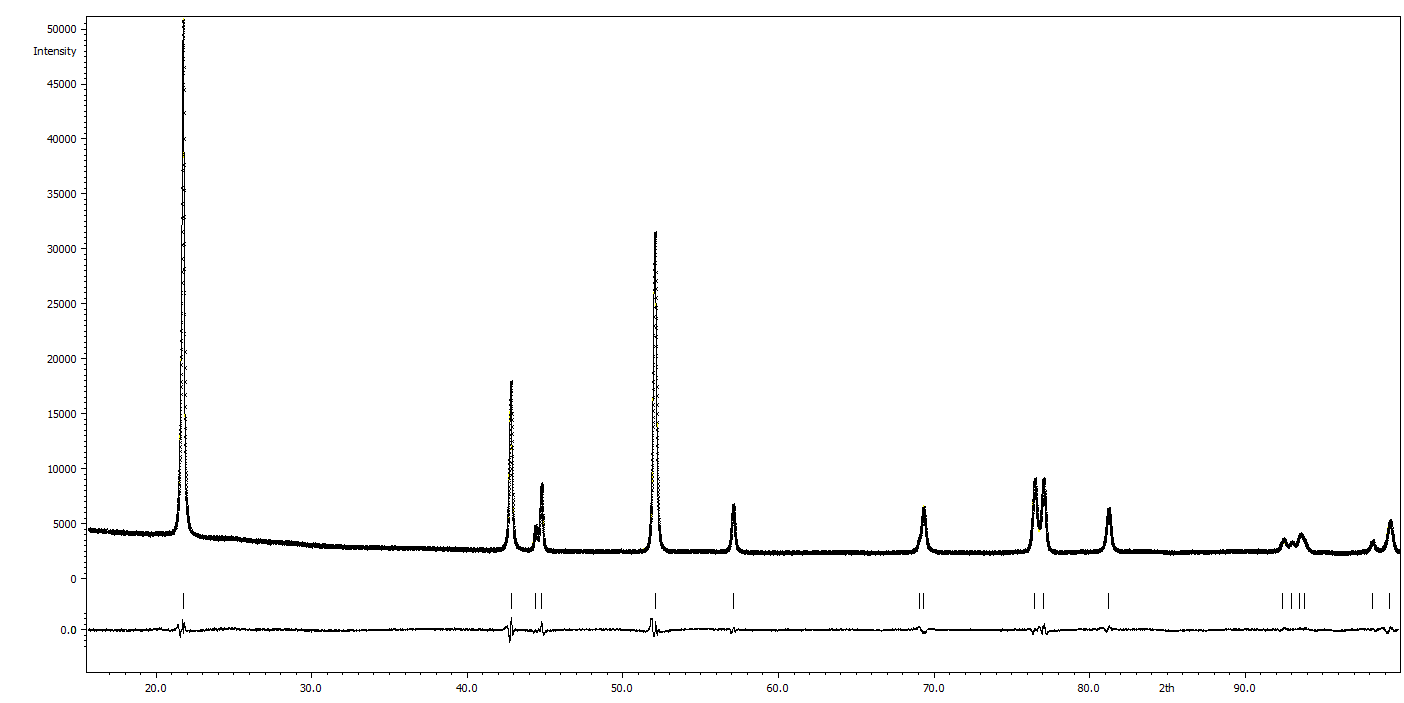


Figure S6. Experimental, calculated and difference PXRD patterns after Rietveld refinement for NMC811. Theoretical Bragg reflection positions for the NMC811 structure (sp. gr. *R*$\bar{3}$*m*) are designated with vertical lines.

Table S1. Crystallographic data after the Rietveld refinement for the LiNi_0.8_Mn_0.1_Co_0.1_O_2_ sample.

| Refined composition | Li_0.998_Ni_0.802_Mn_0.1_Co_0.1_O_2_ |
| --- | --- |
| Space group | *R*$\bar{3}$*m* |
| *a*, Å | 2.87214(1) |
| *c*, Å | 14.2023(1) |
| V, Å^3^ | 101.461(1) |
| Ni^2+^ in 3*a* site, % | 2.84(5) |
| Li^+^ in 3*b* site, % | 2.67(1) |
| z_O_ | 0.24137(6) |
| U_iso_, Å^2^ | 0.0133(1) |
| March-Dollase preferred orientation parameter, τ | 0.9818(6) |
| d(Li-O), Å | 2.1108(4) |
| d(TM-O), Å | 1.9686(4) |
| R_F_; R_p_, % | 2.09; 1.28 |


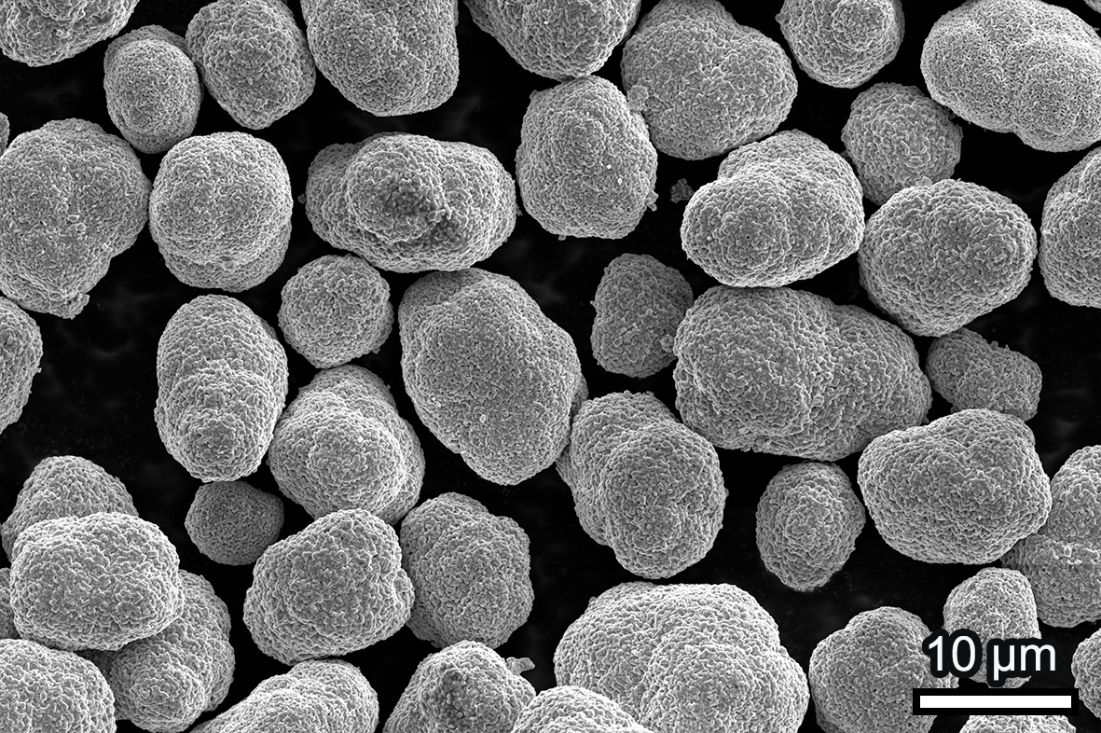

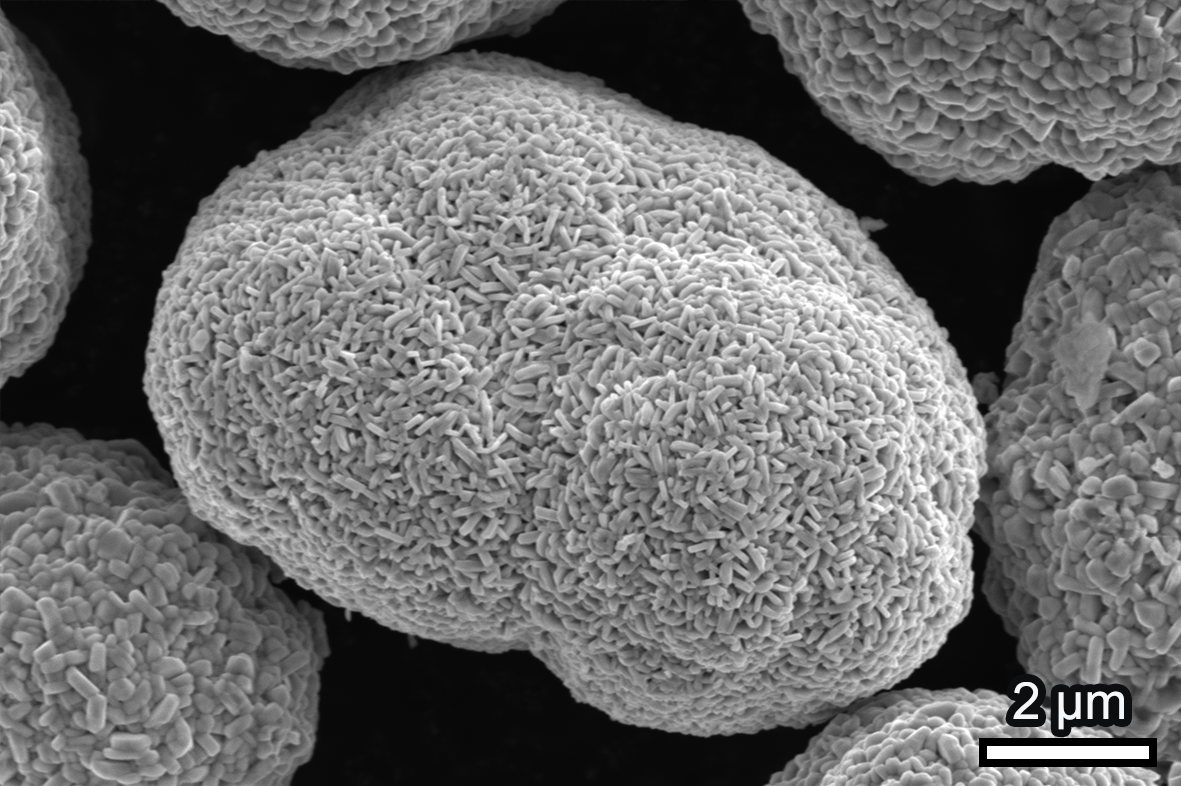


Figure S7. SEM images of NMC811 at different magnifications.


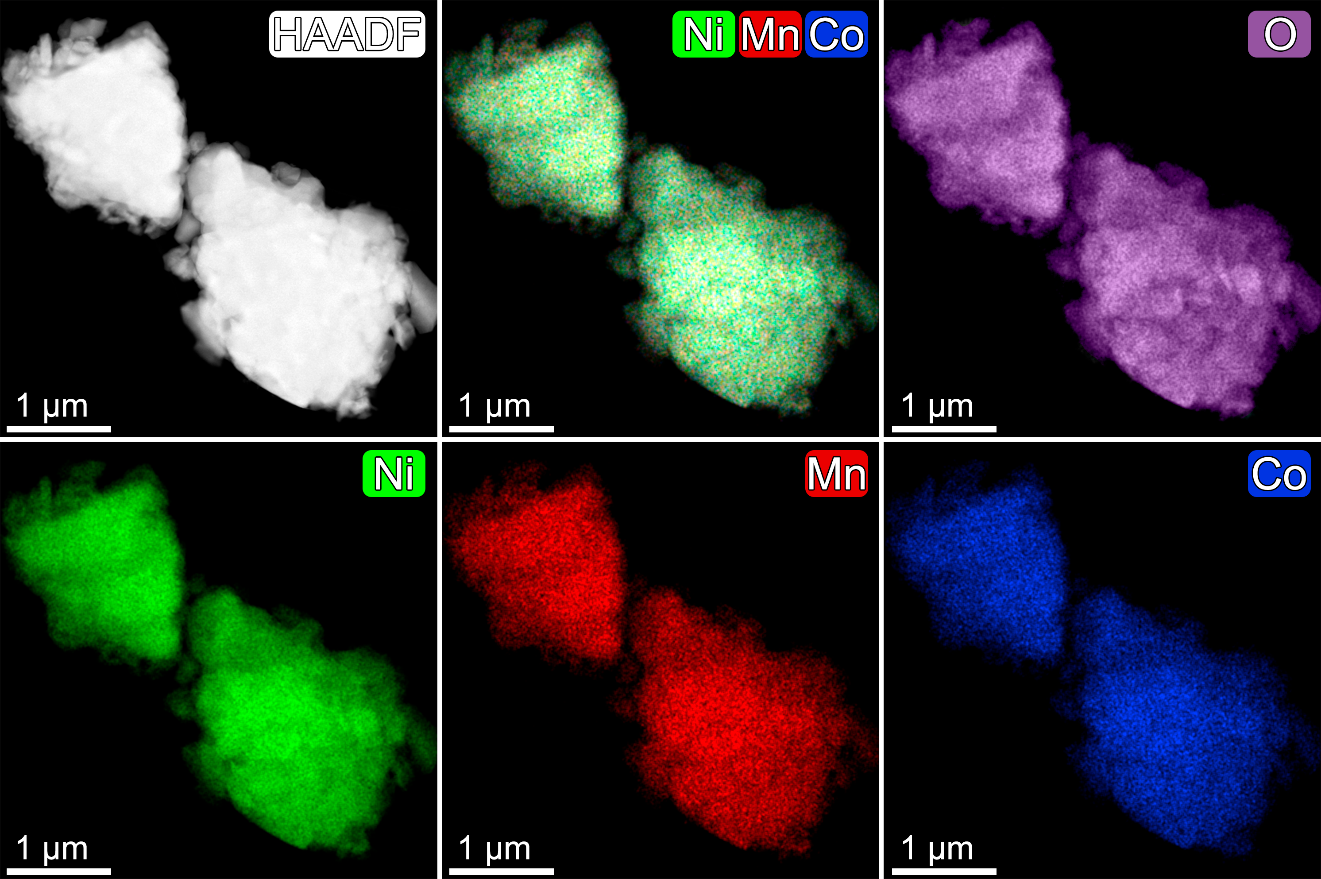


Figure S8. HAADF-STEM image with the color-coded STEM-EDS elemental maps of O, Ni, Mn, and Co for NMC811.


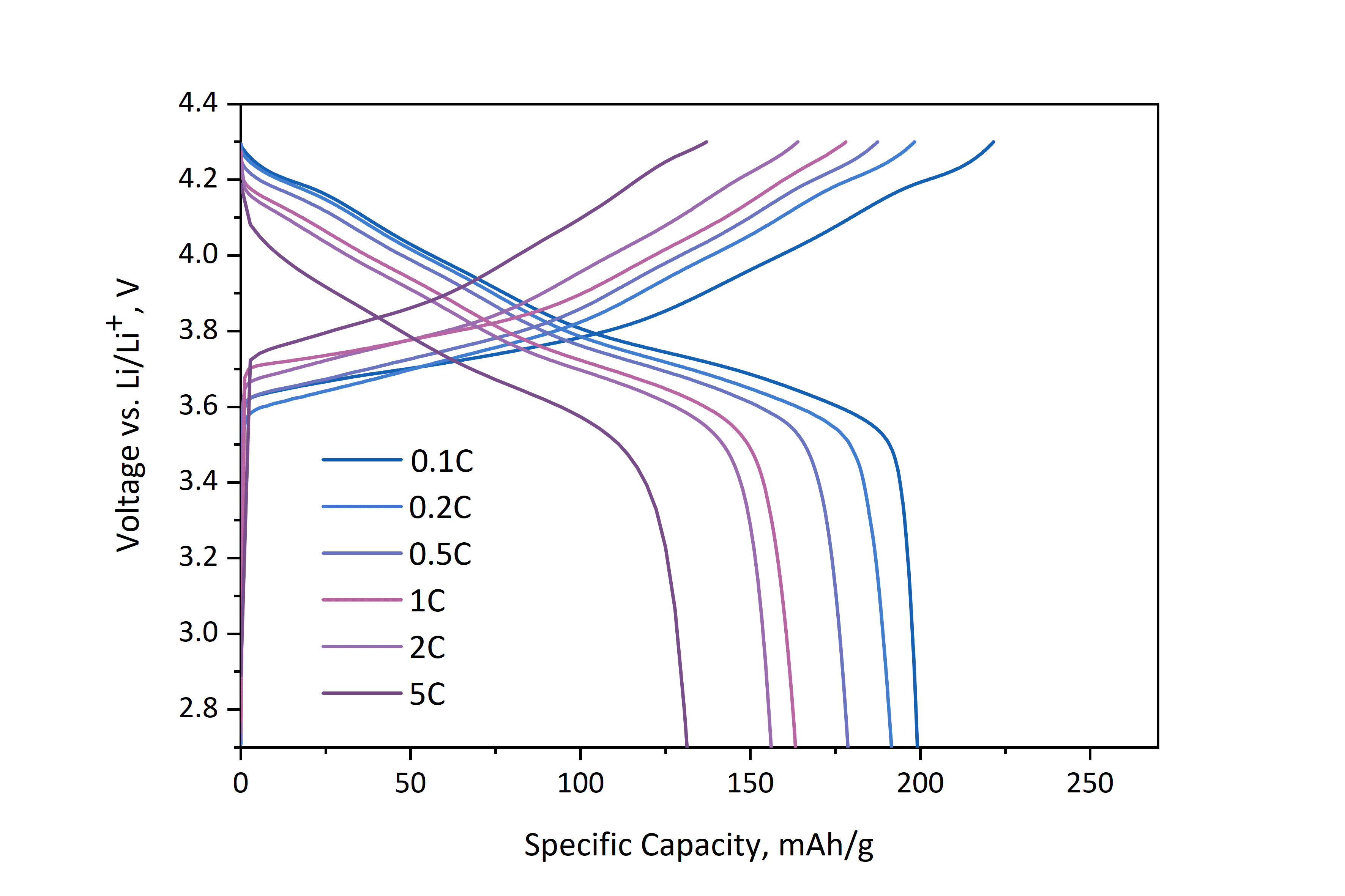


Figure S9. Galvanostatic charge-discharge curves at different current densities for NMC811 in half-cell with metallic Li anode (2.7 – 4.3 V vs Li/Li^+^).


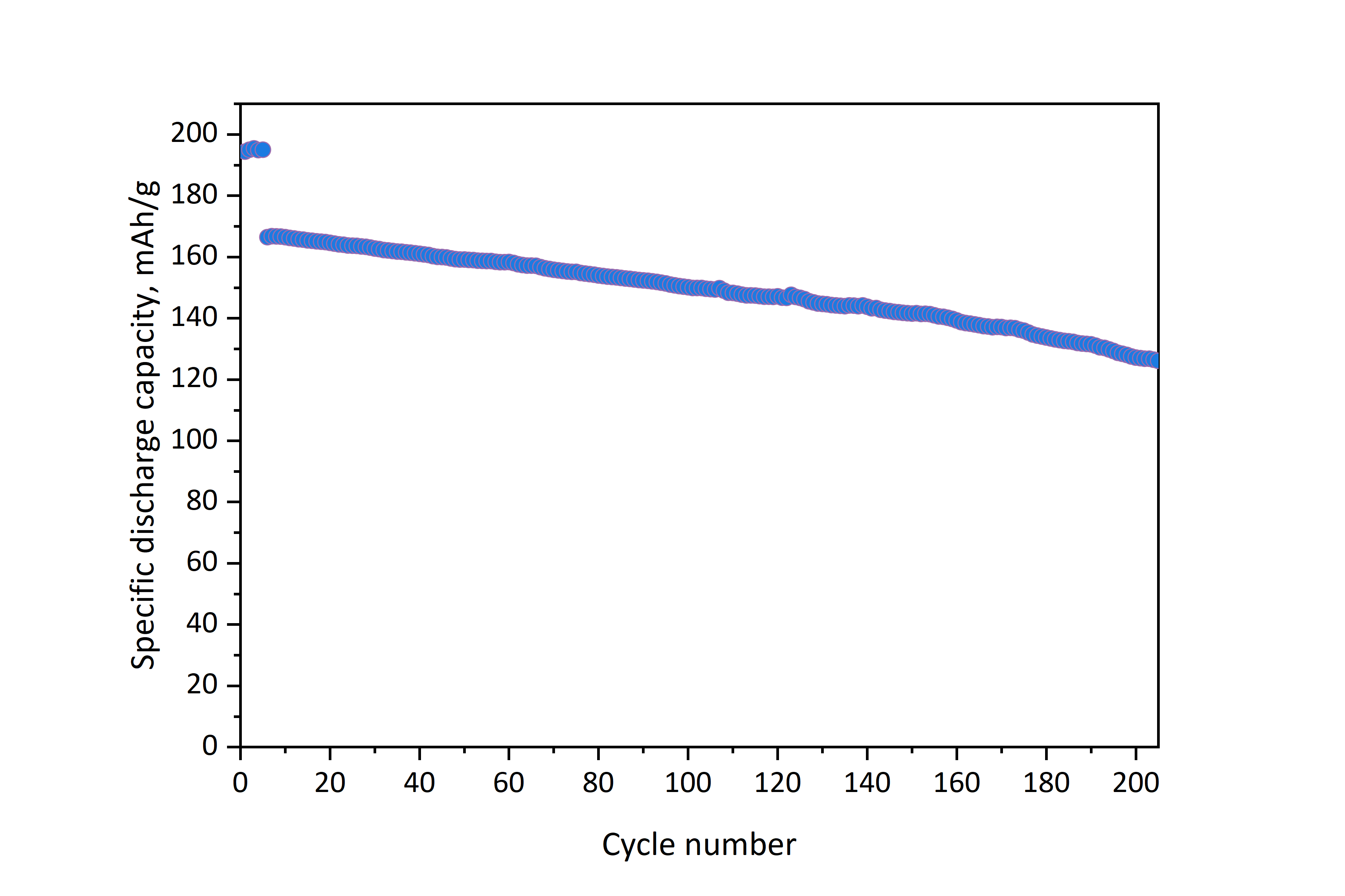


Figure S10. Capacity retention at 1C (2.7 – 4.3V vs Li/Li^+^) in half-cell with metallic Li anode for NMC811.


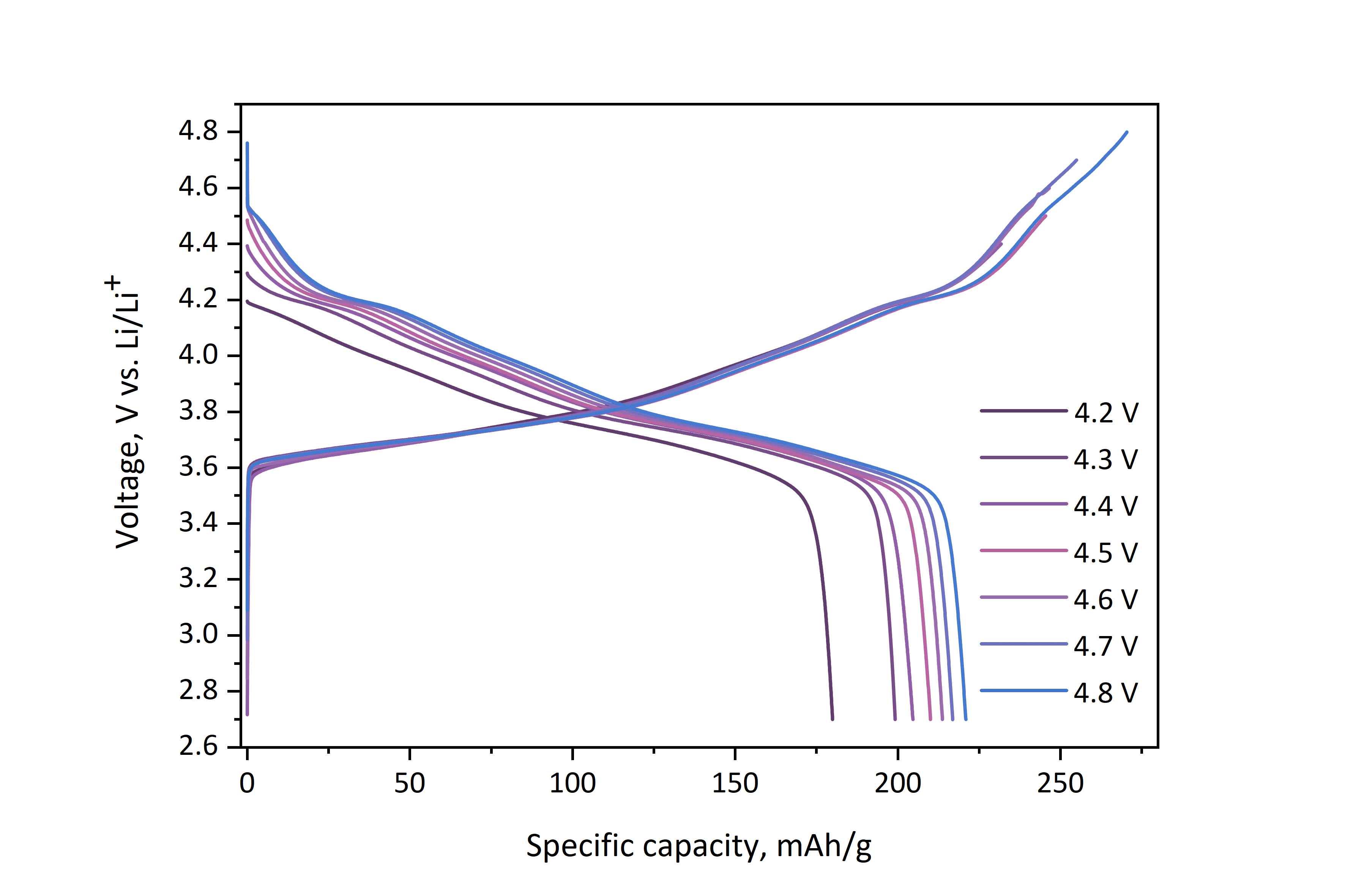


Figure S11. Galvanostatic charge-discharge curves of first cycle at different upper potential (0.1C current rate) for NMC811 sample in half-cell with metallic Li anode.

**References for Supporting Information.**

[1] H.J. Noh, S. Youn, C.S. Yoon, Y.K. Sun, Comparison of the structural and electrochemical properties of layered Li[Ni_x_Co_y_Mn_z_]O_2_ (x = 1/3, 0.5, 0.6, 0.7, 0.8 and 0.85) cathode material for lithium-ion batteries, J. Power Sources. 233 (2013) 121–130. https://doi.org/10.1016/j.jpowsour.2013.01.063.

[2] H.H. Ryu, K.J. Park, C.S. Yoon, Y.K. Sun, Capacity fading of Ni-rich Li[Ni_x_Co_y_Mn_1-x-y_]O_2_ (0.6 ≤ x ≤ 0.95) Cathodes for High-Energy-Density Lithium-Ion Batteries: Bulk or Surface Degradation?, Chem. Mater. 30 (2018) 1155–1163. https://doi.org/10.1021/acs.chemmater.7b05269.

[3] C. Pan, Y. Zhu, Y. Yang, H. Hou, M. Jing, W. Song, X. Yang, X. Ji, Influences of transition metal on structural and electrochemical properties of Li[Ni_x_Co_y_Mn_z_]O_2_ (0.6≤x≤0.8) cathode materials for lithium-ion batteries, Trans. Nonferrous Met. Soc. China. 26 (2016) 1396–1402. https://doi.org/https://doi.org/10.1016/S1003-6326(16)64244-9.

[4] X. Wang, X. Zhang, C. Zhang, L. Zhang, J. Wen, C. Wang, G. Huang, Synthesis of high-nickel and high-performance ternary cathode materials through spent lithium-ion batteries recycling system, Sustain. Chem. Pharm. 31 (2023) 100959. https://doi.org/https://doi.org/10.1016/j.scp.2022.100959.

[5] M. Hofmann, M. Kapuschinski, U. Guntow, G.A. Giffin, Implications of Aqueous Processing for High Energy Density Cathode Materials: Part I. Ni-Rich Layered Oxides, J. Electrochem. Soc. 167 (2020) 140512. https://doi.org/10.1149/1945-7111/abc033.

[6] E. Flores, P. Novák, U. Aschauer, E.J. Berg, Cation Ordering and Redox Chemistry of Layered Ni-Rich Li_x_Ni_1-2y_Co_y_Mn_y_O_2_: An Operando Raman Spectroscopy Study, Chem. Mater. 32 (2020) 186–194. https://doi.org/10.1021/acs.chemmater.9b03202.

[7] J. Oh, J. Kim, Y.M. Lee, D.O. Shin, J.Y. Kim, Y.-G. Lee, K.M. Kim, High-rate cycling performance and surface analysis of LiNi_1-x_Co_x/2_Mn_x/2_O_2_ (x=2/3, 0.4, 0.2) cathode materials, Mater. Chem. Phys. 222 (2019) 1–10. <https://doi.org/https://doi.org/10.1016/j.matchemphys.2018.09.076>.

[8] E.D. Orlova, A.A. Savina, S.A. Abakumov, A. V. Morozov, A.M. Abakumov, Comprehensive study of Li^+^/Ni^2+^ disorder in Ni-rich NMCs cathodes for Li-ion batteries, Symmetry (Basel). 13 (2021). https://doi.org/10.3390/sym13091628.

[9] V. Petrícek, M. Dušek, L. Palatinus, Crystallographic computing system JANA2006: General features, Zeitschrift Fur Krist. 229 (2014) 345–352. https://doi.org/10.1515/zkri-2014-1737.
